# The world’s user-generated road map is more than 80% complete

**DOI:** 10.1371/journal.pone.0180698

**Published:** 2017-08-10

**Authors:** Christopher Barrington-Leigh, Adam Millard-Ball

**Affiliations:** 1 Institute for Health and Social Policy; and School of Environment, McGill University, Montreal, Québec, Canada; 2 Environmental Studies Department, University of California Santa Cruz, Santa Cruz, California, United States of America; Johns Hopkins Bloomberg School of Public Health, UNITED STATES

## Abstract

OpenStreetMap, a crowdsourced geographic database, provides the only global-level, openly licensed source of geospatial road data, and the only national-level source in many countries. However, researchers, policy makers, and citizens who want to make use of OpenStreetMap (OSM) have little information about whether it can be relied upon in a particular geographic setting. In this paper, we use two complementary, independent methods to assess the completeness of OSM road data in each country in the world. First, we undertake a visual assessment of OSM data against satellite imagery, which provides the input for estimates based on a multilevel regression and poststratification model. Second, we fit sigmoid curves to the cumulative length of contributions, and use them to estimate the saturation level for each country. Both techniques may have more general use for assessing the development and saturation of crowd-sourced data. Our results show that in many places, researchers and policymakers can rely on the completeness of OSM, or will soon be able to do so. We find (i) that globally, OSM is ∼83% complete, and more than 40% of countries—including several in the developing world—have a fully mapped street network; (ii) that well-governed countries with good Internet access tend to be more complete, and that completeness has a U-shaped relationship with population density—both sparsely populated areas and dense cities are the best mapped; and (iii) that existing global datasets used by the World Bank undercount roads by more than 30%.

## Introduction

The world’s roads, and their extent and spatial distribution, have enormous implications for economic growth, urban development patterns, access to natural resources, and global climate change. Road transportation accounts for more than 80% of passenger travel [1] and nearly 20% of greenhouse gas emissions from fuel combustion [2]. Moreover, roads represent one of the most permanent commitments to how and where we will live in the future [3].

Accessible, complete, and accurate geospatial data on the world’s road network are therefore valuable not just for trip planning and navigation, but also for understanding questions as diverse as the drivers of deforestation [4] and urban poverty reduction [5]. Yet until recently, no global map nor global accounting of these roads existed. Google Maps and similar proprietary products do not permit geospatial analyses such as calculating road lengths. A new effort to map global roads—the Global Roads Open Access Data Set—meanwhile focuses only on inter-urban roads, and does not cover city streets [6].

Even basic cross-national data on the length of roads are lacking. One review from 1998 notes that the data derived from the International Road Federation (IRF) *World Road Statistics* [7] and UN statistical yearbooks “are patchy, with frequent gaps and many large changes that are often quickly reversed … it appears impossible to construct data that are consistent either across countries or over time” [8]. The extent to which the data have improved in recent years is unclear, and IRF’s sources for road network length for many countries are missing or incomplete.

OpenStreetMap, an ambitious open-data initiative that has emerged and grown rapidly in recent years, promises to fill this gap. Just as Wikipedia provides a volunteer-written encyclopedia, OpenStreetMap (OSM) provides a free, openly licensed, volunteer-contributed repository of geographic information. OSM launched in 2004 with a focus on streets and roads, and has subsequently expanded to map buildings, land uses, points of interest and other geographic features [9]. As of May 2017, ∼3.8 million contributors had created a database with ∼411 million roads, coastlines, administrative boundaries and other linear features known as “ways” [10]. Applications of OSM to date include humanitarian mapping following earthquakes, epidemics and other disasters [11], hydrological modeling [12], downscaling of population estimates to small geographic areas [13], research on diverse subjects from urban morphology to urban farming [14, 15], and even adult coloring books [16].

The usefulness of OSM for these purposes, however, depends on the completeness of the data and other aspects of data quality. As discussed later in this section, research has found that most Western countries which have been assessed appear to have a relatively complete road network in OSM. The picture in low-income countries, however, is much more uncertain. Researchers, policy makers, or citizens who want to make use of OSM road data, therefore, have little information about the extent upon which OSM can be relied. The absence of a global completeness assessment, meanwhile, hampers the use of OSM for research in economics, urban planning, environmental studies and related fields, such as analyses of worldwide patterns of travel behavior or urban development. Moreover, the benefits of OSM may be greatest in low-income countries where completeness is most uncertain, given the relative lack of official or commercial alternative geographic data products.

Most quality assessments of OSM and other Volunteered Geographic Information (VGI) datasets perform a comparison with an official government or proprietary reference dataset (e.g. [17, 18, 19, 20]). Normally, the length and position of the features in both datasets are compared, although there are other approaches such as comparing the output of routing algorithms (e.g. [21, 22]; for a more comprehensive review, see [23]).

Initially, researchers asked about the completeness of the OSM road network, the positional accuracy of the data, and the accuracy of attributes that indicate the type of road, speed limits, turn restrictions, and other information. Some studies continue to focus on completeness, for example through improving computational techniques that can compare OSM to a reference dataset [20]. However, by 2011, others had already noted that OSM research was shifting away from completeness assessments and towards the accuracy of attribute information, such as the opening times of points of interest [24]. More recently, studies have examined the quality of OSM data on building footprints [25], bicycle or pedestrian infrastructure [21, 26], points of interest [27], place names [28], and the classification of areal features [29].

This shift, however, may be somewhat premature, given that research has focused on Europe and North America, and the completeness of the OSM road network in most of the world is unknown. While early assessments found significant gaps [18, 19, 30, 31], more recent studies of European countries have found that the network is virtually complete, and is comparable to or better than official or proprietary data sources [17, 22]. The same does not appear to be true, however, in other parts of the world, such as China, Tehran and Brazil [32, 33, 34].

The only global effort that sheds light on the completeness in the OSM road network quantifies the number of changes to roads in a geographic area, and identifies where saturation has been reached, as defined by a growth rate of ≤ 3% for three or more years [35]. However, by focusing on the number of changes (new additions or edits), this approach does not distinguish between the addition of new roads versus minor edits that update attributes or make small improvements to positional accuracy, or between major versus minor additions. Moreover, this approach can say little about the completeness of areas that have not reached saturation. The definition of saturation in [35] is also restrictive; the authors find that only 11% of Europe (by land area) has reached saturation, even though the country-level studies noted above imply that completeness is likely to be much greater.

In addition to the lack of information on the level of completeness, there is little evidence that helps explain the considerable heterogeneity in completeness, and other aspects of data quality, between and within countries. Some countries and regions are better mapped than others, but the reasons are still unclear. In one U.S study, there is no detectable relationship between OSM data quality and demographic variables, possibly because such a small percentage of the population contributes to OSM, and because many edits are done by users who do not live locally [36]. European-focused studies have noted that dense areas appear to be better mapped in OSM [17, 19], presumably because there are more potential contributors with local knowledge. However, local contributions are only one manner through which the OSM database expands. Imports from official or proprietary data sources, and responses to humanitarian crises help to promote completeness [35]. OSM contributors also gather at “mapping parties” and other social events to make focused updates [37]. The most frequent contributors to OSM have contributed edits in more than one country, perhaps through tracing aerial imagery, or as a result of a vacation or other trip abroad [38].

In this paper, our objective is first to assess the completeness of the OSM road network, worldwide. We provide country-level estimates of completeness that are derived from two independent data sources. Note that we restrict our attention to completeness, a fundamental measure of geographic data quality, and do not assess positional accuracy or other measures commonly employed in the literature.

Second, we aim to shed light on the reasons for the global heterogeneity in completeness, and help explain why some geographic regions are more complete than others. Third, we provide new estimates of the total length of road for each country in the world, and offer a comparison between the OSM-derived roadway stock and official statistics and World Bank data.

## Methods

The simplest way to assess completeness, and the method used by most OSM completeness studies to date, is to compare the OSM database to a comparison dataset from an authoritative source. At the global scale of our analysis, however, no comparison dataset of real roads exists. Most lower-income countries have no readily available data from a national cartographic agency or similar organization. Commercial mapping products such as Google Maps have restrictive licenses, and may not be complete themselves in parts of the world. We therefore assess OSM completeness through two complementary approaches—(i) a visual comparison with aerial imagery, and (ii) fitting parametric models to the historical growth of the OSM street network.

Armed with our estimates of completeness, we then estimate the length of road network in each country, through dividing the existing length of mapped roads in OSM by our estimated fraction complete.

### Visual assessment

#### Sampling and assessment procedures

Our visual assessment is based on a stratified and probability-weighted sample of 45 points in each country. We implement our own sampling algorithm in the QGIS geographic analysis software to (i) select a random point and (ii) overlay streets in the OSM database against aerial or satellite imagery provided by Google through the OpenLayers plugin, at a scale of 1:5000. The number of missing street edges in the visible area (i.e., the screen view centered around the sampled point) is manually counted, and the script automatically counts the number of street edges already present in the OSM database. Here, we use the term “edge” in its graph-theoretic sense to denote the portion of a street between two nodes (intersections).

**Fig 1:**
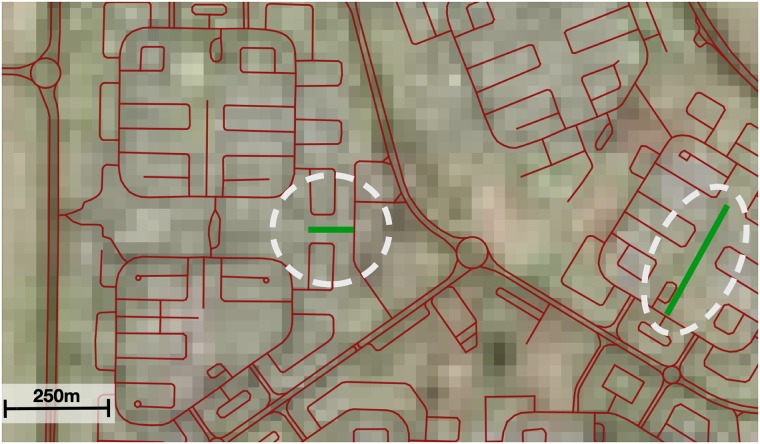
Example visual assessment. Street data from OSM is overlaid on satellite imagery of Kuwait City, Kuwait. Here, the network is 99% complete, with 2 out of 300 edges missing. The red lines indicate street edges in the OSM database. The green lines (highlighted with a white oval) are missing edges. While Google imagery was used in our actual inspection procedure of streets, lower-resolution public-domain imagery is shown in the figure. A version of this figure with the original imagery is available from the authors. Satellite imagery source: Landsat. Road network data source: OpenStreetMap.

The imagery includes streets from Google Maps, which aids in identifying roads where the image was low-resolution or obscured by trees. However, the main source is the actual aerial image, given that our observations indicate that the Google Maps data themselves are not complete in many parts of the world. An example is shown in Fig 1. In a small number of these cases we supplemented the Google imagery with the imagery from Bing, which is also available through the OpenLayers plugin. In order to focus our sampling efforts, we exclude 56 small dependencies, principalities and unrecognized countries, such as American Samoa, Greenland, Palestine and North Cyprus, which account for 0.2% of the global population.

Due to the geographic projection used, the size of the area included in a single observation varies with distance to the equator, but is approximately 1.2 km^2^ at 45 degrees latitude in France. In total, we assess 8370 observations, with a mean of ∼72 street edges in the OSM database and ∼10 missing edges per observation. The visual assessment was based on a February 2015 version of OSM. Therefore, we update the fraction complete to account for additions between February 2015 and January 2016. For example, if our February completeness estimate for a country was 60%, and road length grew by 10% between February and January, our updated estimate would be 66%.

Most of the land area of most countries is sparsely populated, but most roads are in urban areas. A simple random sample would be likely to exclude urban areas, while a sample limited to urban areas would ignore the lower-density areas where OSM may be less complete. Therefore, we adopt a two-part sampling approach with the aim of reducing the variance in our estimates.

The first sub-sample consists of a probability-weighted random sample of 25 points from each country, with selection probabilities proportional to the natural log of population density of each point. Population and density estimates are taken from the 2013 Landscan population distribution dataset, and population and density refer to that of the 30-arc second (∼1 km^2^) grid cell within which the sampled point lies [39]. Points with zero population are ignored. For the second sub-sample, we take a simple random sample of 20 points, restricted to densely populated areas where the point exceeds a country-specific density threshold.

The population density of rural areas varies considerably throughout the world, as does the definition of “urban.” In Canada, the United States and India, for example, places defined as urban must have a density of at least 400 persons km^-2^, while the density threshold is 150 persons km^-2^ in Malta, 500 in the Philippines and 1500 in China [40]. Therefore, we approximate the urban density threshold d* using $Pf=\sum_{i=1}^{N}p_{i}I(d_{i}\geq d^{*})$, where *P* is the population of a country, *f* is the fraction of population that is urbanized in that country (using World Bank data), *p*_*i*_ and *d*_*i*_ are the population and density of each point *i* obtained from the 2013 Landscan population distribution dataset [39], and *I*(⋅) is an indicator function. In the United States, for example, d* = 1165 persons km^−2^, while in India, d* = 11400 persons km^−2^.

Given our complex sampling design, we estimate the completeness of each country based on the inverse sampling probability-weighted totals of (i) OSM street edges (the numerator), and (ii) OSM plus missing street edges (the denominator). Confidence intervals are estimated via a nonparametric bootstrap procedure. We focus on the number of edges rather than road length, partly for feasibility of counting, and partly because edges are the natural units of additions to the OSM network. Since missing edges tend to be shorter than those already present in the OSM database (see Section 2 of the S1 Appendix), our results for “edge completeness” will underestimate the “length completeness” of OSM.

The analysts worked with a set of guidelines to ensure consistency in the definition of a road, and thus which edges were counted as missing. For example, driveways were ignored, as were unpaved paths leading to fields, and roads that are platted but have not yet been constructed. However, some degree of judgment was inevitable in the visual assessment. Although an exact match is not possible, the aim was to be as consistent as possible with the set of roads considered in the parametric modeling discussed below. When counting which edges were already included in the OSM database, only those tagged with the following highway tags were considered: *motorway*, *motorway_link*, *trunk*, *trunk_link*, *primary*, *primary_link*, *secondary*, *secondary_link*, *tertiary*, *residential*, *road*, *unclassified*, or *living_street*.

For example, driveways (excluded from the visual assessment) are generally tagged in OSM as *service* and would be excluded from the set of roads that we consider in the main analysis. Similarly, unpaved paths are generally tagged as *track* and would be similarly excluded.

#### Multilevel estimates of visual assessments

The bootstrapping procedure gives wide confidence intervals, because of the limited sample size within each country, and the wide variation in the number of edges and completeness across a country. To improve precision, we use a multilevel regression and poststratification (MRP) model [41], which draws on information from similar countries to provide tighter and more accurate confidence bounds than is possible when considering a country-level sample in isolation. Data are partially pooled across countries based on country-level covariates such as GDP and Internet access.

The MRP model has found particular relevance within political science and survey research, where its estimates are characterized by less error, higher correlations and lower variance [41, 42]. The MRP model has two further advantages beyond its statistical properties. It allows us to estimate the impacts of grid-cell density and the country-level covariates on the completeness of the OSM database. It also enables us to make out-of-sample estimates of completeness at the grid-cell level, not just at the country-level, and to illustrate the intra-country heterogeneity.

The first step of MRP is the multilevel regression, as in [43]. At the local (30-arc second grid cell) level, our predictor is population density. At the country level, our four predictor variables are GDP per capita (at purchasing power parity), Internet penetration (proportion of Internet users), population size, and the World Bank’s “voice and accountability” governance indicator, which “captures perceptions of the extent to which a country’s citizens are able to participate in selecting their government, as well as freedom of expression, freedom of association, and a free media” [44]. Population and GDP enter in log form. All country-level data are from the World Development Indicators and Worldwide Governance Indicators published by the World Bank [45], with imputation for countries with missing data. The full data set is provided as supplementary information.

Formally, for each observation *i* in country *j* ∈ {1, …, *m*}, we observe the number *S*_*ij*_ of road edges in the OSM database, and the real number of edges *T*_*ij*_, and estimate the following. At the first level:

$$T_{ij}\sim \text{Poisson}\left(\text{exp}\left(\beta_{j1}+\beta_{j2}\text{log}(d_{ij})+\beta_{j3}{\left(\text{log}(d_{ij})\right)}^{2}\right)\right)$$ (1)

$$S_{ij}\sim \text{Binomial}\left(T_{ij},\;f\left(\beta_{j4}+\beta_{j5}\text{log}(d_{ij})+\beta_{j6}{\left(\text{log}(d_{ij})\right)}^{2}\right)\right)$$ (2)

where *d*_*ij*_ is the local population density and *f* is the logit link function.

At the second level, the coefficients are drawn from a distribution as in Eq 3. Importantly, the coefficients are not a deterministic function of the country-level covariates, but rather are drawn from a distribution that is centered on those covariates:

$$\begin{array}{c} \beta_{j}∼N(\alpha +\gamma Z_{j},\;\Omega ) \end{array}$$ (3)

where *β*_*j*_, *α* are vectors of length 6 (given that there are six grid-cell coefficients for each country *j*, *β*_*j*1_ … *β*_*j*6_); γ, **Z**_*j*_ are *m*×6 matrices of coefficients and country-level covariates; and Ω is the variance-covariance matrix.

The model is estimated in a Bayesian framework using the open-source PyStan software [46]. We run the model for 10,000 iterations spread across ten independent chains. Half of the iterations are used for burn-in and the remainder are thinned to every fifth iteration, giving us a usable sample of 1,000 iterations. The Bayesian framework is primarily used for computational reasons, and our weak priors (Cauchy(0,2) based on standardized coefficients) are designed to help convergence rather than to incorporate prior information. Almost identical results are obtained from a weaker Cauchy(0,5) prior.

The second step of the MRP process is to apply the estimates out-of-sample to the entire globe. Based on the grid-cell level Landscan densities and the country-level coefficients *β*_*j*1_ … *β*_*j*6_, we estimate the number of road edges and the fraction complete in each 30-arc second grid cell. The country-level completeness estimates are then calculated as the mean completeness of each grid cell within that country, weighted by the estimated number of edges.

### Saturation of contributions

We employ a second, novel method of estimating completeness which relies only on details in the underlying OSM database itself. The total length of road mapped in a given region has a natural maximum. That is, the summed length of all roads in a region must converge to the actual extant length. Postulating that growth in road length in OSM is characterized in each country by growing interest at the beginning and saturation at the end, we approximate the time series of contributed length with a sigmoid shape. From the asymptote of the sigmoid, we infer the actual length of all roads. We are not the first to use a saturation criterion for the rate of changes; however, previously [35] an arbitrary threshold rate was used to indicate saturation (≤ 3% for each time interval over three or more years), while we allow for country-specific saturation levels to emerge from the model.

The OSM history dataset [47] provides a record of each version of each object in the OSM database, including objects that were subsequently deleted. The exceptions are objects whose original contributor did not agree to a license change in 2012; about 1% of data was lost as a result [48]. We use a custom Python script to extract every version in the contribution history of every node (i.e., each geolocated point) and every way (a linear sequence of nodes) that is tagged “highway,” which is a generic attribute for a roadway, including pedestrian paths and trails. We obtain the time stamp of each roadway (including its deletion date, if applicable), calculate its length, and identify the country where it is located using a spatial query against boundary data [49]. In this way, we build up a time series of the total road length rendered in each region. For the length calculation and the country lookup, we use a PostgreSQL/PostGIS spatial database. We provide our Python code under an open-source license (see S1 Appendix), allowing interested readers to replicate and/or update our findings.

In the main analysis in this paper, we restrict ourselves to roadways that are intended for vehicle circulation; these ways are further tagged *motorway*, *motorway_link*, *trunk*, *trunk_link*, *primary*, *primary_link*, *secondary*, *secondary_link*, *tertiary*, *residential*, *road*, *unclassified*, or *living_street*. However, we also show the growth in non-vehicle roadways, which largely consist of pedestrian paths. For clarity, we refer to “roads” and “other paths” in the remainder of this paper, where other paths are defined as roadways that do not have one of the above tags.

In order to estimate the growth and saturation of street coverage, we fit parametric models to the road length time series. While mostly monotonic, additions to road length are occasionally sudden, as opposed to steady. This is likely due to various kinds of bulk data imports (e.g. US government TIGER road data), the release of new aerial imagery which OSM contributors can trace [50], and “mapping parties” targeting localized areas. In order to accommodate these jumps, we use nonlinear least squares optimization to fit flexible functional forms which include up to four jumps superposed on a smooth sigmoid shape. From several such shapes as well as a linear growth model, we choose the best fitting functional form for each country, as measured by a mean-squared error criterion. These models are specified in detail in the S1 Appendix.

We follow the same process for two types of sub-national information. We fit parametric models to the road length time series at (i) the highest sub-national administrative level from GADM, such as U.S. states, German Länder and South African provinces; and (ii) each country-specific quintile of the distribution of grid-cell densities. We choose the best-fitting sigmoid functional form for each sub-national administrative unit and quintile. Incorporating subnational information in this way provides an independent check on the parametric fitting, in the sense that the sub-national asymptotes, as estimated from the fits, should add up to the country-level asymptote.

### Combining the estimates

We have two estimates of completeness for each country. The visual assessment is likely to be accurate but imprecise, while the parametric fit is precise but may detect a false saturation level (for example, due to a temporary hiatus in additions to the OSM database). We therefore combine the estimates as follows. In the 61 countries where the estimates match (i.e., the parametric estimate lies within the 95% confidence interval of the multilevel estimate, or where the difference between the estimates is 0.05 or less), we use the parametric fit. In the other 124 countries for which both estimates exist, we use the multilevel estimate derived from the visual assessment.

We also use the parametric fit in a further 68 countries, accounting for 0.3% of the global population, where no multilevel estimate is available. This is normally because we did not conduct the visual assessment for the reasons discussed in *Visual assessment*.

Our combined completeness estimates, coupled with the existing length of roads in the OSM database, provide the opportunity to make new estimates of the total length of the road network in each country, as in Eq 4. We exclude countries (all 5 of which are small-island states) where completeness is estimated at < 0.05.

$$\begin{array}{c} \text{roads}{}_{total}=\text{roads}{}_{OSM_{2016}}\times \text{frcComplete}{}_{OSM_{2016}}^{-1} \end{array}$$ (4)

## Results

We proceed by first presenting the results from our multilevel regression and poststratification model, which are based on the visual assessment. These results yield intrinsic insights into the reasons why completeness varies between and within countries, as well as allowing us to estimate the completeness of OSM in conjunction with the parametric fits. We present those completeness estimates in *Completeness estimates*, followed by our estimates of road length in *Quantifying road length*.

### Why countries get mapped

**Fig 2:**
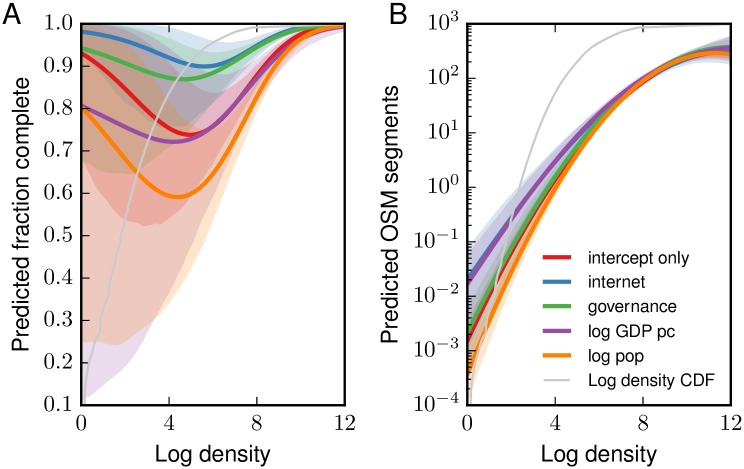
Predicted partial effects in multilevel model. The red line (intercept-only model) shows the baseline predictions, across the density spectrum, when all country-level variables are at their means. Each of the other lines shows the predicted fraction complete after a one standard deviation increase in one country-level predictor. 95% credible intervals are shaded. The thin grey line shows the cumulative distribution of grid-cell level densities in the world.

The standardized coefficients from the multilevel model are reported in the S1 Appendix, as are plots of the posterior densities of all coefficients. The structure of the model makes the coefficients difficult to interpret directly. For example, the impact of GDP affects completeness through both the intercept of the grid-cell level equation and interactions with local population density (in both log and log squared form), as can be seen in Eqs 3 and 2. Therefore, the most complete interpretation of the coefficients is given through a plot of the impact of each variable, as shown in panel A of Fig 2.

The most notable finding is that completeness has a ∪-shaped relationship with density. As shown in panel A of Fig 2, OSM is most likely to be complete at low and high densities. Thus, interurban roads that traverse areas with minimal population are largely present in OSM, and high-density urban areas, with many potential local contributors and good Internet access, are also well mapped. The types of communities that are most likely to have missing streets are smaller towns and villages.

Small countries tend to be more complete, as do those with more open governance and higher Internet penetration. GDP has no clear impacts on completeness, except at the lowest densities.

**Fig 3:**
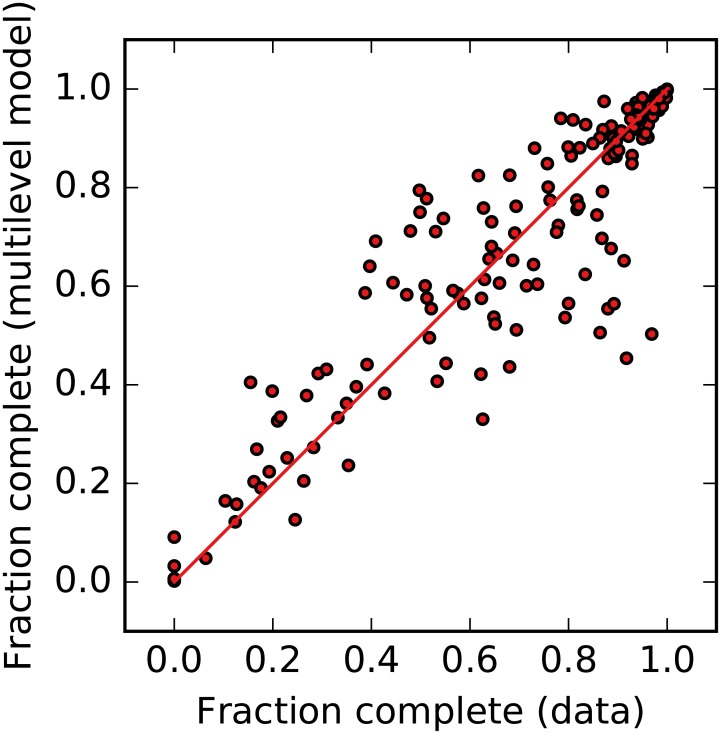
Visual assessments of completeness: Observations vs multilevel model. The two sets of estimates correlate well at the country level, with no evidence of bias, adding confidence to our model predictions. The multilevel estimates are obtained from poststratification using out-of-sample predictions for each grid cell in a country. The red line indicates equality (i.e., the 45° line).

One measure of the performance of our model is to compare the country-level predicted values with the raw estimates (weighted by inverse sampling probabilities) from the visual assessment. The estimates should not be identical, as the multilevel model draws strength from partial pooling with observations in “similar” countries, but should be correlated and mutually unbiased. Fig 3 indicates that this is the case.

Our multilevel model also provides estimates of the number of road edges, which we use to weight each grid cell when aggregating the grid-cell fraction complete predictions to the country level. As shown in panel B of Fig 2, the country-level predictors have little impact on the number of road edges, except at low densities. Unsurprisingly, density itself is a strong predictor of the number of roads. The standardized coefficients and plots of posterior densities are shown in the S1 Appendix.

### Completeness estimates

**Fig 4:**
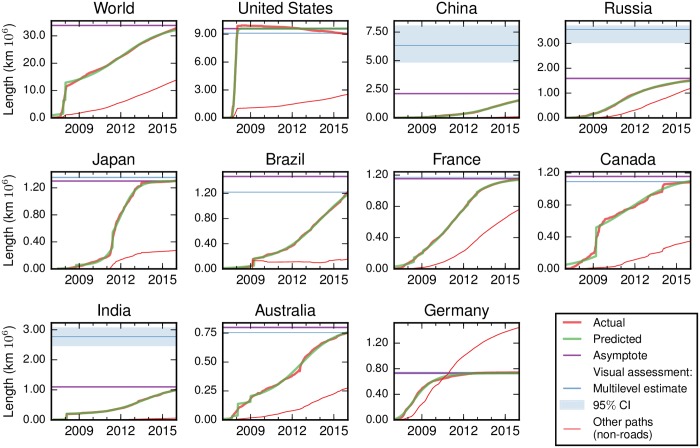
Growth in OSM dataset: Parametric fits and visual assessment. The largest ten countries by road length are shown, along with the global data. The S1 Appendix provides similar plots for all countries. The thick red line shows the actual data for roads, along with the predicted values, asymptote and visual assessment. The thin red line shows that other paths, which are mainly pedestrian routes, continue to grow in some countries even where the road network is complete. The decline in the US is mainly due to the bulk import of TIGER data, which has subsequently been cleaned (e.g. forest tracks are retagged as tracks rather than roads). Years shown indicate January 1.

Our multilevel model of the completeness of OSM suggests that it was ∼83% complete in January 2016, with a 95% confidence interval of 81%-84%. Close agreement is obtained from the country-level parametric fits, which give an estimate of 87% when summed to the global level and weighted by the estimated road length in each country. We also fit a sigmoid model to the growth in the global road stock (Fig 4), which suggests even greater completeness (97%). However, given that the figure suggests that recent growth at the global scale has been linear rather than sigmoid-shaped (Fig 4), we prefer the multilevel and country-level estimates. To reiterate, this measure of completeness only considers the presence of geographic features, and does not consider attribute information such as street names, nor other measures of data quality such as positional accuracy.

**Fig 5:**
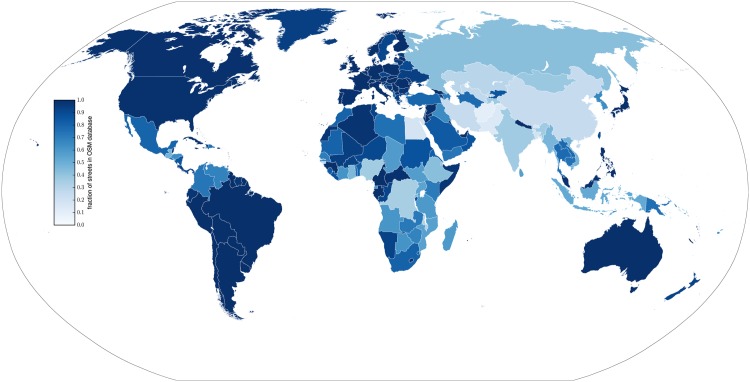
Completeness of the OSM dataset, by country, January 2016. The fraction complete is estimated by the parametric model, where that estimate falls within five percentage points or the 95% confidence interval of the multilevel model. Otherwise, the multilevel model is used.

Fig 4 shows the growth in the OSM database for the ten largest countries (by road length) and the corresponding parametric fit. The asymptote and multilevel model estimate (with confidence interval) are plotted as horizontal lines. Similar plots for all countries and World Bank-defined country groupings are provided in the S1 Appendix. Fig 5 shows the best completeness estimate for each country, generated by combining the visual assessment and parametric fit as described in *Combining the estimates*. Tables with the full estimates are in the S1 Appendix.

Of the 185 countries for which we have estimates from both the multilevel model and parametric fits, 77 are more than 95% complete. For 47 of them, our completeness has the highest level of confidence, in that the estimates from the multilevel model, country-level fits, and sub-national fits all indicate completeness of more than 95%. (Because the sigmoid model is asymptotic, 100% completeness is never achieved.)

**Fig 6:**
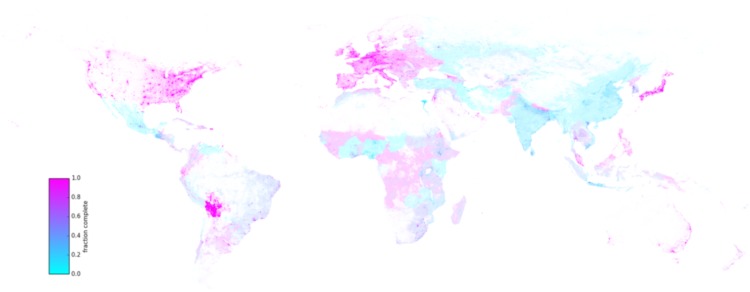
Completeness of the OSM dataset, by grid cell, January 2016. The fraction complete is estimated by the multilevel model. The color intensity represents the number of estimated street edges, thus highlighting parts of the world with a denser street network. The full-resolution image is available online.

There is considerable heterogeneity in estimated completeness. At one end of the spectrum, countries as varied as Kiribati, Afghanistan, Egypt and China are all less than one-third complete. There is also heterogeneity across the density gradient within countries, as shown in Fig 6.

**Fig 7:**
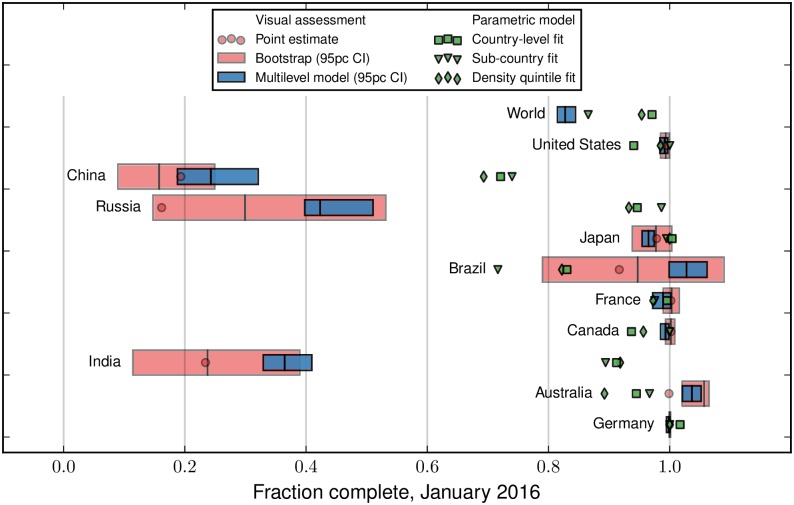
Comparison of methods. The largest ten countries by road length are shown, and a similar plot for all countries is provided in the S1 Appendix. The bars indicate the bootstrapped and multilevel model estimates from the visual assessment. The green makers indicate the estimates from the parametric fits at the country level, and by subnational density quintile and subnational administrative geography.

Fig 7 shows comparisons of the five completeness estimates for each country: (i) bootstrapping of the visual assessment; (ii) the multilevel model of the visual assessment; (iii) the country-level fits; (iv) the summation of fits for lower-level administrative geographies to the country level; and (v) a similar summation over density quintiles to the country level. In most of the countries where the estimates do not match, the OSM database is still growing rapidly, making it difficult to identify the saturation point through the parametric model. Brazil in Fig 4 provides a good example.

### Quantifying road length

**Fig 8:**
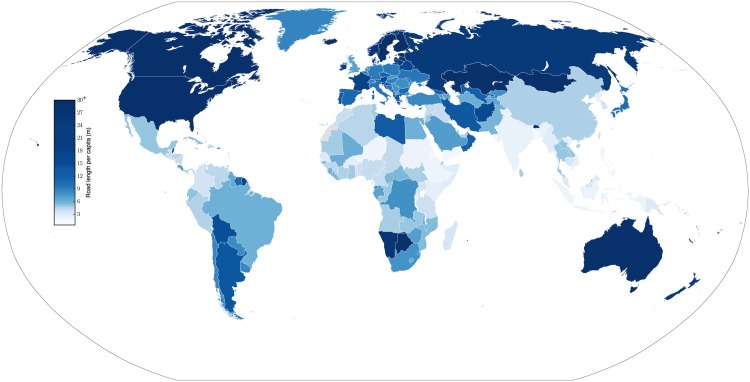
Road length per capita.

We find that the global stock of roads totals ∼39.7×10^6^ km, or ∼5.6 m per person. Interestingly, the United States accounts for ∼23.0% of the world’s stock of roads. Fig 8 shows the estimated road length per capita in each country in the world.

**Fig 9:**
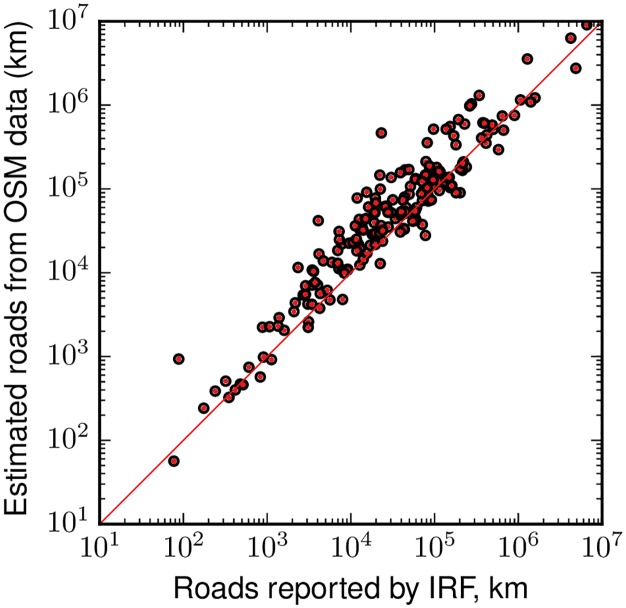
Road length data from the International Road Federation are substantially lower than OSM-based estimates. The red line indicates equality (i.e., the 45° line).

We also find that the concerns over the quality of the IRF and similar global roads statistics are well founded. Our OSM-based estimates of road length exceed those from the IRF in the majority of countries (Fig 9). In the world as a whole, our estimates are 134% of the IRF estimate. In 94 out of 190 countries, our estimate is more than 150% of the length reported by IRF. Note that our comparison is conservative, as the IRF totals include unpaved roads, and we drop countries where the IRF data specifically exclude local or urban roads.

Some of the discrepancy may be caused by double-counting of dual carriageways in the OSM database, where each side of a divided road is represented as a separate way. This is unlikely to account for more than a small part of the difference. One-way streets (a crude proxy for a dual carriageway) only account for 2.0% of the ways in the OSM database, as of May 2017 (see https://taginfo.openstreetmap.org/tags).

### Limitations

There are several sources of uncertainty and other limitations associated with our completeness estimates. Most importantly, contributions to OSM do not exhibit deterministic behavior. Thus, not only are our parametric fit functional forms heuristic, but our estimates have limited predictive power for future contributions in individual countries, which may occur in jumps. We also assume that urban growth is slow and that additions to the OSM database represent previously missing streets, whereas in reality the street network is growing in physical terms. The relatively small sample size in each country means that the confidence intervals from the visual assessment, meanwhile, are often wide. To some extent, however, these limitations are mitigated by our use of two independent methods.

## Discussion and conclusions

As the capabilities of Geographic Information Systems grow, and more spatial data becomes available through GPS receivers and official sources, there becomes an even greater need for publicly available sources of base data layers, particularly roads. While proprietary systems such as Google Maps may be suitable for trip planning and similar applications, they cannot be used for most research and analytic purposes. Our results show that in many parts of the world, OpenStreetMap (OSM) already fills that niche, and that about 42% of countries in the world are more than 95% complete. In other parts of the world, the OSM database is growing rapidly. At the global level, we find that the world’s road network is ∼83% complete (81%-84% with 95% confidence). Our results show that in many places, researchers and policymakers can rely on the completeness of OSM, or will soon be able to do so. In other regions, our results help to bracket the uncertainty.

Our results can be used to assess the fitness for purpose of OSM in individual countries, a contribution that is especially important given that there is wide variation even within low-income countries. At one extreme, we estimate that less than one-third of the streets in China, Egypt and Pakistan are in the OSM database, compared to more than 95% in Cuba, Ecuador and Syria as well as most European and North American countries.

Moreover, our methods can be used to track the country-by-country saturation of contributions, and identify the point at which more countries become complete. Because in many places OSM may now be the most authoritative data available even to local governments and agencies, better knowledge of its completeness is essential if it is to be relied on for planning and development purposes. In addition, knowledge of the completeness of the existing data can indicate where further mapping efforts should be directed, for example in emergency situations where humanitarian agencies already make significant use of OSM. For researchers, sufficient meta-knowledge including data completeness is necessary when using OSM road data for modeling of urban automobility and travel/transportation behavior, and local and climate-related emissions, among other outcomes in places where government or authoritative data are not readily available.

More broadly, our findings demonstrate a technique which may be generally useful for assessing the development and saturation of Volunteered Geographic Information and crowd-sourced data [35, 36, 51, 52]. Flexibly modeling a modified sigmoid curve can capture a variety of processes typical of user contributions, such as business listings, genetic databases, or encyclopedia and dictionary entries.

Equally importantly, we provide a new country-level dataset of road length that, unlike IRF’s *World Road Statistics*, is fully transparent and easy to update. Despite their advertised limitations, the IRF data are the basis for dozens of empirical papers in development economics [53], transportation [54, 55] and energy policy [56], and also appear to underlie other statistical compilations. In its World Development Indicators series, the World Bank sources the data on road length to IRF. The CIA World Factbook [57] does not cite individual sources, but the data are very similar to those published by the IRF. Thus, until now, there has been no obvious alternative to the IRF data.

Particularly in the poorest countries, we find that road supply is nearly 40% larger than suggested by IRF. In the world as a whole, our findings indicate a total road length of 39.7×10^6^ km or nearly 6 m per capita. Road length and road length per capita have important applications in the global study of economic development, transportation patterns, and pollution. For instance, using values of 5–25 kg CO_2_e year^−1^ km^−1^ [58] for life cycle emissions from petroleum-based and cementatious road surfaces, global annual emissions associated with the construction and maintenance of roads amortized over the lifetime of the road is on the order of 100–500 MT CO_2_e year^−1^. In places where completeness is already very high, changes in the OSM road database may even be used to indicate new road development.

Our findings also shed light on the factors that support the development of a crowd-sourced geographic database. Contributing to OSM requires access to the Internet, sufficient general resources such as education, geospatial expertise and leisure time to be able to contribute, and laws which permit the creation of non-government maps. In addition, the availability of open and accessible government information may facilitate importation of existing data to OSM. For example, most of the US road network was originally imported from the US Census Bureau TIGER files [59].

As expected, the most dense parts of the world have a relatively complete OSM network, likely because the most dense cities are home to many potential mappers. More surprisingly, we find a ∪-shaped relationship, with the best-mapped areas found at both ends of the density spectrum. In other words, not just the most dense but also the least dense areas are well mapped—perhaps because interurban roads are easy to trace from satellite imagery, or are already available through other sources. Consistent with intuition, we also find that countries scoring high on governance indicators and those with good Internet access tend to be more complete, and that small countries tend to be more complete than large ones. The open governance indicator may relate to the availability of geographic data, and even the ability of private citizens to undertake mapping efforts. China, for example, restricts private surveying and the publication of geospatial information.

Surprisingly, we find that income does not have a strong, independent effect on OSM completeness. There are also some notable outliers, such as Haiti and Nepal, where intense mapping efforts followed humanitarian disasters. Overall, however, the use of satellite imagery means that OSM contributors can be located in far-flung locations, and many contributions, in particular to sites targeted by humanitarian aid, are made from remote locations [37]. Thus, country-level factors have only limited predictive power and the wide confidence intervals as well as endogeneity concerns mean that our results here should not necessarily be given a causal interpretation.

A complete road network is only the first step in the development of an openly licensed geographic database. For some applications, the usability of OSM will depend on other aspects of data quality, such as positional accuracy, and the presence of tags that indicate road names, speed limits, and other attributes. In principle, our methods can be applied to these other metrics of data quality as well. For example, the percentage of streets that are named and have other attribute information should saturate over time. The same is true for OSM data on buildings, pedestrian paths and points of interest, and the reliability of the fitted curves can be complemented with a visual assessment. By quantifying the completeness of voluntary contributions to geographic information, the effort of the nearly 4 million contributors can be harnessed for broader purposes.

## Supporting information

S1 Appendix Further details on methods, results, and source data. A separate PDF outlines all associated resources. These resources are permanently available at https://alum.mit.edu/www/cpbl/publications/PLOS2017roads.
(PDF)
