# Correction: The world’s user-generated road map is more than 80% complete

**DOI:** 10.1371/journal.pone.0224742

**Published:** 2019-10-29

**Authors:** Christopher Barrington-Leigh, Adam Millard-Ball

## Notice of republication

An incorrect version of Fig 1 was published in error. This article was republished on October 18, 2019 to correct for this error. Please download this article again to view the correct version.
