# Supplementary material for: The world’s user-generated road map is more than 80% complete
Source: PLoS One. 2017 Aug 10;12(8):e0180698. doi: 10.1371/journal.pone.0180698 (PMC5552279; doi:10.1371/journal.pone.0180698)
Supplement: S1 Appendix — A separate PDF outlines all associated resources. These resources are permanently available at https://alum.mit.edu/www/cpbl/publications/PLOS2017roads. (PDF) [file pone.0180698.s001.pdf]

Supplementary Information:
The world's user-generated road map is more than 80% complete

Chris Barrington-Leigh and Adam Millard-Ball

These resources are permanently housed at:
`https://alum.mit.edu/www/cpbl/publications/PLOS2017roads`

Contents

A Database road length models **3**

B Sensitivity check: the lengths of missing edges **3**

C Table of estimated values **4**

D Comparison of fits by country **10**

E Parametric fits **17**

F Parametric fits by decile and world region **28**

G Multilevel model fits: table of standardized coefficients **68**

H Multilevel model fits: diagnostics and distribution **69**

I Data release **74**

J 2017 update **74**

K Open source Python code **74**

L Citation **75**

M Contact **75**

A Database road length models

We use the following functions to model the evolution of total mapped road length in each country (or region). In each case, y is the cumulative total street length in the database, while t represents the date, starting in March 2006. Except where specified in the case of Gompertz, all other parameters of each function are free parameters in the numerical optimization. Also, except for the linear function, which does not predict a saturation level nor date, $y_{\max}$ represents the predicted actual road length, towards which the road length in the OSM database converges.

Linear: This function is zero until some time t_0, after which it represents simple linear growth.

$$y(t;t_0,m) = I(t > t_0)m\,[t-t_0]$$

Here, and below, $I(\cdot)$ is an indicator function, giving value 1 when its argument is true and 0 otherwise.

Logistic function: This is the classic "sigmoid" curve.

$$y(t;k,y_{\max}) = y_{\max}\frac{1}{1+e^{-k[t-t_0]}}$$

Logistic with up to four jumps: We introduce up to four jumps superposed on the logistic curve.

$$y(t;t_0,k,y_{\min},y_{\max},\{t_i\},\{\delta_i\}) = y_{\min} + \sum_{i=1}^{4} I(t > t_i)\delta_i + \frac{\left[y_{\max} - y_{\min} - \sum_{i=1}^{4}\delta_i\right]}{1+e^{-k[t-t_0]}}$$

Here i ranges from 1 to 4 and the t_i are dates on which the road network length underwent a discontinuous increase. We also allow for 1, 2, or 3 jumps, rather than 4, as separate specifications.

Gompertz: The Gompertz function is another sigmoid curve which allows for asymmetry between the concave and convex regions.

$$y(t,t_0,t_1;y_{\max},b,c) = y_{\max}\exp\left(-b\exp\left(-c\frac{t-t_0}{t_1-t_0}\right)\right)$$

where t_0 and t_1 are fixed to be the first and last times in the time series.

The best fit is almost always obtained through the sigmoid functions with jumps. The linear fit produces the lowest mean-square error for just 1 country (Estonia), and the Gompertz function for 19 countries. The remaining countries are fit with a sigmoid function with no jump (2 countries), one jump (41), two jumps (44), three jumps (58), four jumps (84), or one ramp (4).

Where the model suggests that completeness is greater than one (i.e., the asymptote lies slightly below the maximum observed value), we code completeness as 1.0. In 22 countries, the model estimated completeness to be greater than 1.05. These are: Syrian Arab Republic, Ghana, Afghanistan, Burkina Faso, Malawi, Yemen, Haiti, Montenegro, Swaziland, New Caledonia, Gambia, Timor-Leste, Martinique, Cabo Verde, Brunei Darussalam, Barbados, Virgin Islands (U.S.), Dominica, Saint Vincent and the Grenadines, Kiribati, Liechtenstein, and Holy See. These countries tend to have experienced relatively recent rapid growth in the road network, and thus the parametric fit is likely to be less reliable—highlighting the value of using two independent methods to estimate completeness.

B Sensitivity check: the lengths of missing edges

We analyzed the length of existing vs missing edges formally for five randomly selected countries: Great Britain, Malta, Angola, French Guiana, and Djibouti. We compared the edge length of ways that existed in the OSM database on January 1, 2016, and compared that to a version of the OSM database from November 7, 2016. In both cases, we used the osm2po segmenter (osm2po.de) and restricted the database to the OSM tags described in the section "Saturation of contributions" of the main text. In each country, the average edge length became shorter over the $\sim$10-month period, implying that missing ways are also shorter. This makes sense given that longer edges are easier to include when tracing aerial imagery, and are less likely to be overlooked. Specifically, the reduction in length was 1.6% (Great Britain), 0.7% (Malta), 18.1% (Angola), 7.1% (French Guiana), and 11.2% (Djibouti). Note that the reduction in edge length is not solely due to newly added roads being shorter, but also arises when an existing edge is split by a newly added intersecting street.

C Table of estimated values

	Length		Fraction complete				
			Best	Multilevel model	Parametric fits		
Country	Total (10^3 km)	Per capita (m)			Country-level	From sub-geography	From quintiles
World	40000		0.83	0.83 (0.81–0.84)	0.97 (Logistic+2J)	0.87	0.95
United States	9100	29	0.99	0.99 (0.98–1.00)	0.94 (Logistic+1J)	1.00	0.98
China	6300	4.7	0.24	0.24 (0.19–0.32)	0.72 (Logistic+4J)	0.74	0.69
Russia	3600	25	0.42	0.42 (0.40–0.51)	0.95 (Gompertz)	0.99	0.93
India	2800	2.2	0.36	0.36 (0.33–0.41)	0.91 (Logistic+4J)	0.89	0.92
Japan	1300	10	1.00	0.97 (0.95–0.97)	1.00 (Logistic+3J)	0.99	1.00
Brazil	1200	6	1.00	1.03 (1.00–1.06)	0.83 (Logistic+1J)	0.72	0.82
France	1200	18	1.00	0.99 (0.97–1.00)	1.00 (Logistic+4J)	0.98	0.97
Canada	1100	31	0.99	0.99 (0.98–1.00)	0.94 (Logistic+1J)	1.00	0.96
Iran	1000	14	0.25	0.25 (0.27–0.23)	0.54 (Logistic+1J)	0.68	0.37
Pakistan	980	5.5	0.14	0.14 (0.13–0.16)	0.51 (Logistic+2J)	0.30	0.31
Australia	750	33	1.00	1.04 (1.02–1.05)	0.94 (Logistic+3J)	0.97	0.89
Germany	740	9.2	1.00	1.00 (0.99–1.00)	1.02 (Gompertz)	1.00	1.00
Nigeria	670	4	0.36	0.36 (0.31–0.42)	0.16 (Logistic+4J)	0.43	0.14
Mexico	620	5	0.80	0.80 (0.80–0.81)	0.87 (Logistic+3J)	0.48	0.37
Turkey	600	8.2	0.79	0.82 (0.81–0.82)	0.79 (Logistic+1J)	0.87	0.79
Argentina	600	14	1.00	1.03 (1.02–1.03)	0.92 (Logistic+1R)	0.88	0.91
Italy	580	9.7	1.00	0.98 (0.97–1.00)	1.01 (Logistic+4J)	0.98	0.97
DR Congo	560	7.9	0.34	0.34 (0.22–0.52)	0.75 (Logistic+4J)	1.00	1.00
Egypt	520	6	0.17	0.17 (0.16–0.19)	1.00 (Gompertz)	1.00	1.00
Kazakhstan	520	31	0.29	0.29 (0.22–0.39)	0.84 (Logistic+4J)	0.58	0.87
Indonesia	510	2.1	0.52	0.52 (0.51–0.55)	0.93 (Logistic+4J)	0.84	0.91
Spain	500	11	0.99	0.99 (0.97–1.00)	0.93 (Gompertz)	0.97	0.94
Afghanistan	470	16	0.10	0.10 (0.10–0.09)	1.32 (Logistic+1J)	0.17	1.00
United Kingdom	440	6.9	1.00	1.00 (0.99–1.00)	1.00 (Gompertz)	0.99	1.00
Ukraine	430	9.4	0.94	0.95 (0.89–1.00)	0.94 (Logistic+3J)	0.93	0.96
South Africa	410	7.7	0.81	0.81 (0.77–0.85)	1.00 (Logistic+3J)	1.00	1.00
Uzbekistan	360	12	0.26	0.26 (0.22–0.31)	0.68 (Logistic+4J)	0.81	0.70
Poland	350	9.2	1.00	1.01 (1.01–1.02)	0.92 (Logistic+4J)	0.96	0.98
Thailand	340	5	0.77	0.77 (0.73–0.82)	0.89 (Logistic+3J)	0.90	0.89
Sweden	290	31	0.91	0.94 (0.91–0.98)	0.91 (Gompertz)	0.94	0.83
Finland	210	39	1.00	1.01 (0.99–1.04)	1.02 (Logistic+3J)	0.98	1.00
Saudi Arabia	210	7.1	0.84	0.84 (0.79–0.89)	0.70 (Gompertz)	0.81	0.97
Viet Nam	200	2.3	0.47	0.47 (0.45–0.50)	0.92 (Logistic+2J)	0.90	0.93
Tanzania	190	3.9	0.59	0.59 (0.46–0.76)	1.02 (Logistic+2J)	0.82	0.31
Bangladesh	180	1.2	0.19	0.19 (0.15–0.24)	0.84 (Logistic+1J)	0.64	0.89
South Korea	180	3.6	0.64	0.64 (0.60–0.69)	0.99 (Logistic+4J)	0.97	0.92
Philippines	170	1.8	1.00	1.06 (1.03–1.09)	0.92 (Logistic+3J)	0.97	0.91
Mongolia	170	61	0.38	0.38 (0.35–0.55)	0.92 (Logistic+4J)	0.98	0.92
Ethiopia	170	1.8	0.42	0.42 (0.33–0.55)	0.86 (Logistic+4J)	0.84	0.87
Colombia	170	3.6	0.73	0.73 (0.68–0.81)	0.91 (Gompertz)	0.94	0.82
Algeria	160	4.4	0.98	0.98 (0.96–1.01)	0.85 (Logistic+4J)	0.69	0.79
Belarus	160	17	0.90	0.90 (0.83–0.95)	1.01 (Logistic+3J)	0.99	0.98
Romania	160	8.1	1.00	1.00 (0.98–1.02)	0.46 (Gompertz)	0.73	0.75
Greece	160	14	0.94	0.94 (0.92–0.96)	0.89 (Logistic+3J)	0.76	0.86

Continued on next page

Country	Length		Fraction complete				
			Best	Multilevel model	Parametric fits		
	Total (10^3 km)	Per capita (m)			Country-level	From sub-geography	From quintiles
Myanmar	160	3	0.47	0.47 (0.44–0.50)	0.80 (Logistic+3J)	0.78	0.85
Bolivia	150	14	1.00	1.12 (1.09–1.14)	0.37 (Gompertz)	0.76	0.76
Chile	150	8.5	1.00	1.00 (0.99–1.01)	1.01 (Logistic+4J)	0.98	0.99
Portugal	150	14	0.99	1.02 (1.01–1.03)	0.99 (Logistic+3J)	1.00	1.00
Peru	140	4.6	1.00	1.05 (1.03–1.07)	0.72 (Logistic+4J)	0.84	0.73
Mozambique	140	5.3	0.55	0.55 (0.45–0.70)	0.75 (Logistic+4J)	0.91	0.01
Netherlands	140	8.1	1.00	0.97 (0.96–0.98)	1.00 (Logistic+3J)	1.00	1.00
Iraq	130	4	0.65	0.65 (0.60–0.71)	0.04 (Logistic+2J)	0.30	0.16
Morocco	130	3.9	0.80	0.80 (0.79–0.82)	1.01 (Logistic+4J)	1.00	0.98
Austria	130	15	1.00	0.99 (0.99–1.00)	1.01 (Gompertz)	1.00	1.00
Venezuela	130	4.3	0.68	0.68 (0.69–0.68)	0.95 (Logistic+1J)	0.86	0.96
Norway	120	25	1.00	1.03 (1.00–1.05)	1.04 (Logistic+4J)	0.81	0.90
Ghana	120	4.9	0.45	0.45 (0.43–0.48)	1.06 (Logistic+1J)	0.95	0.98
Uganda	120	3.5	0.55	0.55 (0.44–0.67)	0.41 (Logistic+3J)	0.89	0.59
Czech Rep.	120	11	0.93	0.93 (0.89–0.96)	1.05 (Gompertz)	1.00	0.99
Kenya	110	2.6	0.71	0.71 (0.63–0.80)	0.51 (Logistic+4J)	0.92	0.78
Ireland	110	24	0.98	1.00 (1.00–1.01)	0.98 (Logistic+4J)	0.94	0.93
New Zealand	110	25	0.93	0.93 (0.88–0.97)	1.01 (Logistic+4J)	0.97	0.97
Angola	110	4.7	0.60	0.60 (0.59–0.62)	0.92 (Logistic+4J)	0.99	0.94
Belgium	1e+02	9.3	1.00	0.98 (0.98–0.99)	1.01 (Logistic+4J)	1.00	1.00
Zimbabwe	1e+02	7.1	0.68	0.68 (0.55–0.82)	0.88 (Logistic+1J)	0.95	0.42
Mali	99	6.2	0.94	0.94 (0.93–0.96)	0.88 (Logistic+3J)	0.68	0.95
Cote d'Ivoire	98	4.6	0.61	0.61 (0.60–0.62)	0.09 (Logistic+3J)	0.62	0.25
Denmark	97	17	0.99	1.00 (1.00–1.00)	0.99 (Logistic+3J)	1.00	1.00
Sri Lanka	96	4.7	0.57	0.57 (0.50–0.64)	0.94 (Logistic+3J)	0.42	0.63
Malawi	91	5.8	0.32	0.32 (0.26–0.39)	1.17 (Logistic+2J)	0.88	0.21
Hungary	90	9.1	1.00	1.00 (0.99–1.01)	0.90 (Gompertz)	0.99	0.92
Malaysia	90	3.1	0.99	0.99 (0.96–1.01)	0.83 (Logistic+3J)	0.95	0.86
Syria	88	4.1	1.00	1.03 (1.00–1.07)	1.09 (Logistic+4J)	0.89	1.00
Cameroon	86	4	1.00	1.08 (0.99–1.17)	0.00 (Logistic+3J)	0.53	0.34
Libya	85	14	0.75	0.75 (0.72–0.80)	1.03 (Logistic+4J)	1.00	1.00
Ecuador	80	5.2	0.98	1.02 (0.99–1.04)	0.98 (Logistic+4J)	0.98	0.97
Sudan	78	2.1	0.87	0.87 (0.82–0.94)	0.96 (Logistic+4J)	0.91	0.63
Bulgaria	78	11	0.97	0.97 (0.94–1.00)	0.92 (Logistic+3J)	0.83	0.94
Madagascar	74	3.3	0.59	0.59 (0.50–0.71)	1.03 (Logistic+3J)	0.94	0.99
Zambia	74	5	0.72	0.72 (0.64–0.86)	0.91 (Logistic+4J)	0.92	0.40
Taiwan	73		0.91		0.91 (Logistic+2J)	0.87	0.94
Switzerland	73	9.1	1.00	1.00 (0.99–1.00)	1.03 (Logistic+1J)	1.00	1.00
Lithuania	72	24	0.97	0.97 (0.94–0.99)	0.97 (Logistic+3J)	0.97	0.97
Nepal	68	2.5	1.00	2.46 (2.32–2.57)	0.09 (Logistic+4J)	0.03	0.22
Croatia	62	15	0.94	0.95 (0.92–0.98)	0.94 (Gompertz)	0.96	0.98
North Korea	62	2.5	0.66	0.66 (0.55–0.77)	0.96 (Logistic+2J)	0.99	0.96
Botswana	61	29	0.61	0.61 (0.57–0.67)	0.87 (Logistic+4J)	0.96	0.88
Azerbaijan	61	6.6	0.71	0.71 (0.66–0.78)	0.12 (Logistic+3J)	0.34	0.23
Guatemala	61	4	0.47	0.47 (0.42–0.53)	0.90 (Logistic+1J)	0.63	0.86
Namibia	60	26	0.93	0.93 (0.90–0.95)	0.24 (Logistic+2J)	0.91	0.33
Cuba	60	5.3	1.00	0.95 (0.94–0.97)	1.01 (Logistic+3J)	0.99	0.99

Continued on next page

Country	Length		Fraction complete				
			Best	Multilevel model	Parametric fits		
	Total (10^3 km)	Per capita (m)			Country-level	From sub-geography	From quintiles
Latvia	54	27	0.99	1.00 (0.98–1.03)	0.99 (Logistic+1J)	0.98	0.99
Georgia	54	12	1.00	0.97 (0.95–0.98)	1.01 (Logistic+1J)	1.00	1.00
Serbia	53	7.4	1.00	1.01 (0.99–1.03)	1.00 (Logistic+3J)	0.89	0.83
Chad	53	4.2	0.58	0.58 (0.54–0.64)	1.05 (Logistic+2J)	0.98	1.00
Tajikistan	52	6.6	0.37	0.30 (0.23–0.39)	0.37 (Logistic+4J)	1.00	0.27
Tunisia	52	4.8	0.93	0.91 (0.89–0.94)	0.93 (Logistic+4J)	0.97	0.94
Paraguay	52	8.2	1.00	1.01 (1.00–1.02)	1.00 (Logistic+4J)	0.86	0.99
South Sudan	52	4.7	0.58	0.58 (0.44–0.75)	0.22 (Logistic+3J)	0.77	0.91
Guinea	51	4.4	1.00	1.15 (1.08–1.22)	0.84 (Logistic+4J)	0.29	0.87
Cambodia	49	3.3	0.75	0.75 (0.67–0.88)	0.91 (Logistic+2J)	0.82	0.90
Estonia	45	34	0.96	0.96 (0.93–0.99)	(justaline)	0.81	0.83
Kyrgyzstan	44	7.9	0.85	0.85 (0.81–0.90)	0.94 (Logistic+1J)	0.90	0.94
Burkina Faso	44	2.7	0.93	0.93 (0.91–0.97)	1.23 (Logistic+4J)	0.78	1.00
Senegal	44	3.2	0.70	0.70 (0.65–0.77)	1.03 (Logistic+3J)	1.00	1.00
Dominican Rep.	43	4.3	0.76	0.76 (0.72–0.80)	0.89 (Logistic+1J)	0.99	1.00
Oman	42	12	0.86	0.86 (0.79–0.92)	0.66 (Logistic+4J)	0.84	0.89
UAE	42	4.7	1.00	1.00 (0.99–1.01)	0.93 (Logistic+3J)	0.94	0.95
Slovakia	41	7.6	1.00	0.96 (0.93–0.98)	1.01 (Logistic+1J)	1.00	1.00
Benin	40	3.9	0.82	0.82 (0.74–0.95)	0.31 (Logistic+1J)	0.66	0.90
Yemen	38	1.5	0.73	0.73 (0.70–0.77)	1.10 (Logistic+4J)	1.00	1.00
Bosnia & Herz.	37	9.6	0.97	0.94 (0.91–0.98)	0.97 (Logistic+2J)	0.98	0.95
Sierra Leone	36	6	0.84	0.81 (0.72–0.90)	0.84 (Logistic+2J)	0.98	0.94
Puerto Rico	35	9.6	0.99	0.95 (0.94–0.96)	0.99 (Logistic+2J)	1.00	1.00
Honduras	35	4.5	0.64	0.67 (0.64–0.71)	0.64 (Logistic+2J)	0.82	0.38
Costa Rica	34	7.2	0.93	0.93 (0.90–0.97)	1.03 (Logistic+4J)	1.00	1.00
Laos	33	5.1	0.78	0.76 (0.65–0.91)	0.78 (Logistic+4J)	0.90	0.86
Moldova	33	9.2	1.00	1.01 (1.00–1.03)	1.02 (Logistic+1J)	0.95	1.00
Rwanda	32	3	0.44	0.44 (0.41–0.46)	1.01 (Logistic+1J)	0.60	0.45
Turkmenistan	32	6.2	0.77	0.77 (0.70–0.86)	0.62 (Logistic+2J)	0.93	0.80
Jordan	31	4.9	1.00	0.96 (0.94–0.98)	1.03 (Logistic+4J)	1.00	1.00
Slovenia	31	15	1.00	1.05 (1.03–1.07)	0.90 (Gompertz)	0.62	0.87
Niger	30	1.7	0.91	0.91 (0.81–0.98)	1.03 (Logistic+1J)	0.93	1.00
Somalia	29	2.9	0.98		0.98 (Logistic+1J)	0.91	0.97
Israel	29	3.7	0.98	1.00 (0.99–1.01)	0.98 (Gompertz)	1.00	1.00
Albania	29	9.9	0.75	0.75 (0.71–0.80)	0.88 (Logistic+3J)	0.98	0.93
Uruguay	28	8.2	1.00	0.99 (0.98–1.00)	1.00 (Logistic+1J)	0.96	1.00
C. African Rep.	25	5.4	0.99	0.97 (0.94–1.00)	0.99 (Logistic+3J)	0.97	0.56
Togo	25	3.7	0.77	0.77 (0.76–0.79)	0.50 (Logistic+1J)	0.91	0.99
El Salvador	25	4.1	0.75	0.75 (0.69–0.80)	0.69 (Logistic+4J)	1.00	0.78
Nicaragua	24	4	0.86	0.86 (0.83–0.89)	0.47 (Logistic+2J)	0.66	0.89
Liberia	23	5.5	0.87	0.87 (0.82–0.92)	0.98 (Logistic+2J)	0.96	0.91
Bhutan	22	30	0.17	0.17 (0.12–0.25)	0.78 (Logistic+2J)	0.05	0.94
Armenia	22	7.4	0.94	0.94 (0.89–0.99)	0.94 (Logistic+4J)	0.92	0.96
Burundi	22	2.2	0.87	0.87 (0.81–0.93)	0.96 (Logistic+3J)	0.86	0.79
Papua New Guinea	22	3	0.75	0.57 (0.34–0.85)	0.75 (Logistic+4J)	1.00	0.78
Congo	21	5	0.92	0.92 (0.89–0.95)	0.58 (Logistic+2J)	0.38	0.62
Iceland	20	61	0.95	0.95 (0.91–0.98)	1.03 (Logistic+1J)	1.00	1.00

Continued on next page

Country	Length		Fraction complete				
			Best	Multilevel model	Parametric fits		
	Total (10^3 km)	Per capita (m)			Country-level	From sub-geography	From quintiles
Lebanon	18	4.2	0.79	0.79 (0.75–0.83)	0.97 (Logistic+1J)	0.26	0.60
Mauritania	18	4.8	0.80	0.86 (0.72–0.96)	0.80 (Logistic+3J)	0.90	0.83
Panama	17	4.6	0.94	0.89 (0.85–0.94)	0.94 (Logistic+4J)	0.94	0.96
Haiti	17	1.6	0.94	0.94 (0.93–0.96)	1.05 (Logistic+2J)	0.95	1.00
Macedonia	14	7	1.00	1.03 (1.02–1.04)	0.88 (Logistic+4J)	0.75	0.91
Palestine	14	3.4	1.00		1.01 (Logistic+4J)	1.00	1.00
Lesotho	13	6.5	0.99	0.99 (0.93–1.05)	0.59 (Logistic+4J)	0.32	0.45
Kosovo	13	7.2	0.94	0.92 (0.87–0.97)	0.94 (Logistic+4J)	0.94	1.00
Jamaica	13	4.7	1.00	1.07 (1.04–1.10)	0.59 (Logistic+3J)	0.49	0.50
Cyprus	12	11	0.97	0.99 (0.98–0.99)	0.97 (Logistic+2J)	1.00	1.00
Samoa	12	61	0.10	0.10 (0.01–0.22)	0.96 (Logistic+3J)	1.00	0.96
Kuwait	11	3.3	1.00	0.96 (0.94–0.97)	1.04 (Logistic+3J)	1.00	1.00
Gabon	11	6.8	1.00	1.11 (1.07–1.23)	0.30 (Logistic+2J)	0.30	0.80
Montenegro	11	17	1.00	1.07 (1.05–1.09)	1.08 (Logistic+2J)	0.99	0.99
Guinea-Bissau	11	6.3	0.27	0.27 (0.25–0.30)	0.79 (Logistic+4J)	0.80	0.83
Qatar	11	5.3	0.92	0.92 (0.91–0.93)	0.99 (Logistic+2J)	0.98	0.98
Swaziland	10	8.4	0.82	0.82 (0.74–0.89)	1.07 (Logistic+1J)	1.00	1.00
Trinidad	9.9	7.4	1.00	0.98 (0.97–0.99)	1.05 (Logistic+1J)	1.00	1.00
Gambia	7.7	4.2	0.61	0.61 (0.59–0.64)	1.08 (Logistic+4J)	1.00	1.00
Western Sahara	7.5		0.98		0.98 (Logistic+1J)	0.99	1.00
Eritrea	7.1	1.5	0.82	0.82 (0.77–0.86)	1.03 (Logistic+2J)	0.86	0.72
Fiji	7.1	8.1	0.70	0.70 (0.69–0.84)	0.99 (Logistic+3J)	0.99	1.00
Eq. Guinea	7	9	0.61	0.61 (0.49–0.81)	0.99 (Logistic+4J)	0.03	1.00
Luxembourg	6.2	12	1.00	0.99 (0.98–1.00)	1.03 (Logistic+4J)	1.00	0.98
Reunion	6.1		0.97		0.97 (Logistic+4J)	0.99	0.99
Suriname	5.7	11	0.97	1.00 (0.99–1.00)	0.97 (Logistic+2J)	0.97	0.99
Belize	5.5	16	1.00	0.98 (0.93–1.02)	1.00 (Logistic+2J)	1.00	1.00
Bahamas	5.4	15	1.00	1.03 (1.01–1.03)	1.02 (Logistic+4J)	0.92	0.99
Timor-Leste	5.1	4.4	0.69	0.69 (0.63–0.77)	1.05 (Logistic+3J)	0.99	1.00
Guyana	4.8	6.3	1.00	1.16 (1.14–1.17)	1.04 (Logistic+3J)	0.79	1.00
New Caledonia	4.7	18	1.00		1.05 (Logistic+4J)	1.00	1.00
N. Cyprus	4.6		0.99		0.99 (Logistic+3J)	1.00	1.00
Mauritius	4.4	3.5	0.71	0.71 (0.65–0.78)	0.97 (Logistic+3J)	0.97	1.00
Guadeloupe	4.3		1.00		1.04 (Logistic+2J)	1.00	1.00
Djibouti	4.2	5	0.42	0.42 (0.39–0.52)	0.97 (Logistic+2J)	1.00	0.68
Singapore	4.2	0.79	1.00	1.01 (1.01–1.01)	1.02 (Logistic+3J)	1.00	1.00
Bahrain	3.8	2.8	0.98	1.02 (1.01–1.02)	0.98 (Logistic+3J)	0.92	0.97
Hong Kong	3.4	0.48	0.98		0.98 (Logistic+1J)	0.98	0.98
Martinique	3.1		1.00		1.10 (Logistic+2J)	1.00	1.00
Solomon Isl.	2.9	5.3	0.51	0.51 (0.40–0.72)	1.02 (Logistic+4J)	0.99	1.00
Brunei	2.6	6.4	0.88	0.88 (0.84–0.92)	1.08 (Logistic+4J)	1.00	0.61
Cabo Verde	2.3	4.6	1.00	1.10 (1.08–1.11)	1.09 (Logistic+4J)	0.97	0.97
Vanuatu	2.3	9.2	1.00	1.49 (1.32–1.60)	0.87 (Logistic+4J)	0.78	0.81
Comoros	2.2	3.1	0.56	0.56 (0.35–0.82)	1.02 (Logistic+4J)	1.00	0.99
Malta	2.2	5.3	1.00	1.03 (1.03–1.04)	1.04 (Logistic+2J)	1.00	1.00
Barbados	2.1	7.3	1.00		1.14 (Logistic+2J)	1.00	1.00
Fr. Polynesia	1.9	7.1	1.00		1.00 (Logistic+3J)	1.00	1.00

Continued on next page

Country	Length: Total (10^3 km)	Length: Per capita (m)	Fraction complete: Best	Fraction complete: Multilevel model	Parametric fits: Country-level	Parametric fits: From sub-geography	Parametric fits: From quintiles
Fr. Guiana	1.9		1.00		1.03 (Logistic+2J)	1.00	1.00
Curacao	1.7	11	0.99		0.99 (Logistic+2J)	0.99	0.95
Guam	1.7	10	1.00		1.01 (Logistic+2J)	1.00	1.00
Aland Islands	1.5		1.00		1.02 (Logistic+4J)	0.96	1.00
US Virgin Isl.	1.5	14	1.00		1.06 (Logistic+4J)	1.00	1.00
St. Kitts & Nevis	1.4	27	0.41		0.41 (Logistic+2J)	1.00	1.00
Falkland Isl.	1		0.98		0.98 (Logistic+1J)	0.98	0.99
Saint Lucia	0.99	5.5	0.93		0.93 (Logistic+4J)	1.00	0.40
Dominica	0.98	14	1.00	1.14 (1.14–1.15)	1.06 (Logistic+4J)	1.00	1.00
Aruba	0.98	9.6	0.95		0.95 (Logistic+3J)	0.95	1.00
Isle of Man	0.98	11	1.00		1.02 (Logistic+1R)	1.00	1.00
Maldives	0.93	2.7	1.00	1.40 (1.37–1.43)	1.05 (Logistic+4J)	1.00	1.00
Faroe Islands	0.92	19	1.00		1.03 (Logistic+1J)	1.00	1.00
Grenada	0.92	8.7	0.94	0.98 (0.95–1.00)	0.94 (Logistic+1J)	0.81	0.94
N. Mariana Isl.	0.76	14	0.94		0.94 (Logistic+4J)	0.00	0.98
Cayman Isl.	0.74	13	1.00		1.01 (Logistic+4J)	0.95	0.99
Akrotiri Dhekelia	0.67		0.96		0.96 (Logistic+3J)	1.00	0.99
Jersey	0.63		0.98		0.98 (Logistic+1J)	0.93	0.96
Turks & Caicos	0.62	19	1.00		1.00 (Logistic+3J)	0.02	1.00
St. Vincent	0.57	5.2	1.00		1.12 (Logistic)	1.00	1.00
Bonaire	0.57		0.92		0.92 (Logistic+3J)	1.00	1.00
Mayotte	0.55		1.00		1.02 (Logistic+4J)	1.00	1.00
Sao Tome	0.51	2.8	0.99		0.99 (Logistic+2J)	0.99	0.94
Greenland	0.49	8.6	0.94		0.94 (Logistic+3J)	1.00	0.79
Guernsey	0.48		1.00		1.02 (Logistic+1J)	1.00	1.00
Bermuda	0.47	7.2	1.00		1.02 (Logistic+4J)	0.99	1.00
Seychelles	0.46	5.2	1.00	1.05 (1.01–1.09)	1.00 (Logistic+3J)	0.02	0.97
Andorra	0.43	5.4	1.00		1.05 (Logistic+4J)	1.00	0.98
Macao	0.4	0.72	1.00		1.04 (Logistic+3J)	1.00	0.99
Micronesia	0.39	3.7	1.00	1.07 (1.03–1.09)	0.92 (Logistic+4J)	0.96	1.00
Liechtenstein	0.37	9.9	1.00		1.06 (Logistic+1J)	0.00	1.00
Am. Samoa	0.36	6.5	0.87		0.87 (Logistic+4J)	0.89	0.13
Br. Virgin Isl.	0.34		0.98		0.98 (Logistic+2J)	0.93	0.95
San Marino	0.32	10	1.00		1.00 (Gompertz)	0.50	0.86
Cook Isl.	0.31		1.00		1.05 (Logistic+3J)	1.00	0.98
Palau	0.3	14	1.00	1.01 (1.01–1.01)	1.00 (Logistic+4J)	0.91	0.96
Anguilla	0.24		0.90		0.90 (Logistic+4J)	0.90	0.91
Wallis & Futuna	0.22		0.98		0.98 (Logistic+3J)	0.98	1.00
St. Maarten	0.22	6.3	0.95		0.95 (Logistic+3J)	0.95	0.92
St. Helena	0.21		1.00		1.01 (Logistic+1J)	1.00	1.00
Marshall Isl.	0.18	3.4	1.00	1.01 (1.00–1.02)	1.02 (Logistic+4J)	1.00	0.92
St. Martin	0.18	5.8	1.00		1.01 (Logistic+3J)	1.00	0.93
Montserrat	0.14		1.00		1.01 (Logistic+4J)	1.00	0.90
St. Pierre	0.13		0.95		0.95 (Logistic+1J)	1.00	0.99
Niue	0.13		1.00		1.00 (Logistic+4J)	1.00	1.00
Svalbard	0.12		0.76		0.76 (Logistic+3J)	0.76	0.87
St. Barthelemy	0.1		1.00		1.05 (Logistic+4J)	1.00	0.97

Continued on next page

Country	Length		Fraction complete				
			Best	Multilevel model	Parametric fits		
	Total (10^3 km)	Per capita (m)			Country-level	From sub-geography	From quintiles
US Outlying Isl.	0.08		0.52		0.52 (Logistic+4J)	0.02	
Christmas Isl.	0.08		1.00		1.03 (Logistic+2J)	1.00	0.25
Norfolk Isl.	0.079		0.99		0.99 (Logistic+2J)	0.99	0.97
Gibraltar	0.073		0.97		0.97 (Logistic+4J)	0.97	0.94
Br. Ind. Ocean Terr.	0.068		0.94		0.94 (Logistic+3J)	0.94	
Monaco	0.056	1.5	0.98		0.98 (Logistic+3J)	0.98	0.94
Tuvalu	0.052	5.2	1.00	1.39 (1.38–1.39)	0.81 (Logistic+2J)	0.00	0.80
Nauru	0.046		0.95		0.95 (Logistic+4J)	1.00	0.76
Cocos Isl.	0.021		1.00		1.02 (Logistic+2J)	1.00	0.94
Fr. S. Terr.	0.0065		0.93		0.93 (Logistic+1J)	1.00	
Holy See	0.0058		1.00		1.05 (Logistic+2J)	1.00	0.68
Paracel Isl.	0.0056		0.98		0.98 (Logistic+4J)	0.98	
Tokelau	0.004		0.63		0.63 (Logistic)	0.00	0.00
Spratly Isl.	0.00064		1.00		1.00 (Gompertz)	1.00	
Antigua			0.00	0.00 (0.00–0.01)	1.05 (Logistic+1J)	0.94	0.84
Kiribati			0.04	0.04 (0.00–0.09)	1.14 (Logistic+1J)	1.00	1.00
Pitcairn			0.05		0.05 (Logistic+1R)	0.05	0.06
S. Georgia			0.00		0.00 (Logistic+1R)	0.00	
Tonga			0.01	0.01 (0.00–0.02)	0.82 (Logistic+4J)	0.99	0.74

D Comparison of fits by country

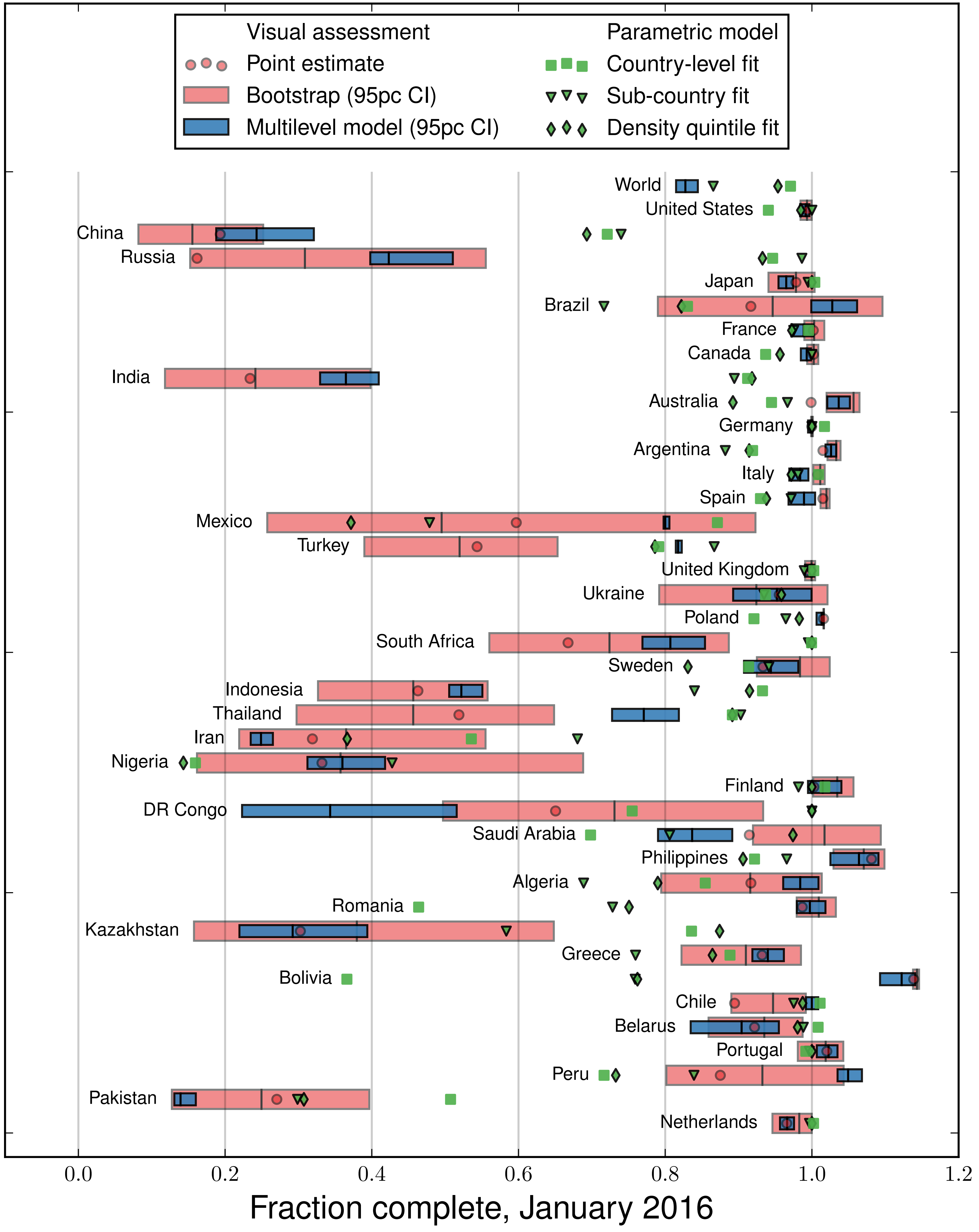

Visual assessment
Point estimate
Bootstrap (95pc CI)
Multilevel model (95pc CI)
Parametric model
Country-level fit
Sub-country fit
Density quintile fit
World
United States
China
Russia
Japan
Brazil
France
Canada
India
Australia
Germany
Argentina
Italy
Spain
Mexico
Turkey
United Kingdom
Ukraine
Poland
South Africa
Sweden
Indonesia
Thailand
Iran
Nigeria
Finland
DR Congo
Saudi Arabia
Philippines
Algeria
Romania
Kazakhstan
Greece
Bolivia
Chile
Belarus
Portugal
Peru
Pakistan
Netherlands
0.0
0.2
0.4
0.6
0.8
1.0
1.2
Fraction complete, January 2016

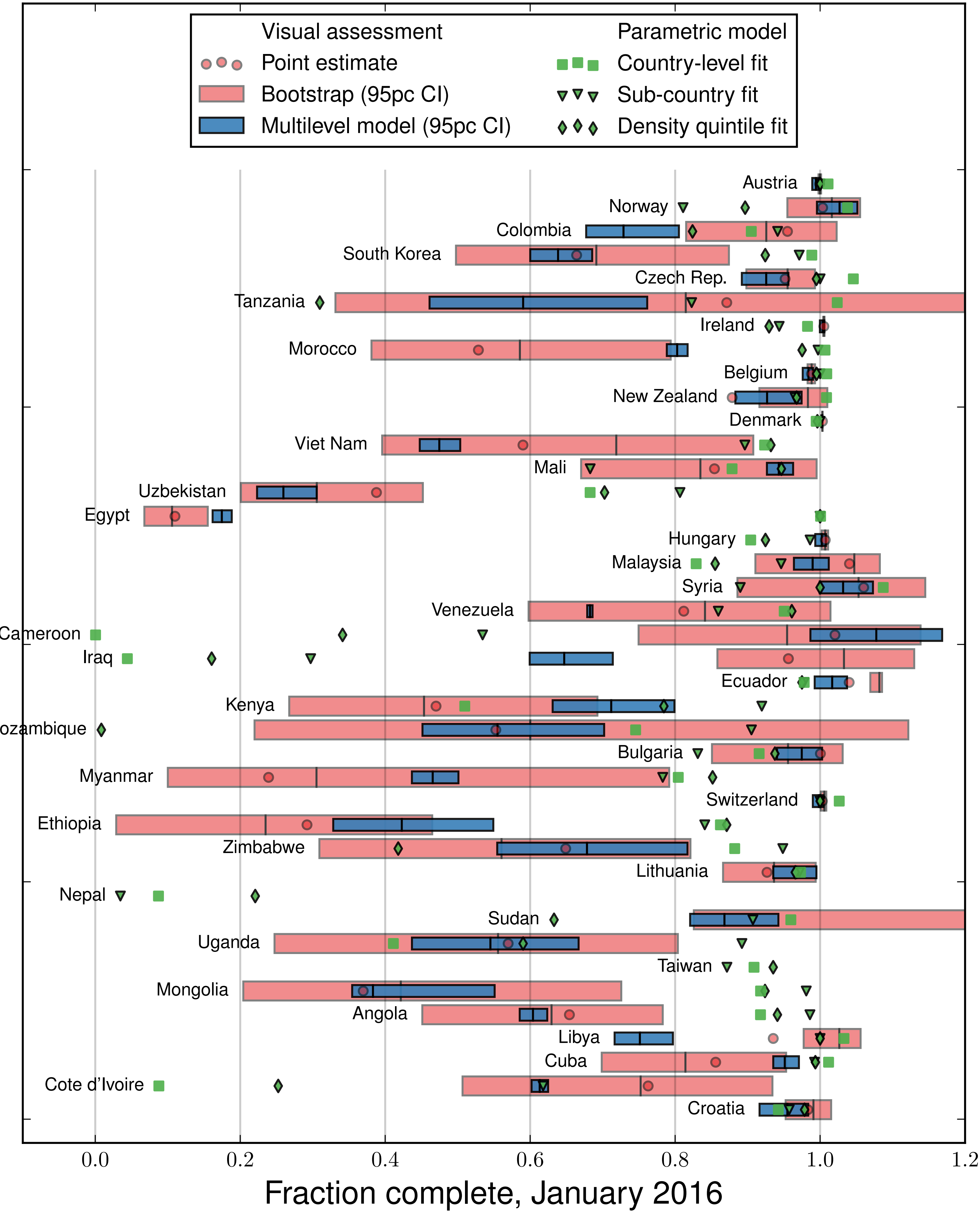

Visual assessment
Point estimate
Bootstrap (95pc CI)
Multilevel model (95pc CI)
Parametric model
Country-level fit
Sub-country fit
Density quintile fit
Austria
Norway
Colombia
South Korea
Czech Rep.
Tanzania
Ireland
Morocco
Belgium
New Zealand
Denmark
Viet Nam
Mali
Uzbekistan
Egypt
Hungary
Malaysia
Syria
Venezuela
Cameroon
Iraq
Ecuador
Kenya
ozambique
Bulgaria
Myanmar
Switzerland
Ethiopia
Zimbabwe
Lithuania
Nepal
Sudan
Uganda
Taiwan
Mongolia
Angola
Libya
Cuba
Cote d'Ivoire
Croatia
0.0
0.2
0.4
0.6
0.8
1.0
1.2
Fraction complete, January 2016

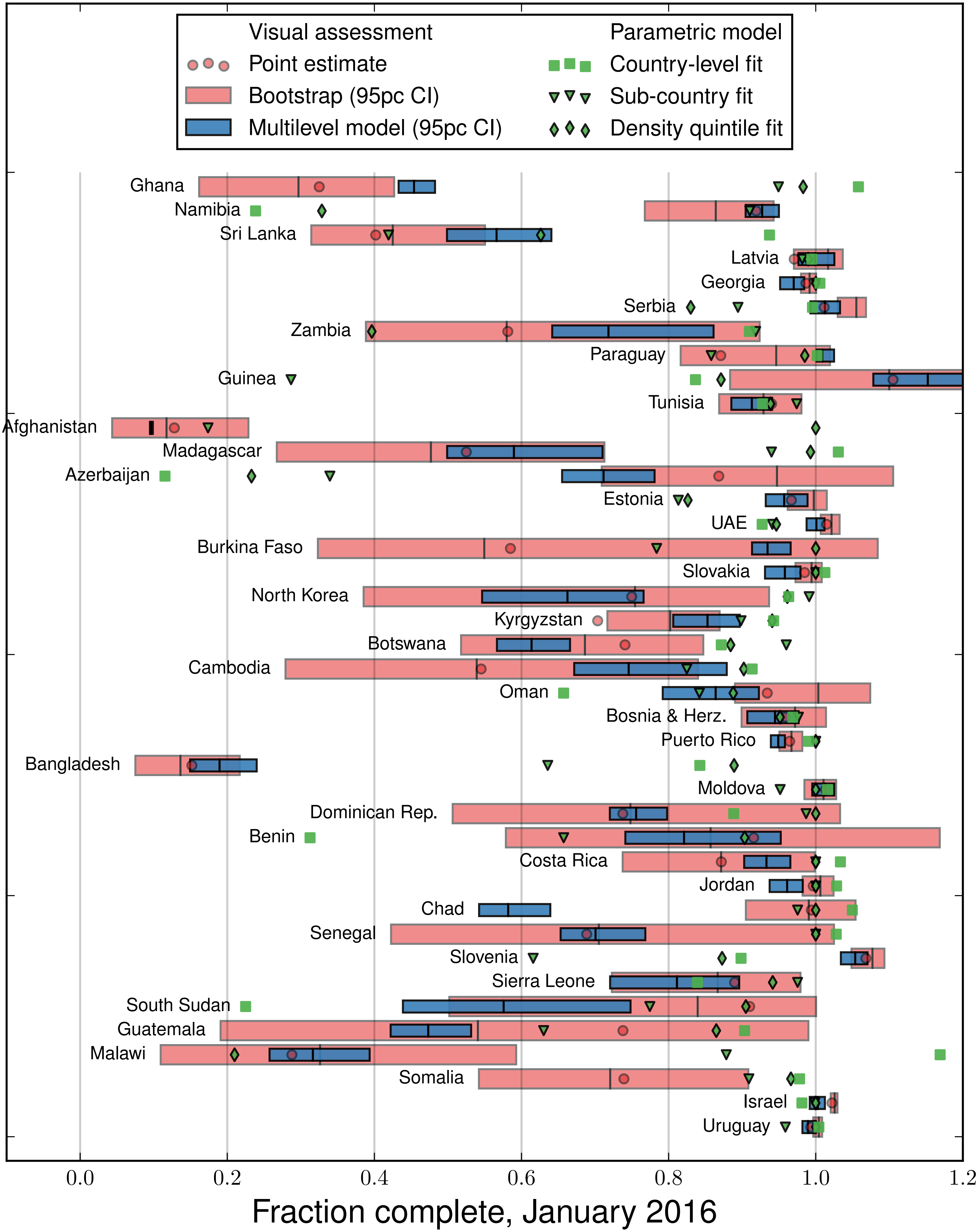
Visual assessment
Point estimate
Bootstrap (95pc CI)
Multilevel model (95pc CI)
Parametric model
Country-level fit
Sub-country fit
Density quintile fit
Ghana
Namibia
Sri Lanka
Latvia
Georgia
Serbia
Zambia
Paraguay
Guinea
Tunisia
Afghanistan
Madagascar
Azerbaijan
Estonia
UAE
Burkina Faso
Slovakia
North Korea
Kyrgyzstan
Botswana
Cambodia
Oman
Bosnia & Herz.
Puerto Rico
Bangladesh
Moldova
Dominican Rep.
Benin
Costa Rica
Jordan
Chad
Senegal
Slovenia
Sierra Leone
South Sudan
Guatemala
Malawi
Somalia
Israel
Uruguay
0.0
0.2
0.4
0.6
0.8
1.0
1.2
Fraction complete, January 2016

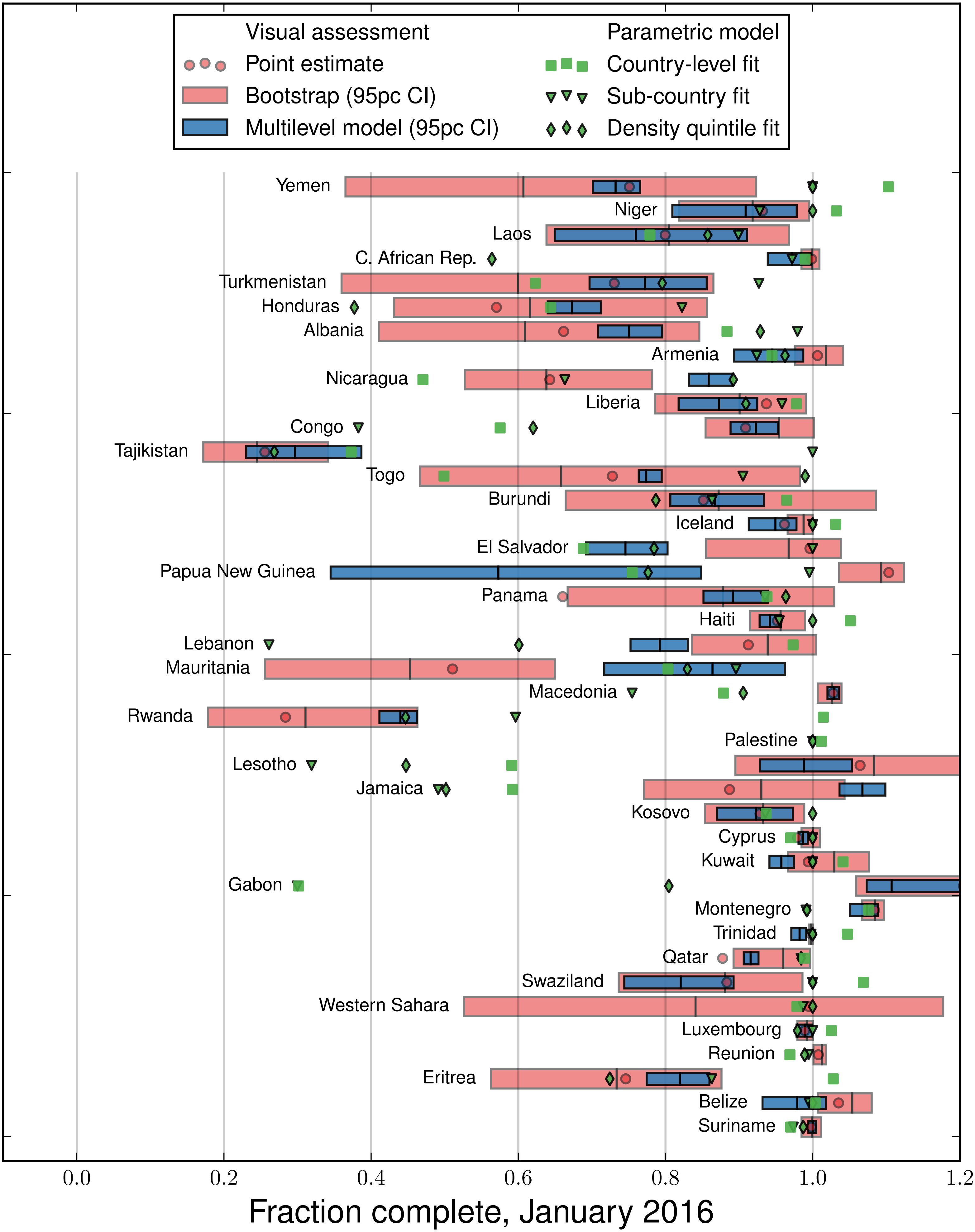
Visual assessment
Point estimate
Bootstrap (95pc CI)
Multilevel model (95pc CI)
Parametric model
Country-level fit
Sub-country fit
Density quintile fit
Yemen
Niger
Laos
C. African Rep.
Turkmenistan
Honduras
Albania
Armenia
Nicaragua
Liberia
Congo
Tajikistan
Togo
Burundi
Iceland
El Salvador
Papua New Guinea
Panama
Haiti
Lebanon
Mauritania
Macedonia
Rwanda
Palestine
Lesotho
Jamaica
Kosovo
Cyprus
Kuwait
Gabon
Montenegro
Trinidad
Qatar
Swaziland
Western Sahara
Luxembourg
Reunion
Eritrea
Belize
Suriname
0.0
0.2
0.4
0.6
0.8
1.0
1.2
Fraction complete, January 2016

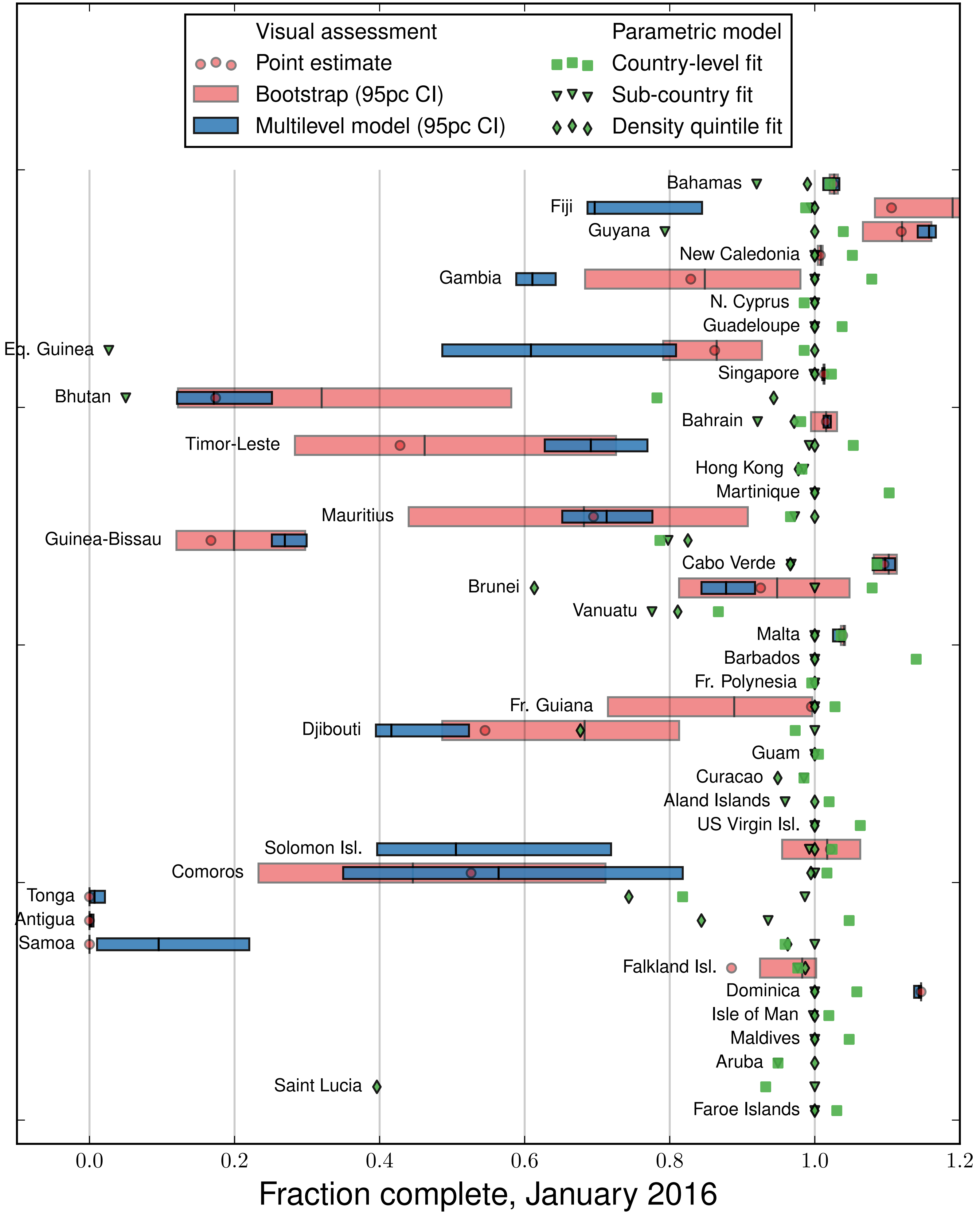
Visual assessment
Point estimate
Bootstrap (95pc CI)
Multilevel model (95pc CI)
Parametric model
Country-level fit
Sub-country fit
Density quintile fit
Bahamas
Fiji
Guyana
New Caledonia
Gambia
N. Cyprus
Guadeloupe
Eq. Guinea
Singapore
Bhutan
Bahrain
Timor-Leste
Hong Kong
Martinique
Mauritius
Guinea-Bissau
Cabo Verde
Brunei
Vanuatu
Malta
Barbados
Fr. Polynesia
Fr. Guiana
Djibouti
Guam
Curacao
Aland Islands
US Virgin Isl.
Solomon Isl.
Comoros
Tonga
Antigua
Samoa
Falkland Isl.
Dominica
Isle of Man
Maldives
Aruba
Saint Lucia
Faroe Islands
0.0
0.2
0.4
0.6
0.8
1.0
1.2
Fraction complete, January 2016

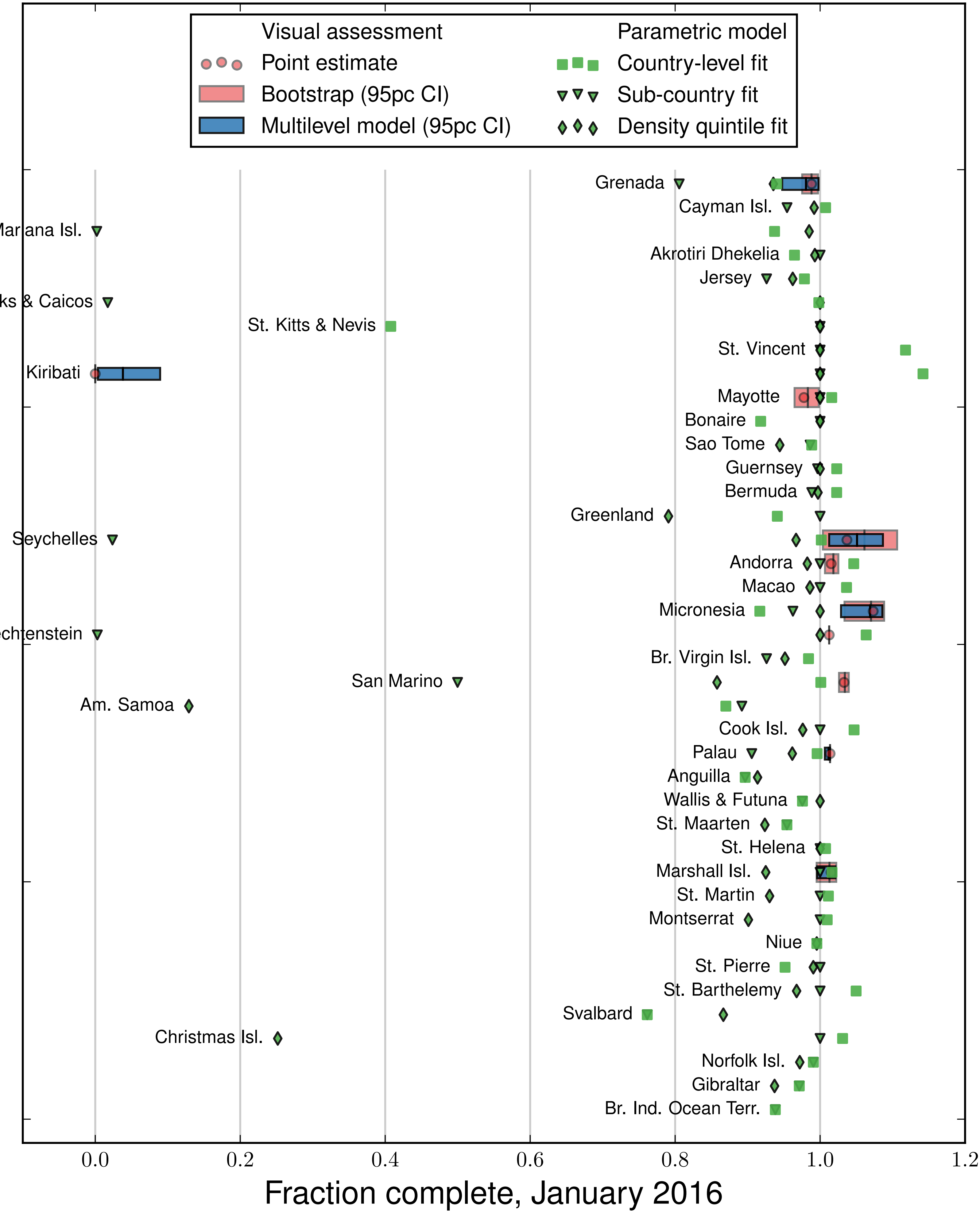

Visual assessment
Point estimate
Bootstrap (95pc CI)
Multilevel model (95pc CI)
Parametric model
Country-level fit
Sub-country fit
Density quintile fit
Grenada
Cayman Isl.
ana Isl.
Akrotiri Dhekelia
Jersey
ks & Caicos
St. Kitts & Nevis
St. Vincent
Kiribati
Mayotte
Bonaire
Sao Tome
Guernsey
Bermuda
Greenland
Seychelles
Andorra
Macao
Micronesia
chtenstein
Br. Virgin Isl.
San Marino
Am. Samoa
Cook Isl.
Palau
Anguilla
Wallis & Futuna
St. Maarten
St. Helena
Marshall Isl.
St. Martin
Montserrat
Niue
St. Pierre
St. Barthelemy
Svalbard
Christmas Isl.
Norfolk Isl.
Gibraltar
Br. Ind. Ocean Terr.
0.0
0.2
0.4
0.6
0.8
1.0
1.2
Fraction complete, January 2016

E Parametric fits

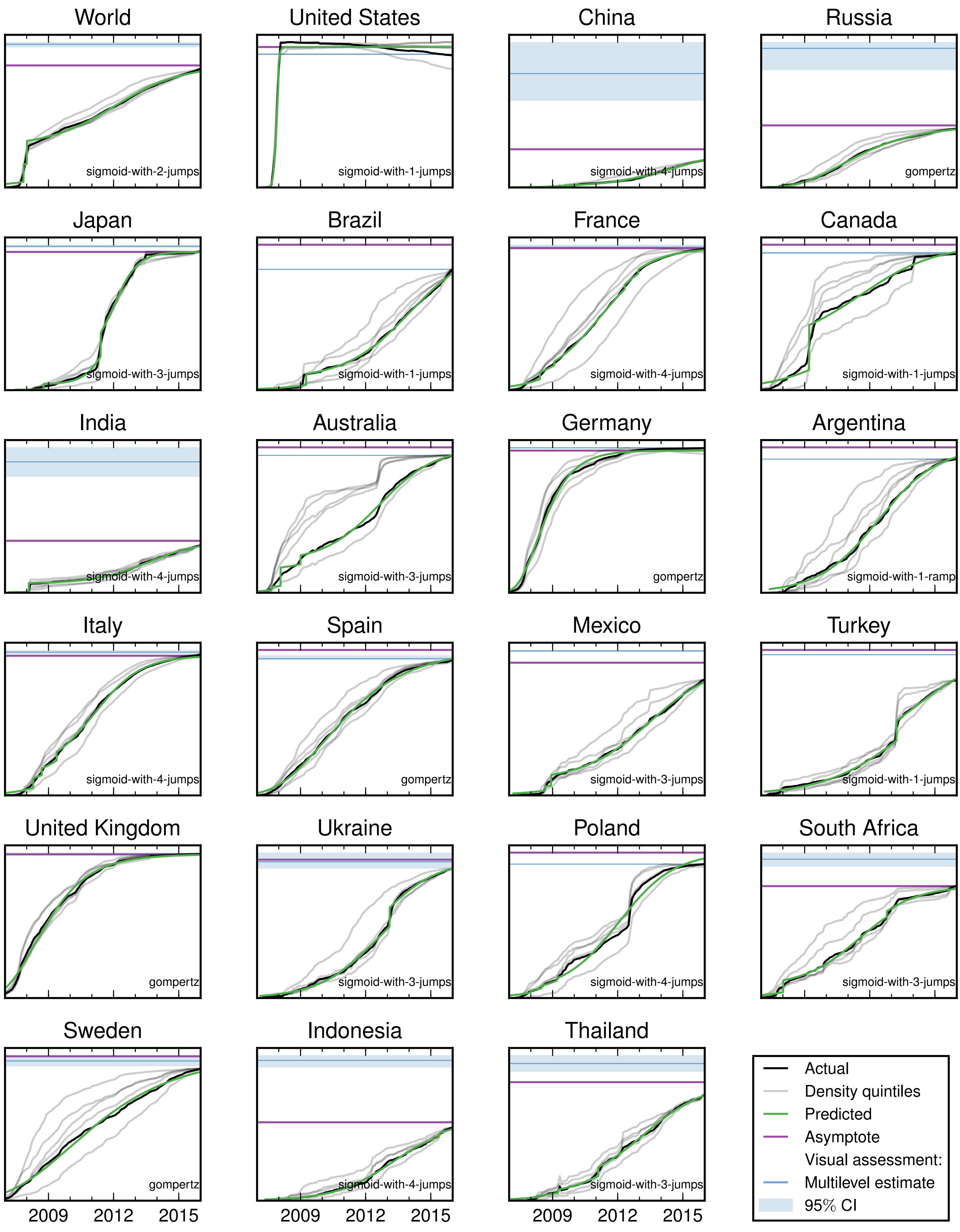
World
sigmoid-with-2-jumps
United States
sigmoid-with-1-jumps
China
sigmoid-with-4-jumps
Russia
gompertz
Japan
sigmoid-with-3-jumps
Brazil
sigmoid-with-1-jumps
France
sigmoid-with-4-jumps
Canada
sigmoid-with-1-jumps
India
sigmoid-with-4-jumps
Australia
sigmoid-with-3-jumps
Germany
gompertz
Argentina
sigmoid-with-1-ramp
Italy
sigmoid-with-4-jumps
Spain
gompertz
Mexico
sigmoid-with-3-jumps
Turkey
sigmoid-with-1-jumps
United Kingdom
gompertz
Ukraine
sigmoid-with-3-jumps
Poland
sigmoid-with-4-jumps
South Africa
sigmoid-with-3-jumps
Sweden
gompertz
Indonesia
sigmoid-with-4-jumps
Thailand
sigmoid-with-3-jumps
2009
2012
2015
Actual
Density quintiles
Predicted
Asymptote
Visual assessment:
Multilevel estimate
95% CI

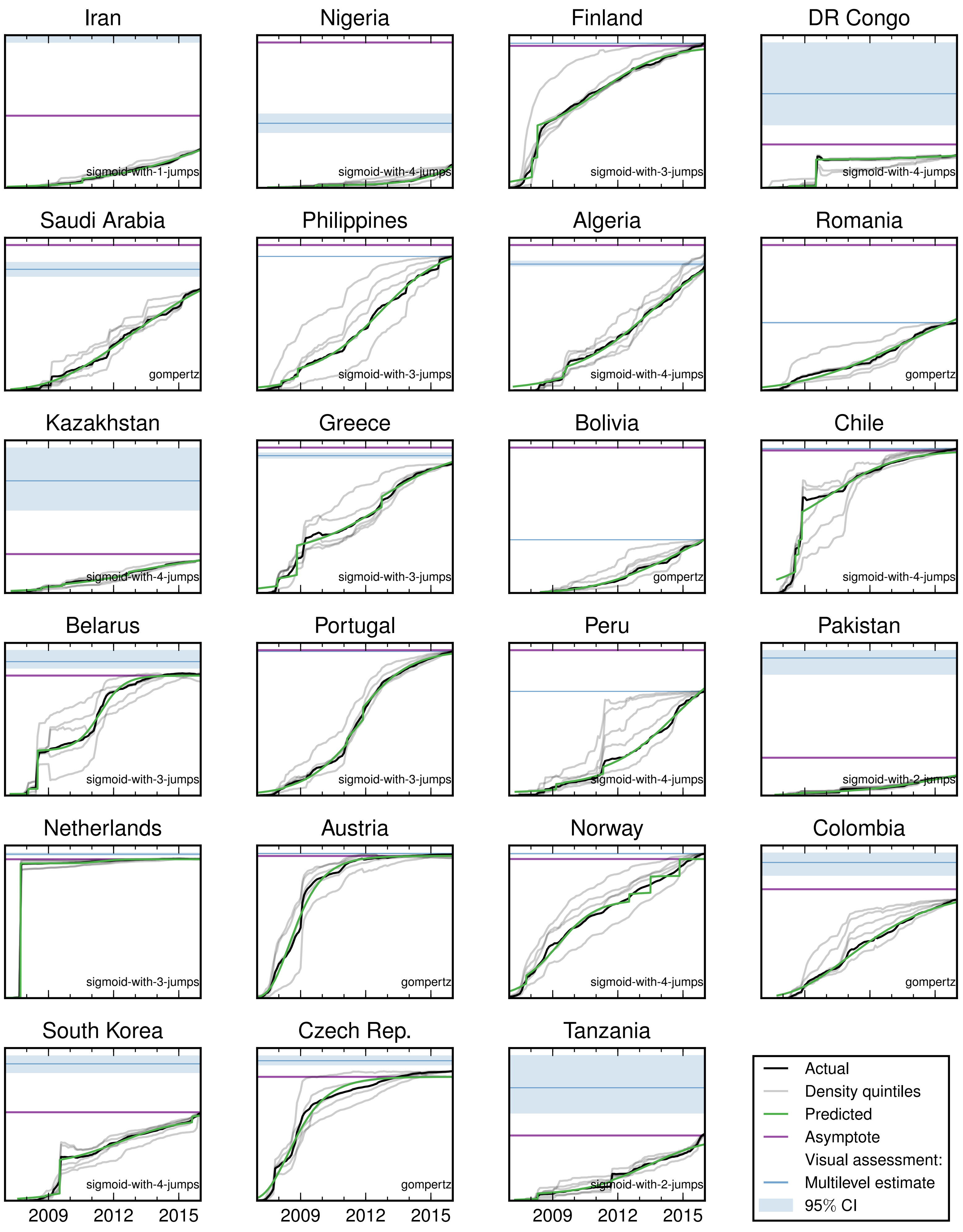

Iran
sigmoid-with-1-jumps
Nigeria
sigmoid-with-4-jumps
Finland
sigmoid-with-3-jumps
DR Congo
sigmoid-with-4-jumps
Saudi Arabia
gompertz
Philippines
sigmoid-with-3-jumps
Algeria
sigmoid-with-4-jumps
Romania
gompertz
Kazakhstan
sigmoid-with-4-jumps
Greece
sigmoid-with-3-jumps
Bolivia
gompertz
Chile
sigmoid-with-4-jumps
Belarus
sigmoid-with-3-jumps
Portugal
sigmoid-with-3-jumps
Peru
sigmoid-with-4-jumps
Pakistan
sigmoid-with-2-jumps
Netherlands
sigmoid-with-3-jumps
Austria
gompertz
Norway
sigmoid-with-4-jumps
Colombia
gompertz
South Korea
sigmoid-with-4-jumps
Czech Rep.
gompertz
Tanzania
sigmoid-with-2-jumps
2009
2012
2015
Actual
Density quintiles
Predicted
Asymptote
Visual assessment:
Multilevel estimate
95% CI

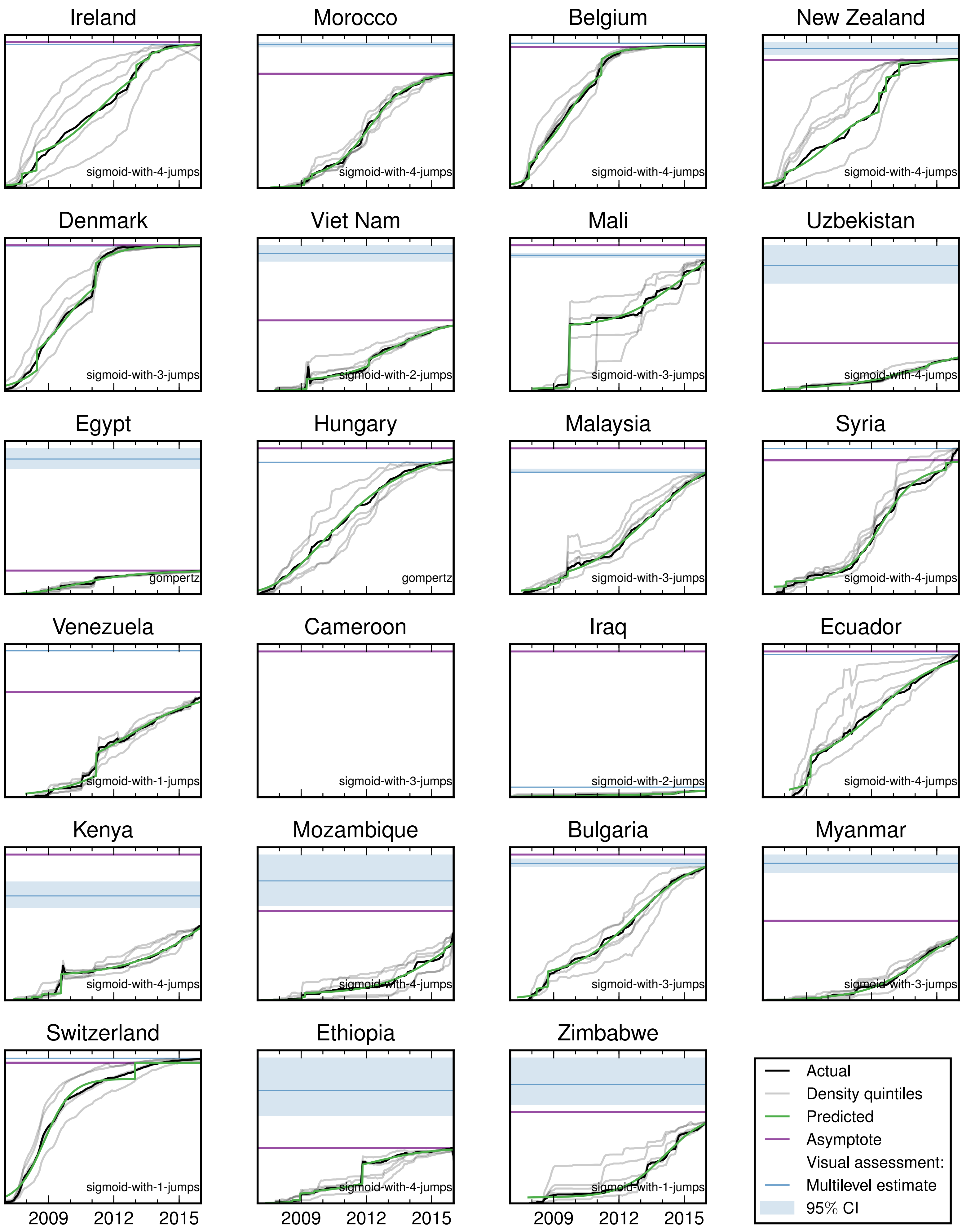

Ireland
sigmoid-with-4-jumps
Morocco
sigmoid-with-4-jumps
Belgium
sigmoid-with-4-jumps
New Zealand
sigmoid-with-4-jumps
Denmark
sigmoid-with-3-jumps
Viet Nam
sigmoid-with-2-jumps
Mali
sigmoid-with-3-jumps
Uzbekistan
sigmoid-with-4-jumps
Egypt
gompertz
Hungary
gompertz
Malaysia
sigmoid-with-3-jumps
Syria
sigmoid-with-4-jumps
Venezuela
sigmoid-with-1-jumps
Cameroon
sigmoid-with-3-jumps
Iraq
sigmoid-with-2-jumps
Ecuador
sigmoid-with-4-jumps
Kenya
sigmoid-with-4-jumps
Mozambique
sigmoid-with-4-jumps
Bulgaria
sigmoid-with-3-jumps
Myanmar
sigmoid-with-3-jumps
Switzerland
sigmoid-with-1-jumps
Ethiopia
sigmoid-with-4-jumps
Zimbabwe
sigmoid-with-1-jumps
2009
2012
2015
Actual
Density quintiles
Predicted
Asymptote
Visual assessment:
Multilevel estimate
95% CI

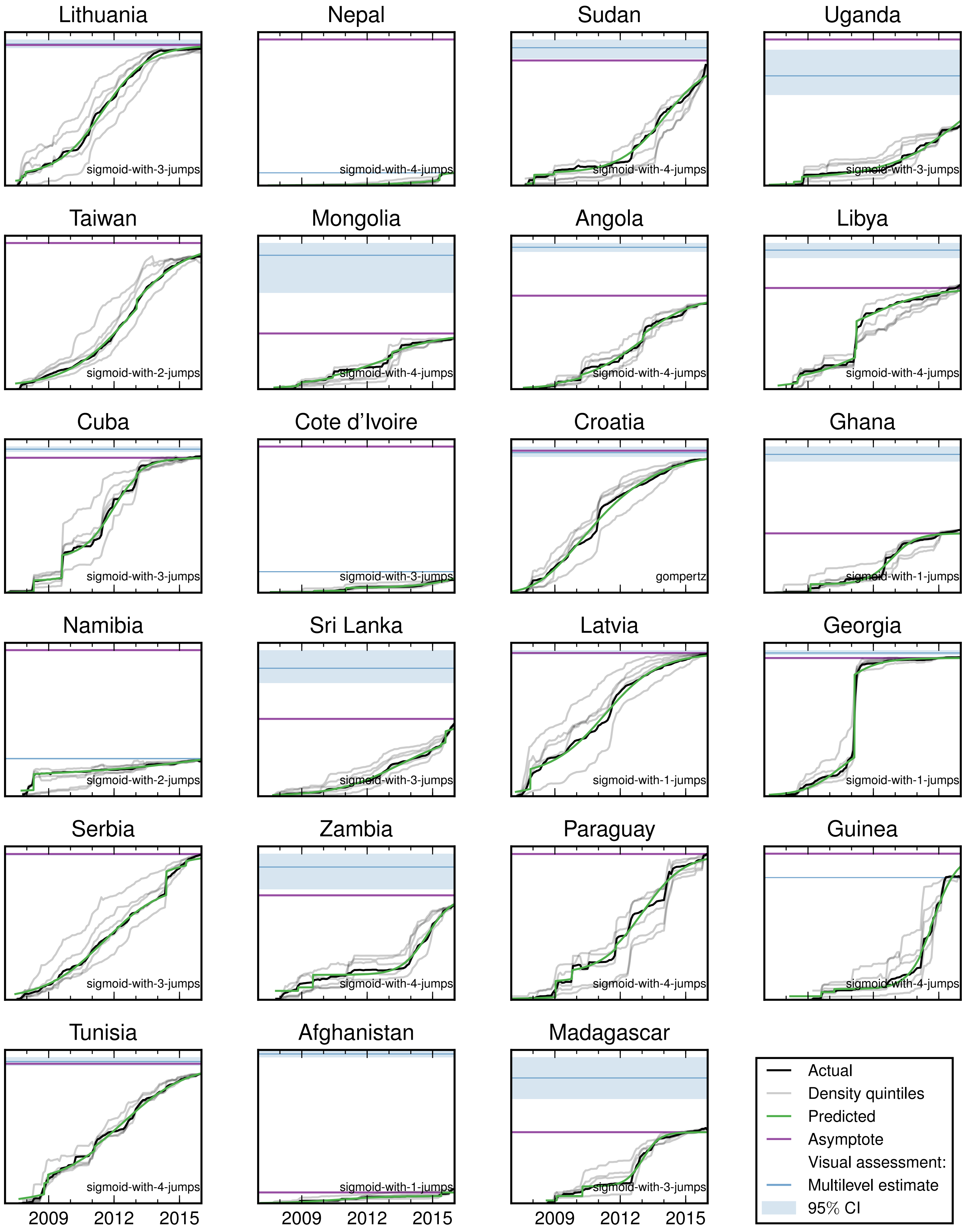

Lithuania
sigmoid-with-3-jumps
Nepal
sigmoid-with-4-jumps
Sudan
sigmoid-with-4-jumps
Uganda
sigmoid-with-3-jumps
Taiwan
sigmoid-with-2-jumps
Mongolia
sigmoid-with-4-jumps
Angola
sigmoid-with-4-jumps
Libya
sigmoid-with-4-jumps
Cuba
sigmoid-with-3-jumps
Cote d'Ivoire
sigmoid-with-3-jumps
Croatia
gompertz
Ghana
sigmoid-with-1-jumps
Namibia
sigmoid-with-2-jumps
Sri Lanka
sigmoid-with-3-jumps
Latvia
sigmoid-with-1-jumps
Georgia
sigmoid-with-1-jumps
Serbia
sigmoid-with-3-jumps
Zambia
sigmoid-with-4-jumps
Paraguay
sigmoid-with-4-jumps
Guinea
sigmoid-with-4-jumps
Tunisia
sigmoid-with-4-jumps
Afghanistan
sigmoid-with-1-jumps
Madagascar
sigmoid-with-3-jumps
2009
2012
2015
Actual
Density quintiles
Predicted
Asymptote
Visual assessment:
Multilevel estimate
95% CI

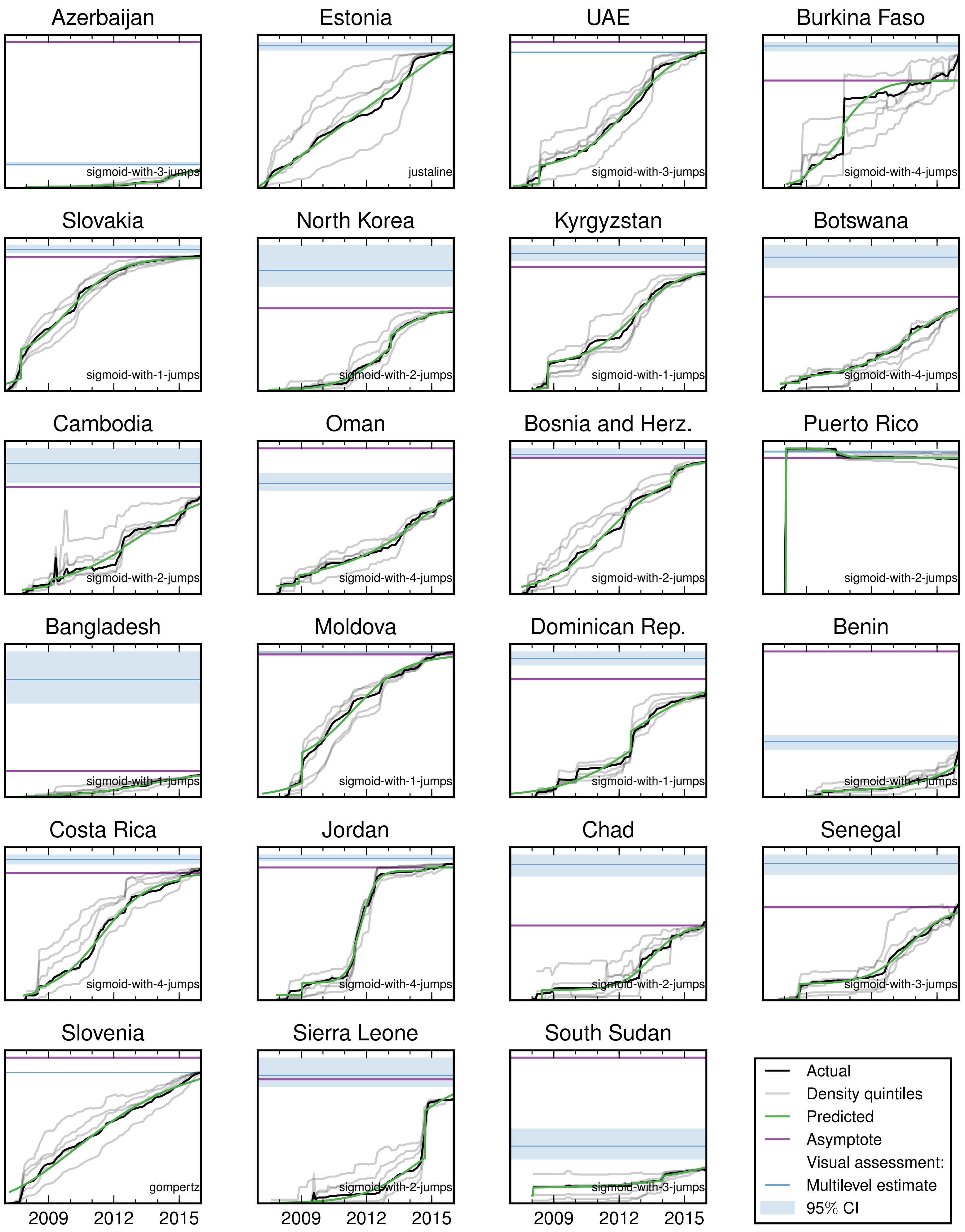
Azerbaijan
sigmoid-with-3-jumps
Estonia
justaline
UAE
sigmoid-with-3-jumps
Burkina Faso
sigmoid-with-4-jumps
Slovakia
sigmoid-with-1-jumps
North Korea
sigmoid-with-2-jumps
Kyrgyzstan
sigmoid-with-1-jumps
Botswana
sigmoid-with-4-jumps
Cambodia
sigmoid-with-2-jumps
Oman
sigmoid-with-4-jumps
Bosnia and Herz.
sigmoid-with-2-jumps
Puerto Rico
sigmoid-with-2-jumps
Bangladesh
sigmoid-with-1-jumps
Moldova
sigmoid-with-1-jumps
Dominican Rep.
sigmoid-with-1-jumps
Benin
sigmoid-with-1-jumps
Costa Rica
sigmoid-with-4-jumps
Jordan
sigmoid-with-4-jumps
Chad
sigmoid-with-2-jumps
Senegal
sigmoid-with-3-jumps
Slovenia
gompertz
Sierra Leone
sigmoid-with-2-jumps
South Sudan
sigmoid-with-3-jumps
2009
2012
2015
Actual
Density quintiles
Predicted
Asymptote
Visual assessment:
Multilevel estimate
95% CI

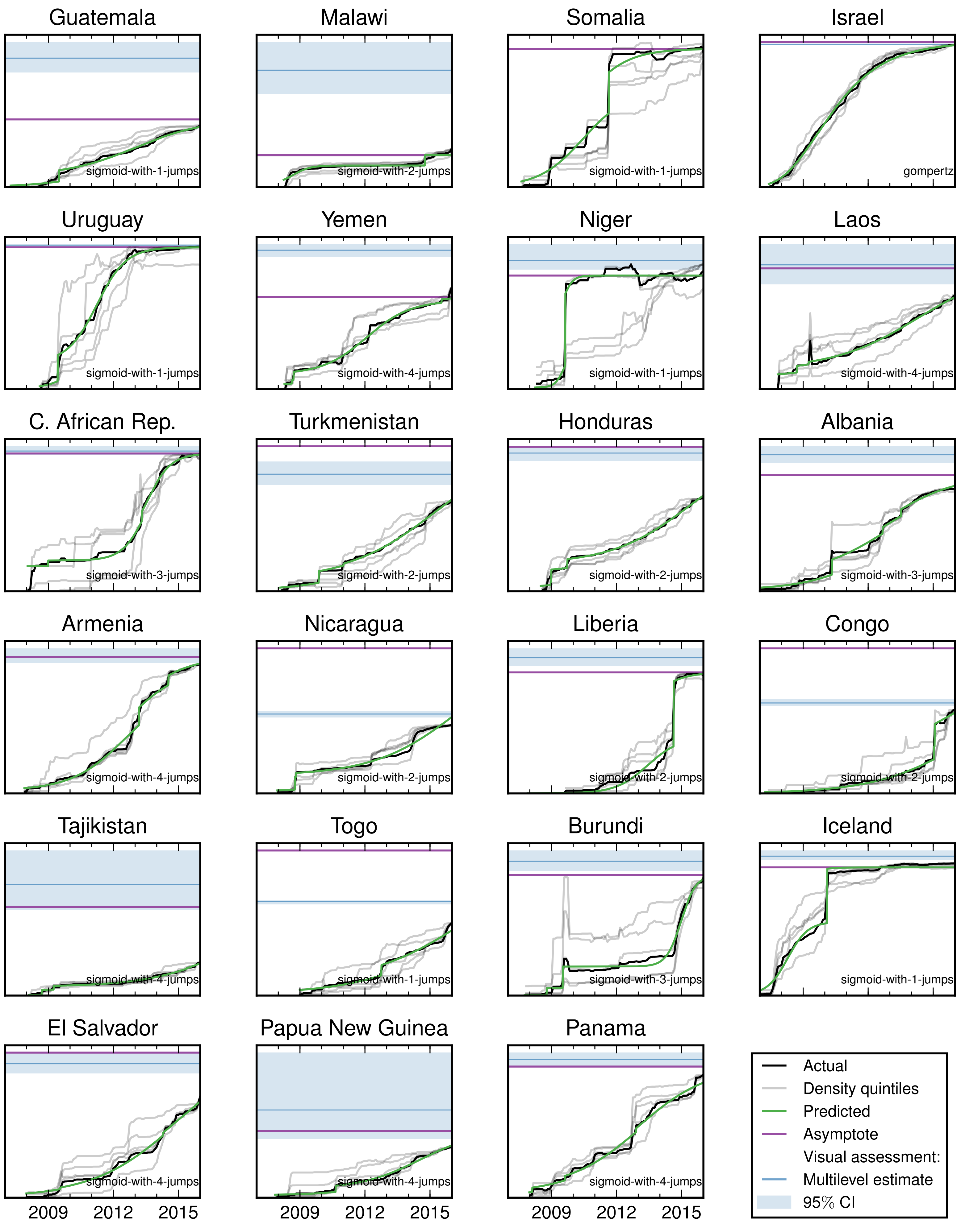

Guatemala
sigmoid-with-1-jumps
Malawi
sigmoid-with-2-jumps
Somalia
sigmoid-with-1-jumps
Israel
gompertz
Uruguay
sigmoid-with-1-jumps
Yemen
sigmoid-with-4-jumps
Niger
sigmoid-with-1-jumps
Laos
sigmoid-with-4-jumps
C. African Rep.
sigmoid-with-3-jumps
Turkmenistan
sigmoid-with-2-jumps
Honduras
sigmoid-with-2-jumps
Albania
sigmoid-with-3-jumps
Armenia
sigmoid-with-4-jumps
Nicaragua
sigmoid-with-2-jumps
Liberia
sigmoid-with-2-jumps
Congo
sigmoid-with-2-jumps
Tajikistan
sigmoid-with-4-jumps
Togo
sigmoid-with-1-jumps
Burundi
sigmoid-with-3-jumps
Iceland
sigmoid-with-1-jumps
El Salvador
sigmoid-with-4-jumps
Papua New Guinea
sigmoid-with-4-jumps
Panama
sigmoid-with-4-jumps
2009
2012
2015
Actual
Density quintiles
Predicted
Asymptote
Visual assessment:
Multilevel estimate
95% CI

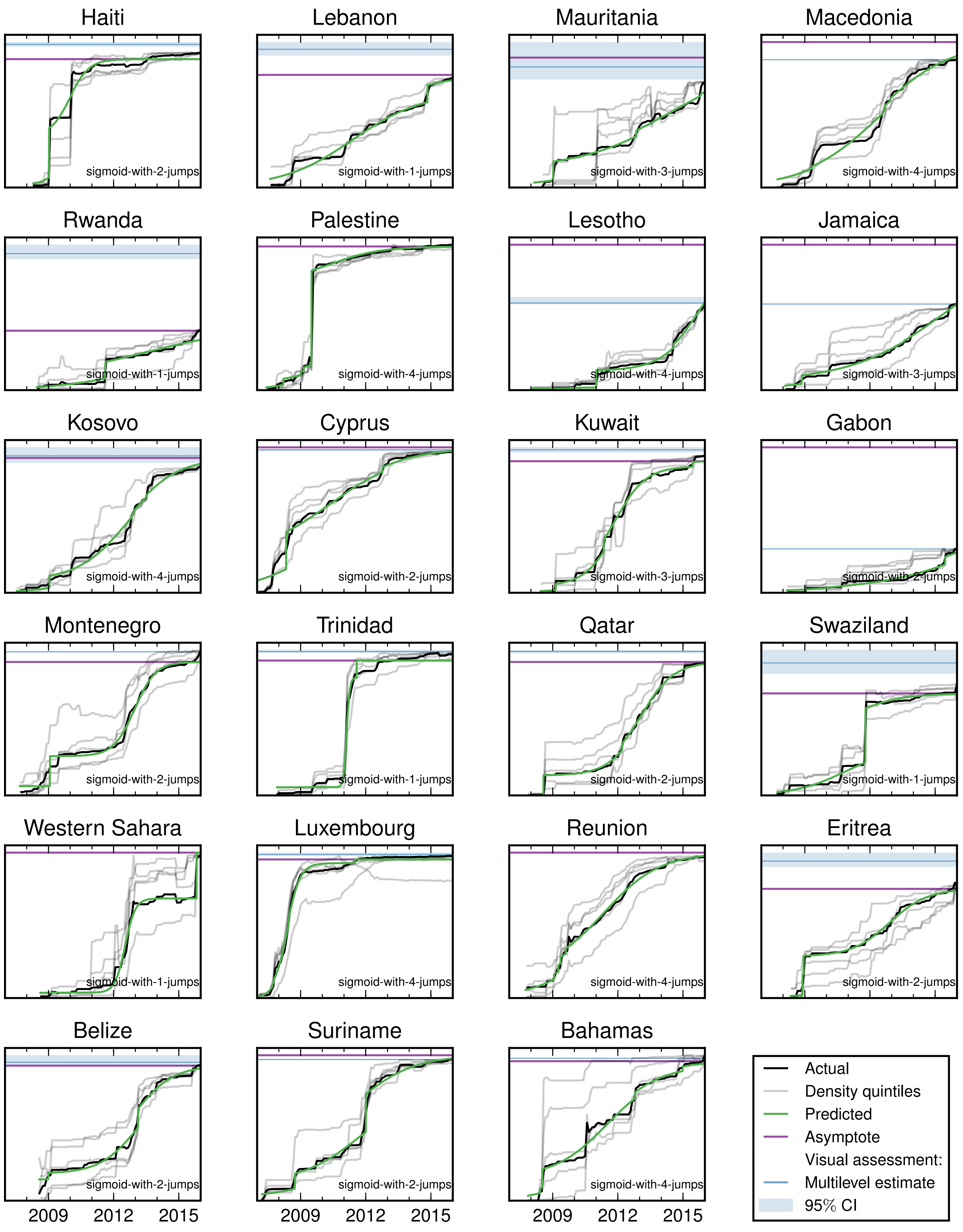
Haiti
sigmoid-with-2-jumps
Lebanon
sigmoid-with-1-jumps
Mauritania
sigmoid-with-3-jumps
Macedonia
sigmoid-with-4-jumps
Rwanda
sigmoid-with-1-jumps
Palestine
sigmoid-with-4-jumps
Lesotho
sigmoid-with-4-jumps
Jamaica
sigmoid-with-3-jumps
Kosovo
sigmoid-with-4-jumps
Cyprus
sigmoid-with-2-jumps
Kuwait
sigmoid-with-3-jumps
Gabon
sigmoid-with-2-jumps
Montenegro
sigmoid-with-2-jumps
Trinidad
sigmoid-with-1-jumps
Qatar
sigmoid-with-2-jumps
Swaziland
sigmoid-with-1-jumps
Western Sahara
sigmoid-with-1-jumps
Luxembourg
sigmoid-with-4-jumps
Reunion
sigmoid-with-4-jumps
Eritrea
sigmoid-with-2-jumps
Belize
sigmoid-with-2-jumps
Suriname
sigmoid-with-2-jumps
Bahamas
sigmoid-with-4-jumps
2009
2012
2015
Actual
Density quintiles
Predicted
Asymptote
Visual assessment:
Multilevel estimate
95% CI

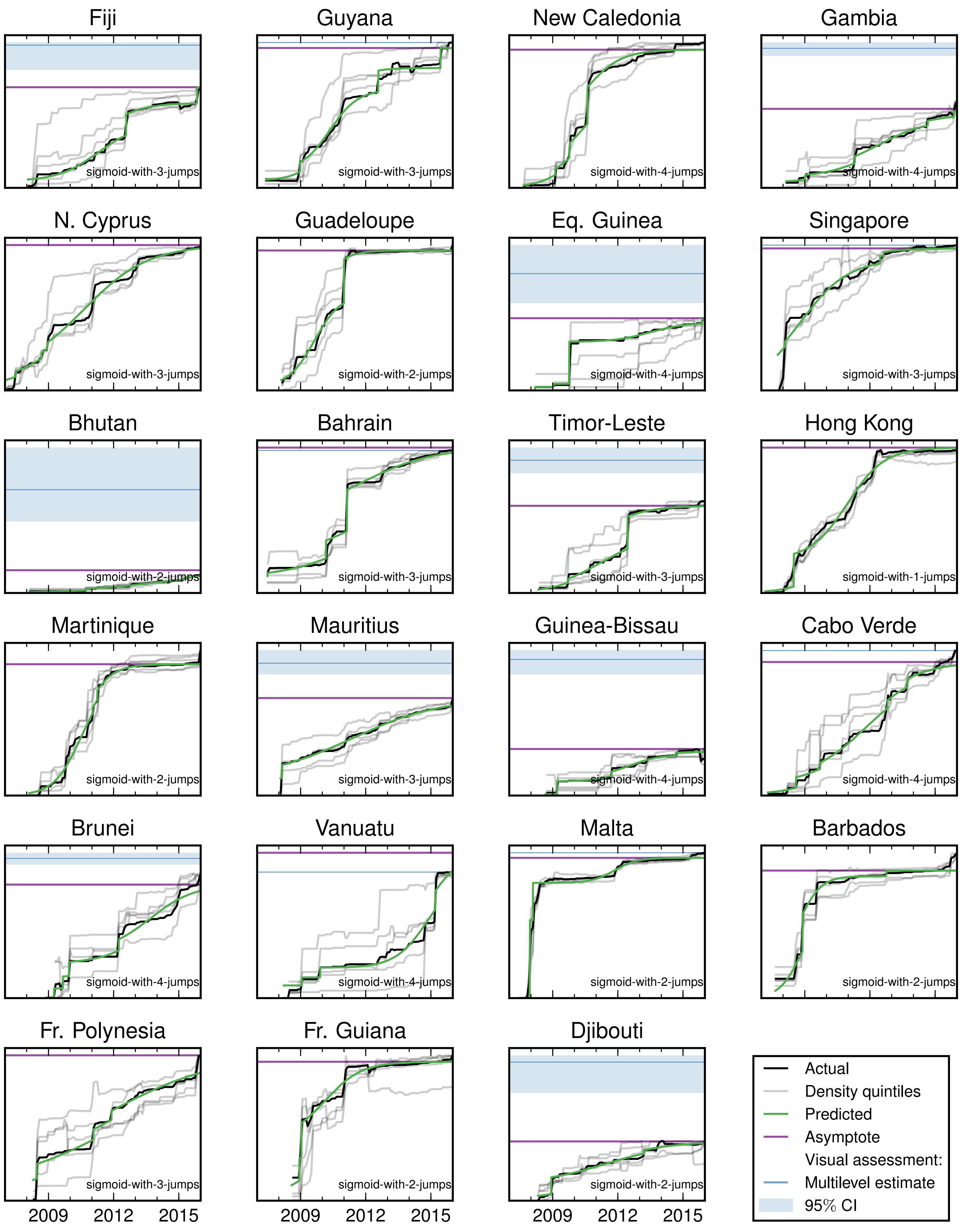
Fiji
sigmoid-with-3-jumps
Guyana
sigmoid-with-3-jumps
New Caledonia
sigmoid-with-4-jumps
Gambia
sigmoid-with-4-jumps
N. Cyprus
sigmoid-with-3-jumps
Guadeloupe
sigmoid-with-2-jumps
Eq. Guinea
sigmoid-with-4-jumps
Singapore
sigmoid-with-3-jumps
Bhutan
sigmoid-with-2-jumps
Bahrain
sigmoid-with-3-jumps
Timor-Leste
sigmoid-with-3-jumps
Hong Kong
sigmoid-with-1-jumps
Martinique
sigmoid-with-2-jumps
Mauritius
sigmoid-with-3-jumps
Guinea-Bissau
sigmoid-with-4-jumps
Cabo Verde
sigmoid-with-4-jumps
Brunei
sigmoid-with-4-jumps
Vanuatu
sigmoid-with-4-jumps
Malta
sigmoid-with-2-jumps
Barbados
sigmoid-with-2-jumps
Fr. Polynesia
sigmoid-with-3-jumps
Fr. Guiana
sigmoid-with-2-jumps
Djibouti
sigmoid-with-2-jumps
2009
2012
2015
Actual
Density quintiles
Predicted
Asymptote
Visual assessment:
Multilevel estimate
95% CI

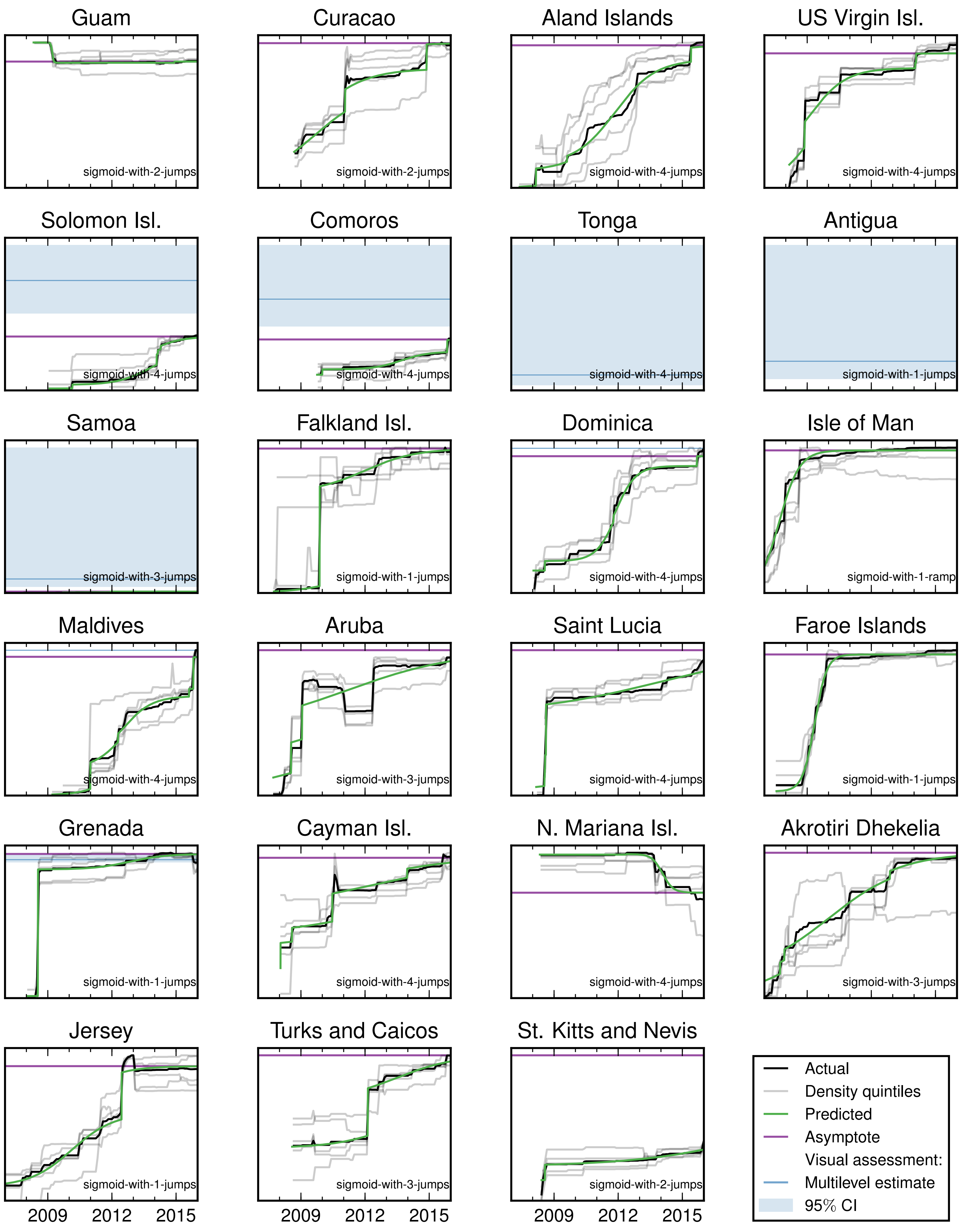

Guam
sigmoid-with-2-jumps
Curacao
sigmoid-with-2-jumps
Aland Islands
sigmoid-with-4-jumps
US Virgin Isl.
sigmoid-with-4-jumps
Solomon Isl.
sigmoid-with-4-jumps
Comoros
sigmoid-with-4-jumps
Tonga
sigmoid-with-4-jumps
Antigua
sigmoid-with-1-jumps
Samoa
sigmoid-with-3-jumps
Falkland Isl.
sigmoid-with-1-jumps
Dominica
sigmoid-with-4-jumps
Isle of Man
sigmoid-with-1-ramp
Maldives
sigmoid-with-4-jumps
Aruba
sigmoid-with-3-jumps
Saint Lucia
sigmoid-with-4-jumps
Faroe Islands
sigmoid-with-1-jumps
Grenada
sigmoid-with-1-jumps
Cayman Isl.
sigmoid-with-4-jumps
N. Mariana Isl.
sigmoid-with-4-jumps
Akrotiri Dhekelia
sigmoid-with-3-jumps
Jersey
sigmoid-with-1-jumps
Turks and Caicos
sigmoid-with-3-jumps
St. Kitts and Nevis
sigmoid-with-2-jumps
2009
2012
2015
Actual
Density quintiles
Predicted
Asymptote
Visual assessment:
Multilevel estimate
95% CI

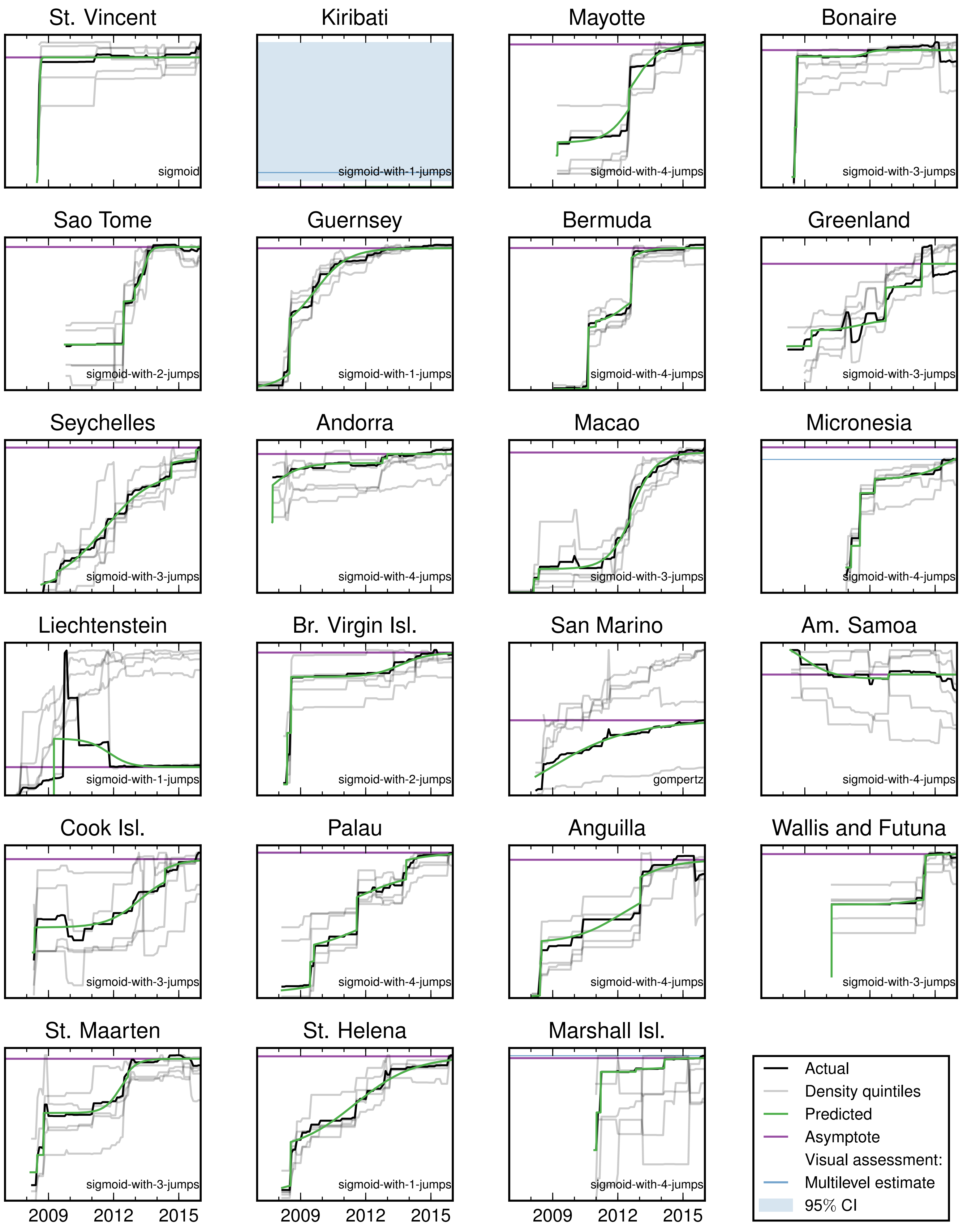
St. Vincent
sigmoid
Kiribati
sigmoid-with-1-jumps
Mayotte
sigmoid-with-4-jumps
Bonaire
sigmoid-with-3-jumps
Sao Tome
sigmoid-with-2-jumps
Guernsey
sigmoid-with-1-jumps
Bermuda
sigmoid-with-4-jumps
Greenland
sigmoid-with-3-jumps
Seychelles
sigmoid-with-3-jumps
Andorra
sigmoid-with-4-jumps
Macao
sigmoid-with-3-jumps
Micronesia
sigmoid-with-4-jumps
Liechtenstein
sigmoid-with-1-jumps
Br. Virgin Isl.
sigmoid-with-2-jumps
San Marino
gompertz
Am. Samoa
sigmoid-with-4-jumps
Cook Isl.
sigmoid-with-3-jumps
Palau
sigmoid-with-4-jumps
Anguilla
sigmoid-with-4-jumps
Wallis and Futuna
sigmoid-with-3-jumps
St. Maarten
sigmoid-with-3-jumps
St. Helena
sigmoid-with-1-jumps
Marshall Isl.
sigmoid-with-4-jumps
2009
2012
2015
Actual
Density quintiles
Predicted
Asymptote
Visual assessment:
Multilevel estimate
95% CI

F Parametric fits by decile and world region

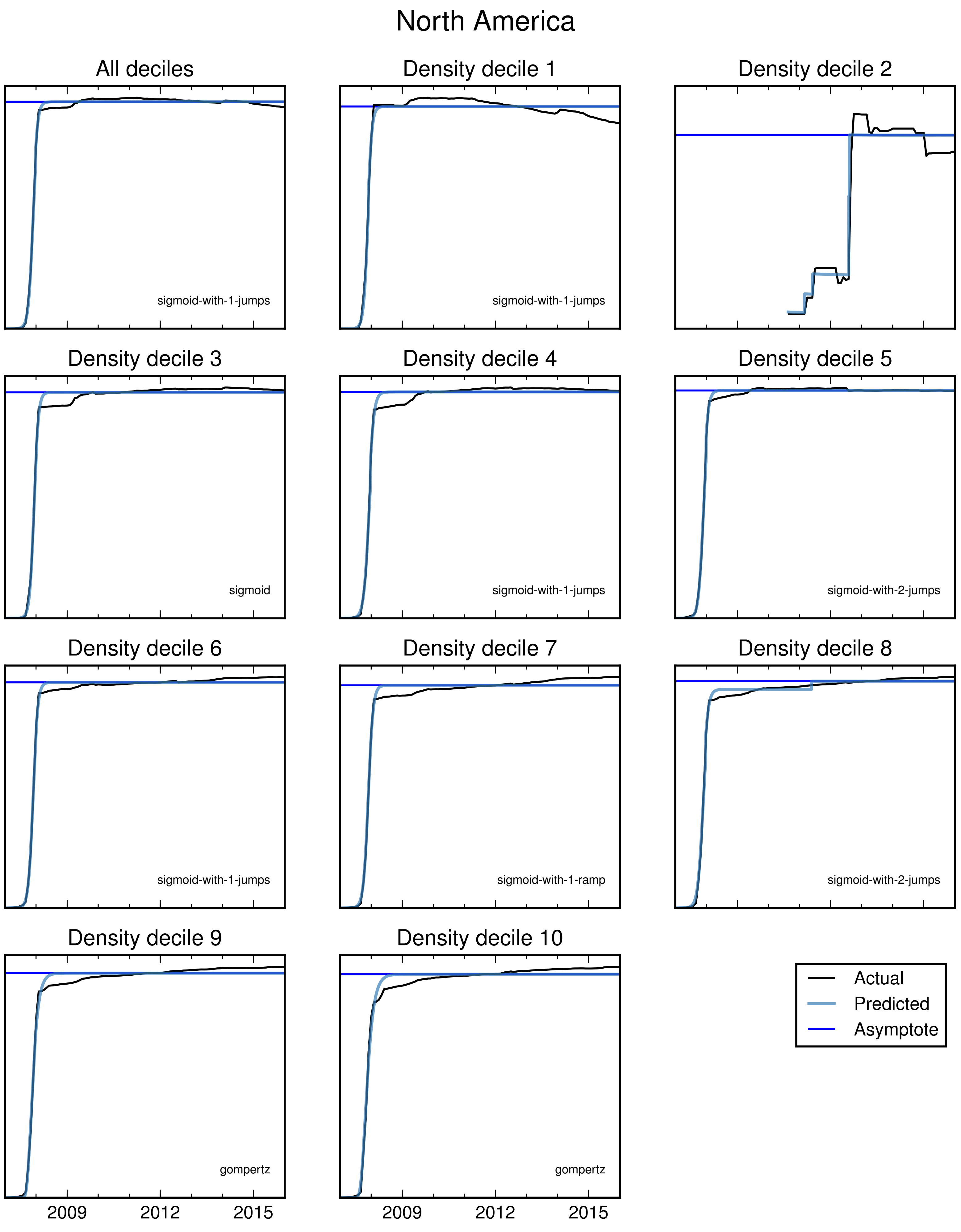
North America
All deciles
sigmoid-with-1-jumps
Density decile 1
sigmoid-with-1-jumps
Density decile 2
Density decile 3
sigmoid
Density decile 4
sigmoid-with-1-jumps
Density decile 5
sigmoid-with-2-jumps
Density decile 6
sigmoid-with-1-jumps
Density decile 7
sigmoid-with-1-ramp
Density decile 8
sigmoid-with-2-jumps
Density decile 9
gompertz
Density decile 10
gompertz
2009
2012
2015
Actual
Predicted
Asymptote

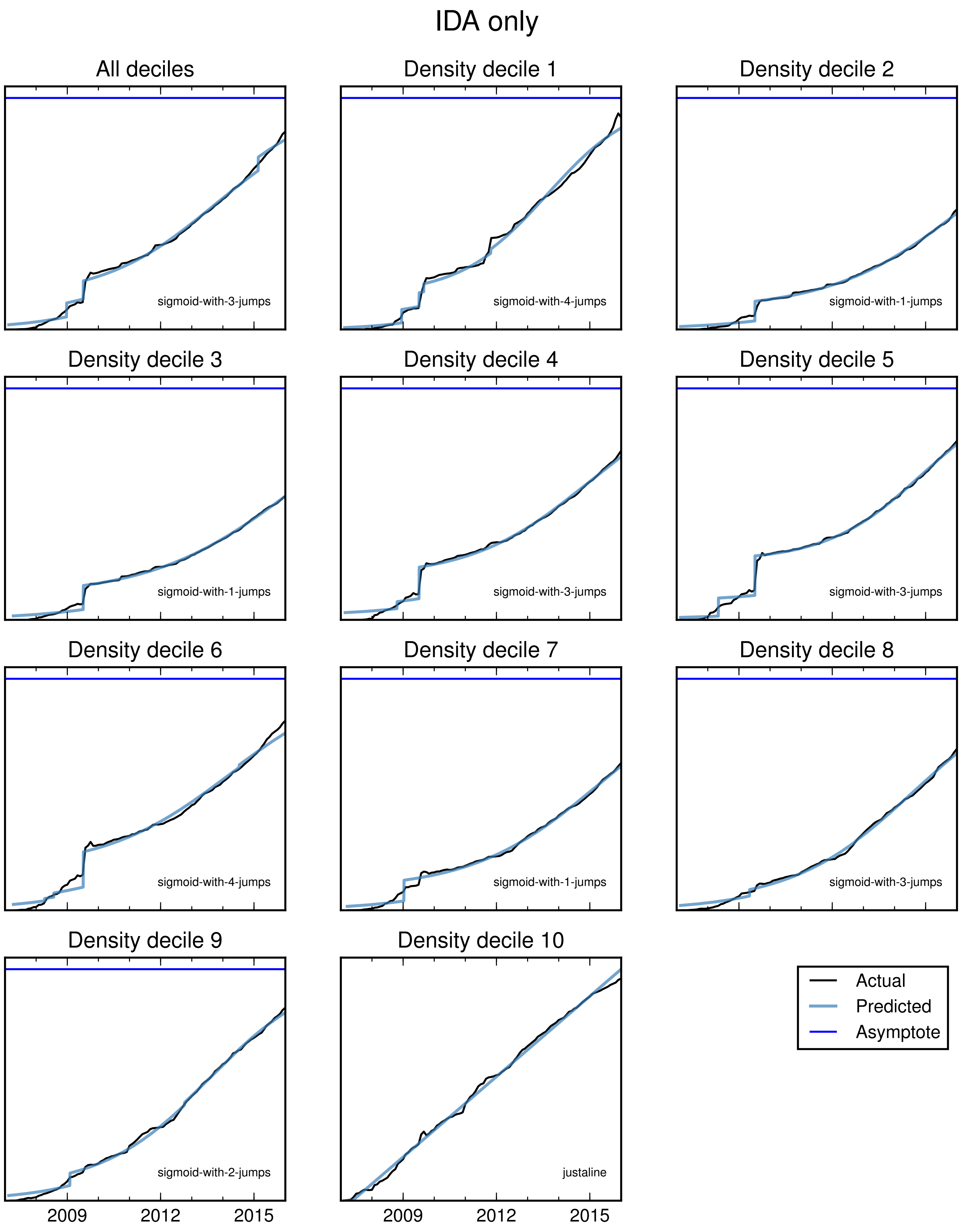
IDA only
All deciles
sigmoid-with-3-jumps
Density decile 1
sigmoid-with-4-jumps
Density decile 2
sigmoid-with-1-jumps
Density decile 3
sigmoid-with-1-jumps
Density decile 4
sigmoid-with-3-jumps
Density decile 5
sigmoid-with-3-jumps
Density decile 6
sigmoid-with-4-jumps
Density decile 7
sigmoid-with-1-jumps
Density decile 8
sigmoid-with-3-jumps
Density decile 9
sigmoid-with-2-jumps
Density decile 10
justaline
2009
2012
2015
Actual
Predicted
Asymptote

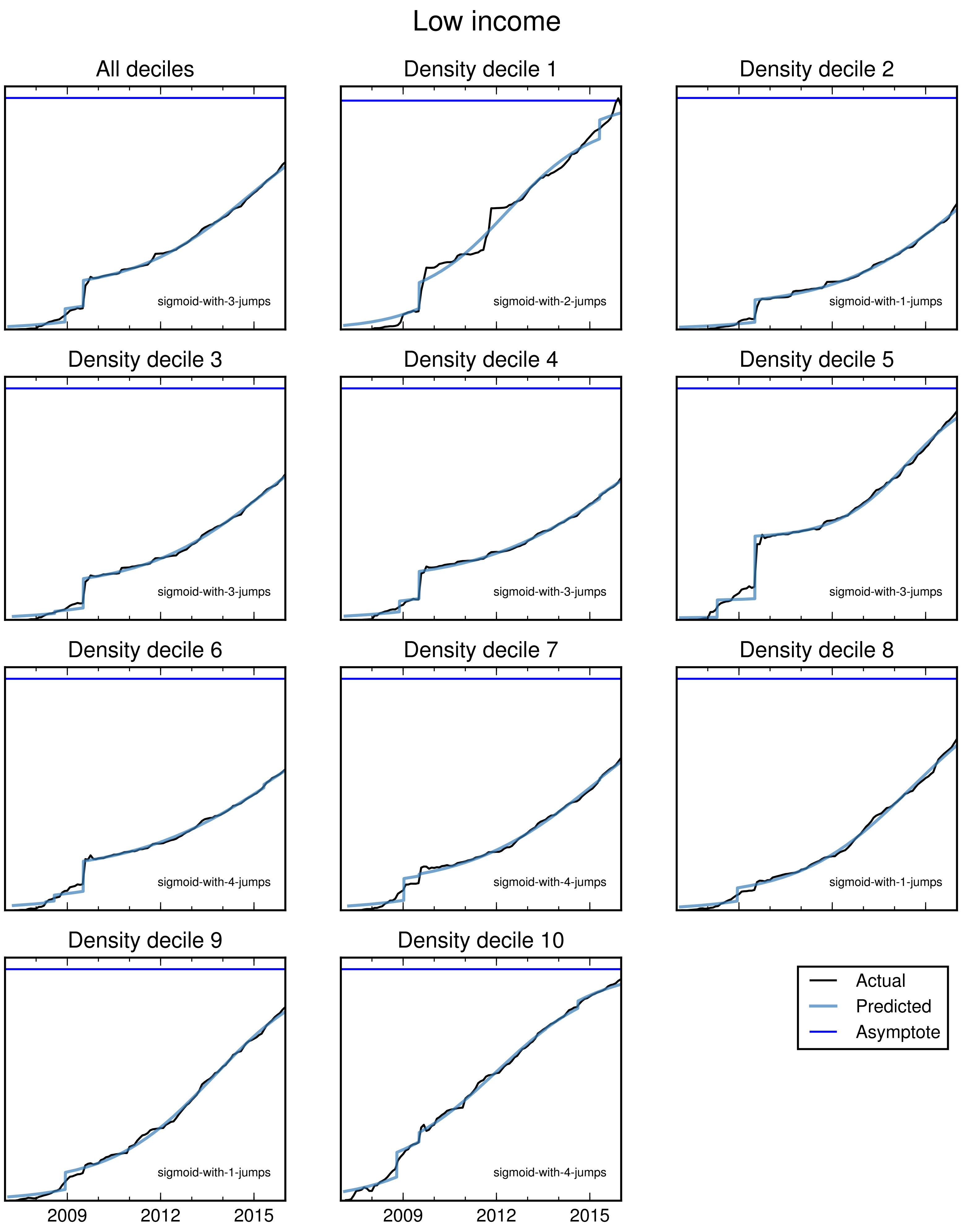
Low income
All deciles
sigmoid-with-3-jumps
Density decile 1
sigmoid-with-2-jumps
Density decile 2
sigmoid-with-1-jumps
Density decile 3
sigmoid-with-3-jumps
Density decile 4
sigmoid-with-3-jumps
Density decile 5
sigmoid-with-3-jumps
Density decile 6
sigmoid-with-4-jumps
Density decile 7
sigmoid-with-4-jumps
Density decile 8
sigmoid-with-1-jumps
Density decile 9
sigmoid-with-1-jumps
Density decile 10
sigmoid-with-4-jumps
2009
2012
2015
Actual
Predicted
Asymptote

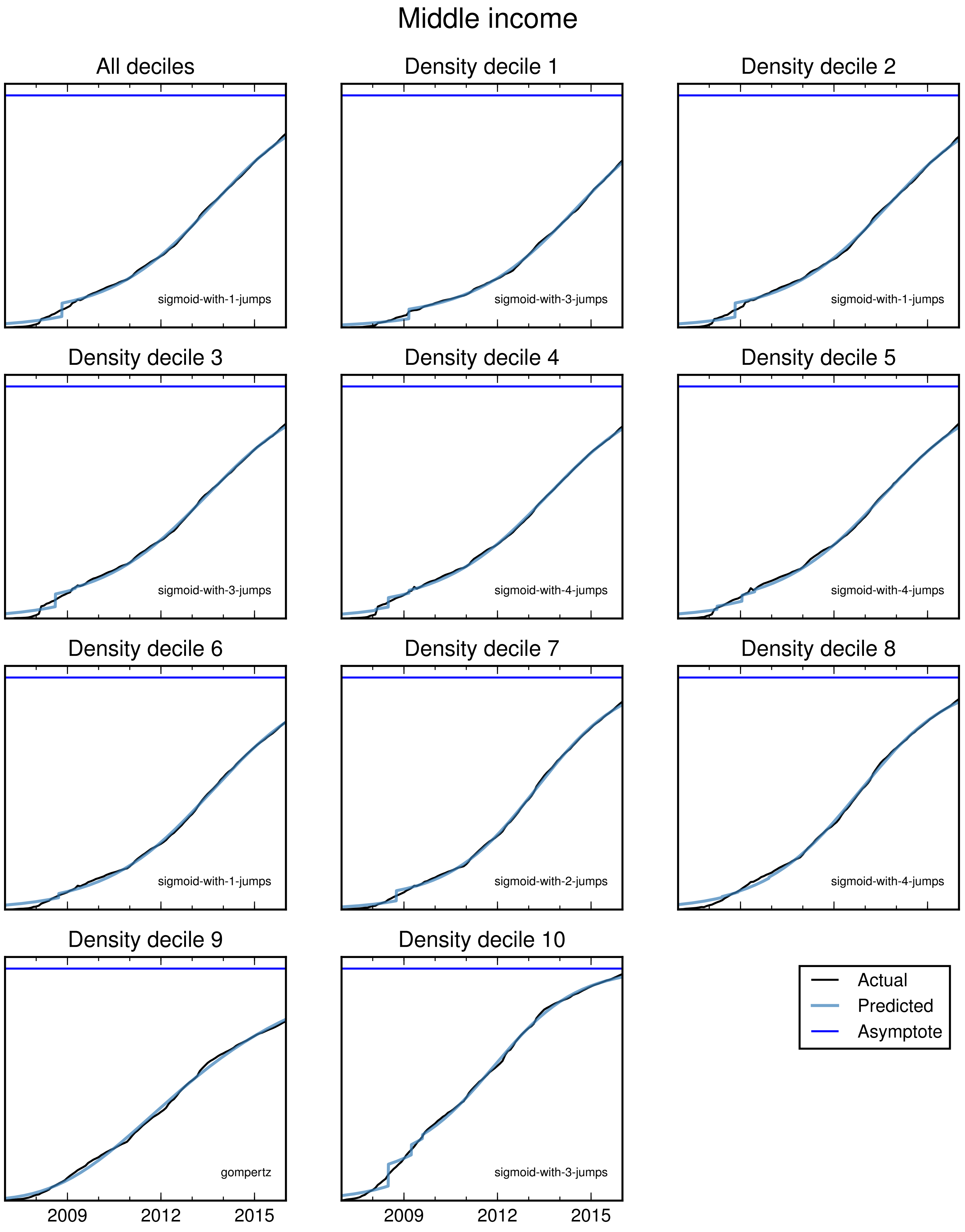

Middle income
All deciles
sigmoid-with-1-jumps
Density decile 1
sigmoid-with-3-jumps
Density decile 2
sigmoid-with-1-jumps
Density decile 3
sigmoid-with-3-jumps
Density decile 4
sigmoid-with-4-jumps
Density decile 5
sigmoid-with-4-jumps
Density decile 6
sigmoid-with-1-jumps
Density decile 7
sigmoid-with-2-jumps
Density decile 8
sigmoid-with-4-jumps
Density decile 9
gompertz
Density decile 10
sigmoid-with-3-jumps
2009
2012
2015
Actual
Predicted
Asymptote

IBRD only

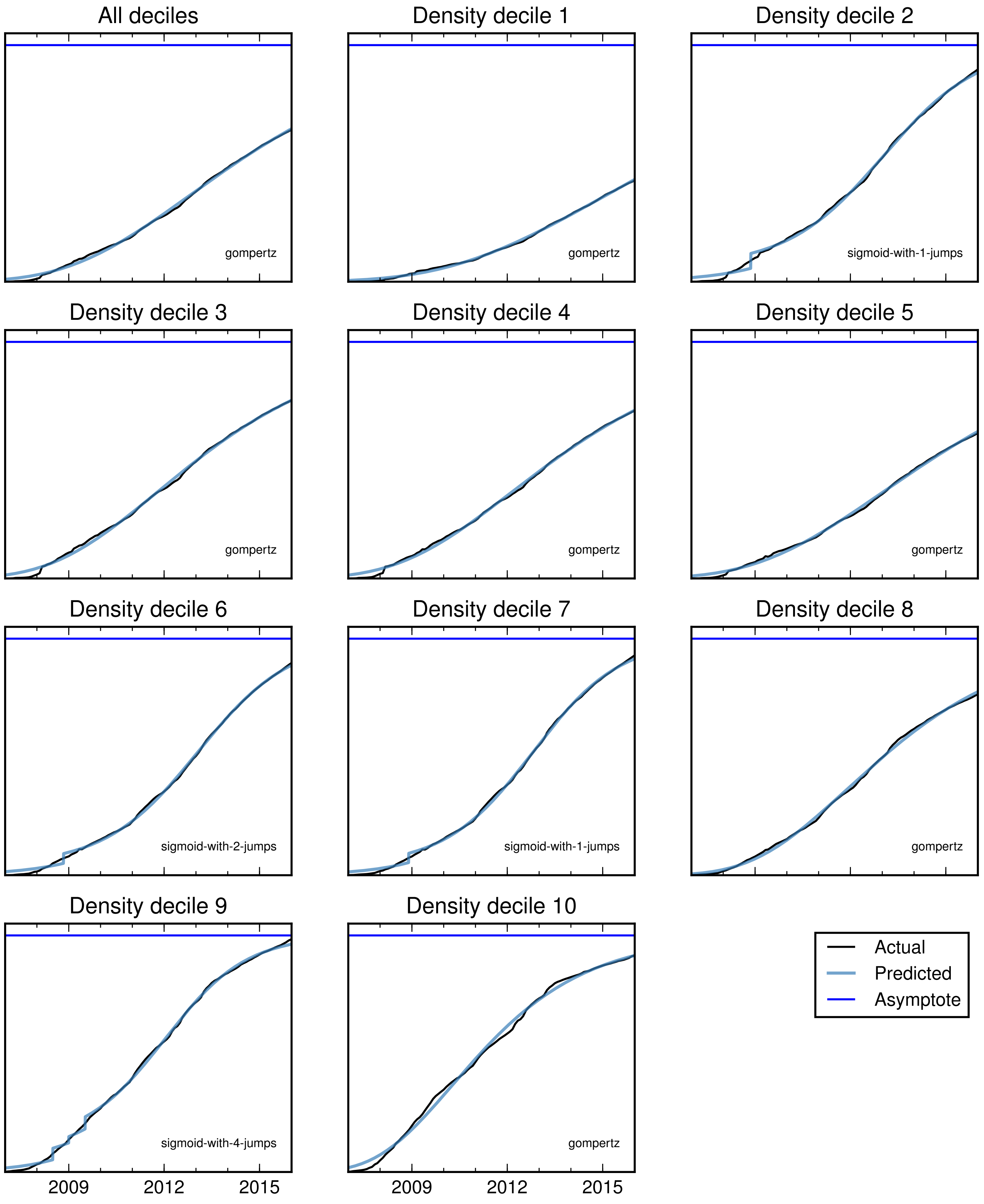

All deciles
Density decile 1
Density decile 2
Density decile 3
Density decile 4
Density decile 5
Density decile 6
Density decile 7
Density decile 8
Density decile 9
Density decile 10
gompertz
sigmoid-with-1-jumps
sigmoid-with-2-jumps
sigmoid-with-4-jumps
2009
2012
2015
Actual
Predicted
Asymptote

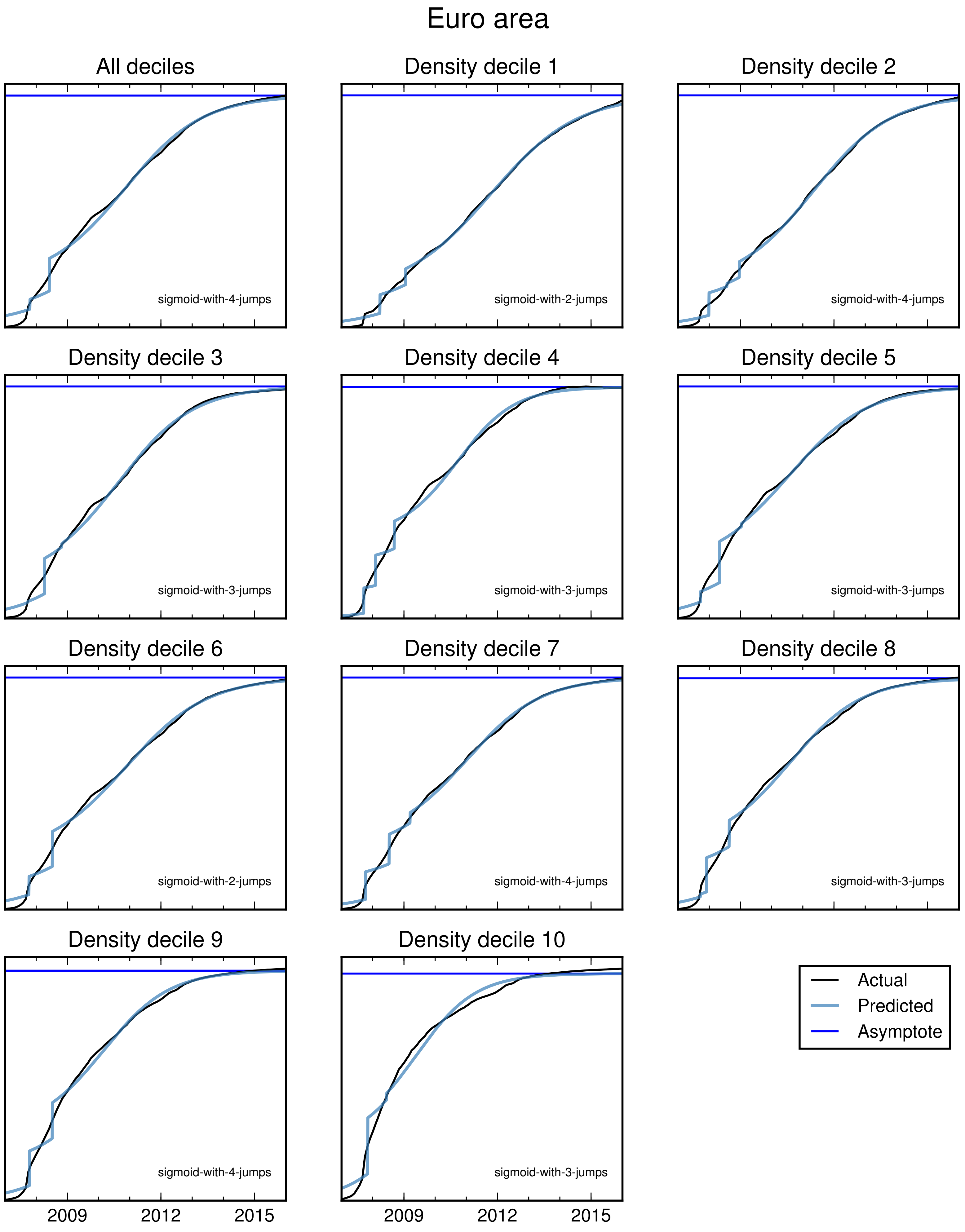
Euro area
All deciles
sigmoid-with-4-jumps
Density decile 1
sigmoid-with-2-jumps
Density decile 2
sigmoid-with-4-jumps
Density decile 3
sigmoid-with-3-jumps
Density decile 4
sigmoid-with-3-jumps
Density decile 5
sigmoid-with-3-jumps
Density decile 6
sigmoid-with-2-jumps
Density decile 7
sigmoid-with-4-jumps
Density decile 8
sigmoid-with-3-jumps
Density decile 9
sigmoid-with-4-jumps
Density decile 10
sigmoid-with-3-jumps
2009
2012
2015
Actual
Predicted
Asymptote

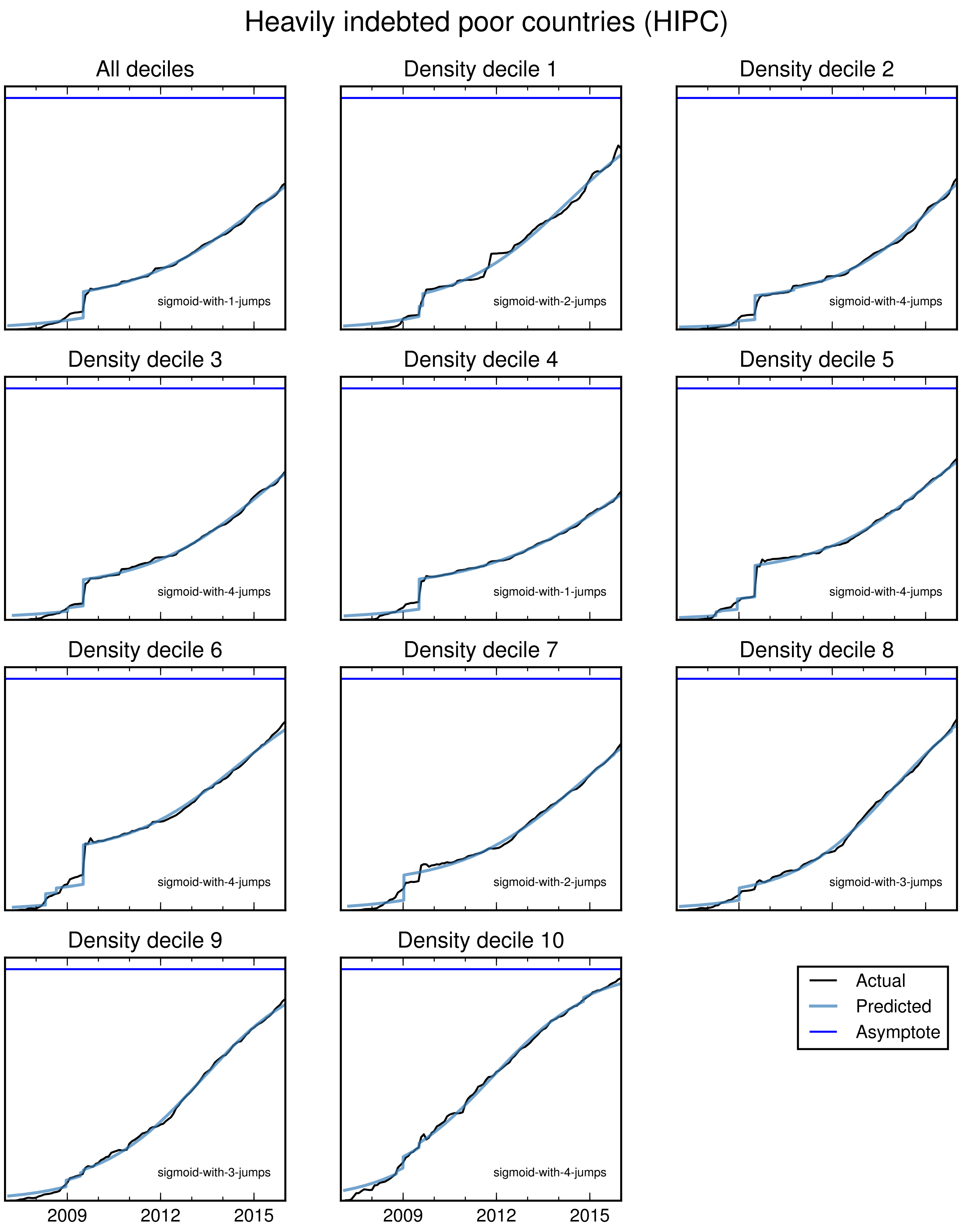

Heavily indebted poor countries (HIPC)
All deciles
sigmoid-with-1-jumps
Density decile 1
sigmoid-with-2-jumps
Density decile 2
sigmoid-with-4-jumps
Density decile 3
sigmoid-with-4-jumps
Density decile 4
sigmoid-with-1-jumps
Density decile 5
sigmoid-with-4-jumps
Density decile 6
sigmoid-with-4-jumps
Density decile 7
sigmoid-with-2-jumps
Density decile 8
sigmoid-with-3-jumps
Density decile 9
sigmoid-with-3-jumps
Density decile 10
sigmoid-with-4-jumps
2009
2012
2015
Actual
Predicted
Asymptote

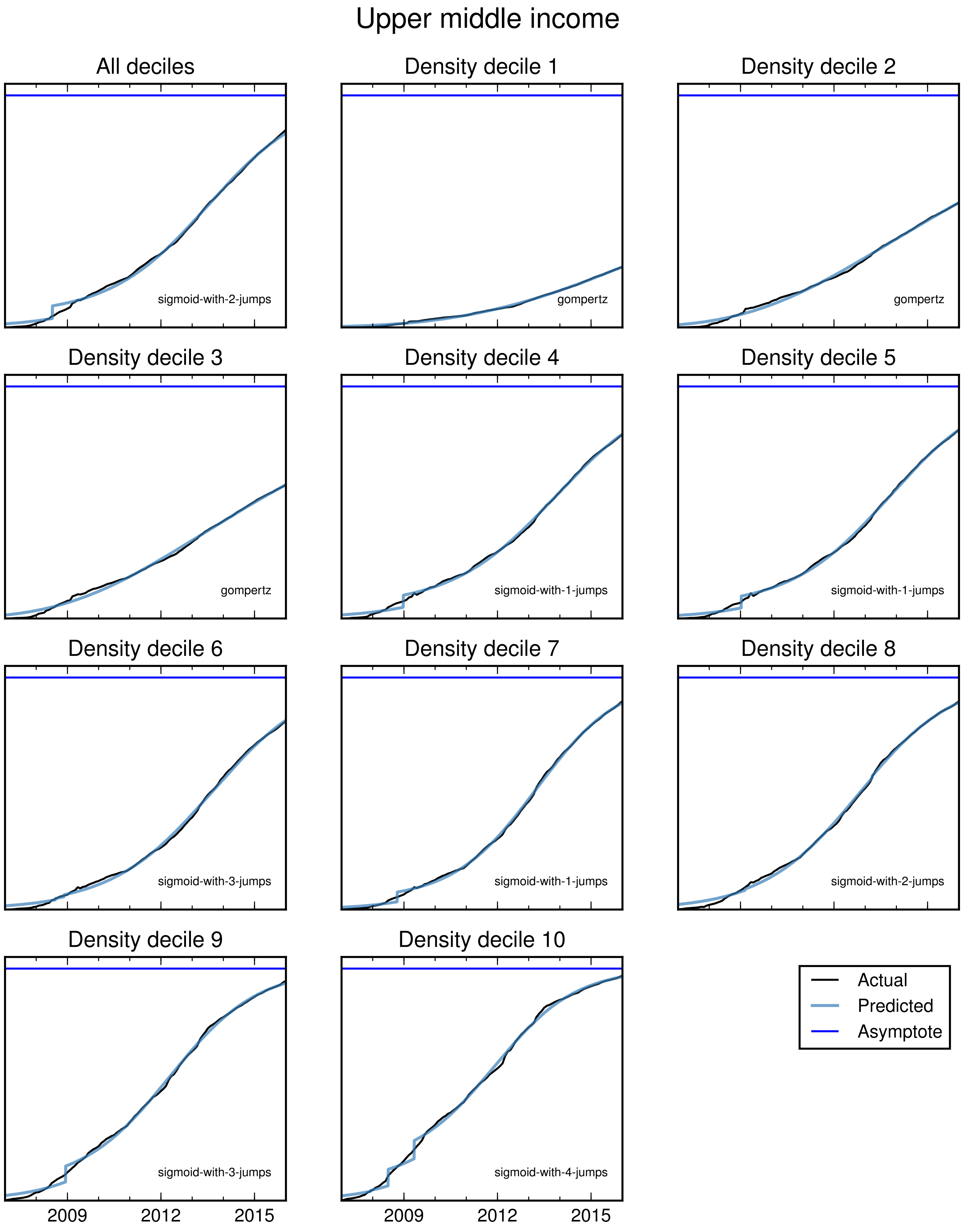

Upper middle income
All deciles
sigmoid-with-2-jumps
Density decile 1
gompertz
Density decile 2
gompertz
Density decile 3
gompertz
Density decile 4
sigmoid-with-1-jumps
Density decile 5
sigmoid-with-1-jumps
Density decile 6
sigmoid-with-3-jumps
Density decile 7
sigmoid-with-1-jumps
Density decile 8
sigmoid-with-2-jumps
Density decile 9
sigmoid-with-3-jumps
Density decile 10
sigmoid-with-4-jumps
2009
2012
2015
2009
2012
2015
Actual
Predicted
Asymptote

High income: nonOECD

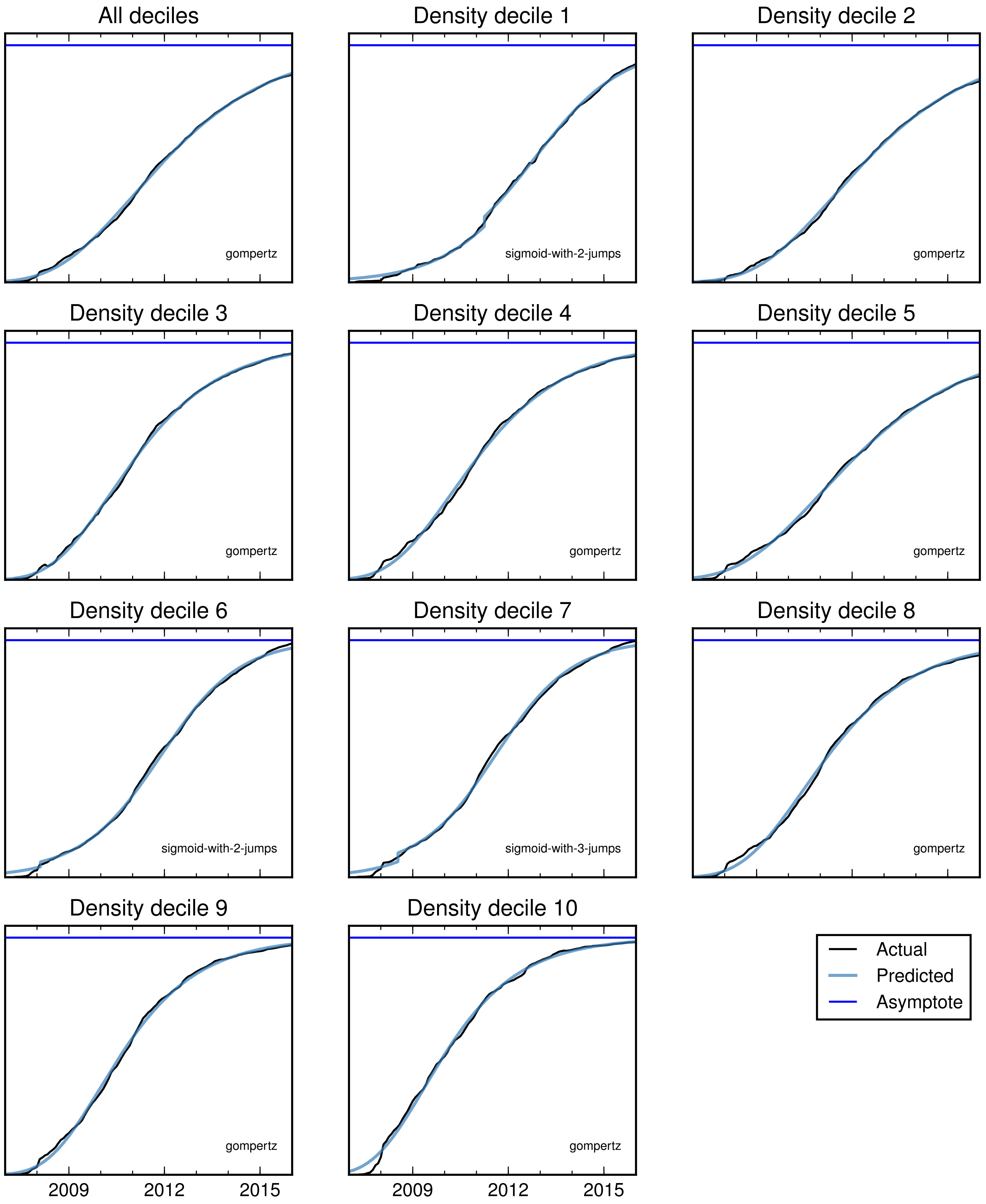

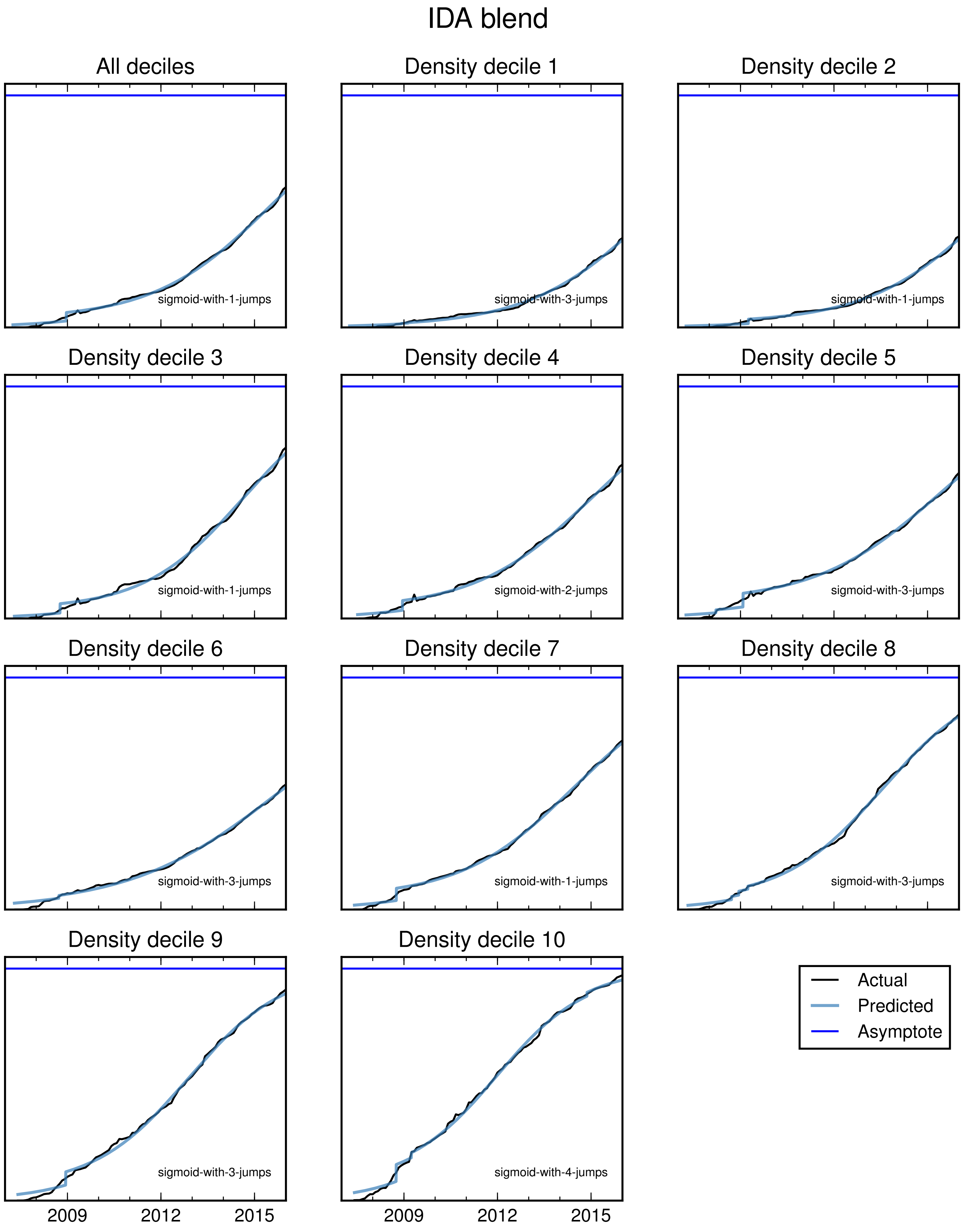

IDA blend
All deciles
Density decile 1
Density decile 2
Density decile 3
Density decile 4
Density decile 5
Density decile 6
Density decile 7
Density decile 8
Density decile 9
Density decile 10
sigmoid-with-1-jumps
sigmoid-with-3-jumps
sigmoid-with-1-jumps
sigmoid-with-1-jumps
sigmoid-with-2-jumps
sigmoid-with-3-jumps
sigmoid-with-3-jumps
sigmoid-with-1-jumps
sigmoid-with-3-jumps
sigmoid-with-3-jumps
sigmoid-with-4-jumps
2009
2012
2015
Actual
Predicted
Asymptote

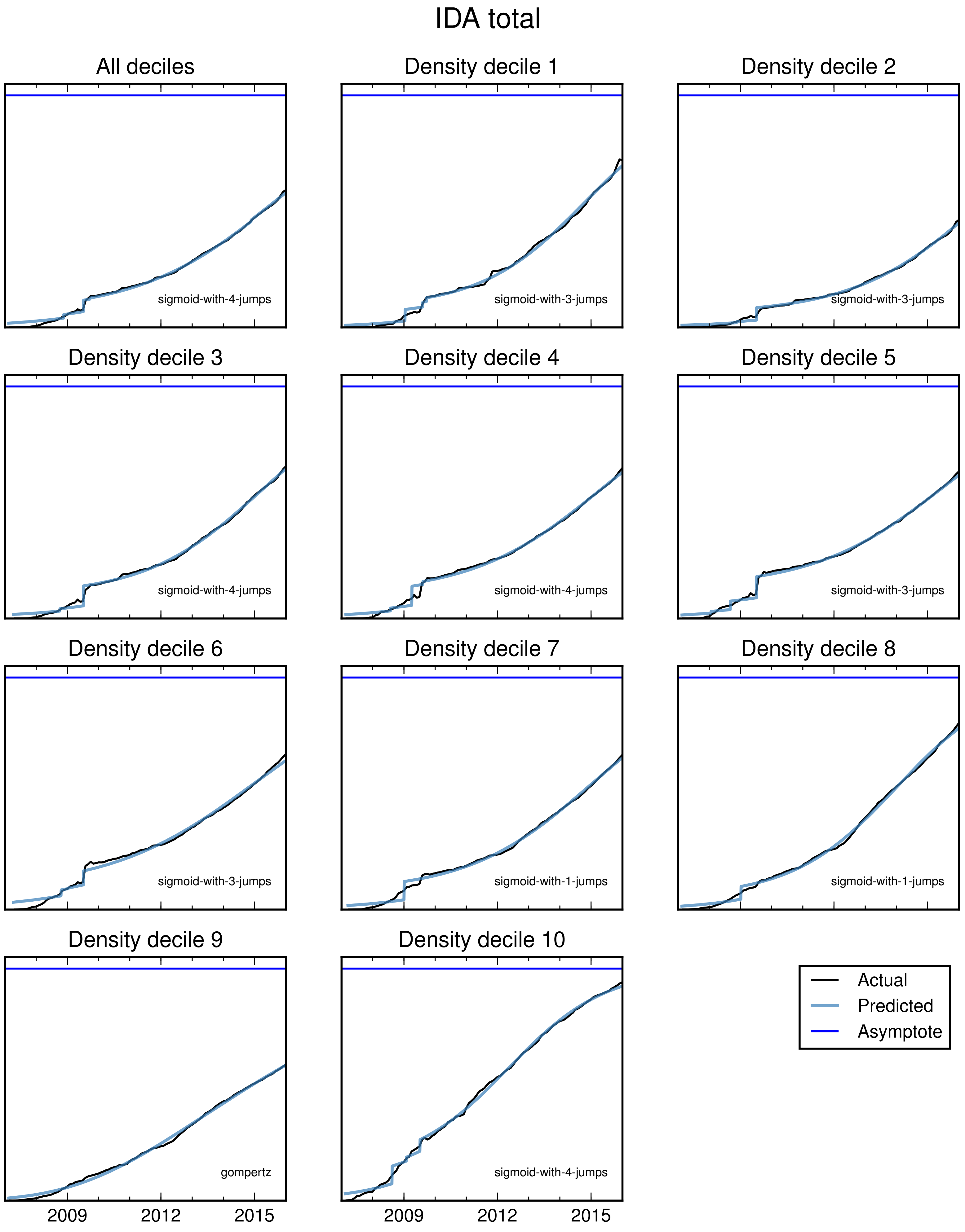
IDA total
All deciles
sigmoid-with-4-jumps
Density decile 1
sigmoid-with-3-jumps
Density decile 2
sigmoid-with-3-jumps
Density decile 3
sigmoid-with-4-jumps
Density decile 4
sigmoid-with-4-jumps
Density decile 5
sigmoid-with-3-jumps
Density decile 6
sigmoid-with-3-jumps
Density decile 7
sigmoid-with-1-jumps
Density decile 8
sigmoid-with-1-jumps
Density decile 9
gompertz
Density decile 10
sigmoid-with-4-jumps
2009
2012
2015
Actual
Predicted
Asymptote

Least developed countries: UN classification

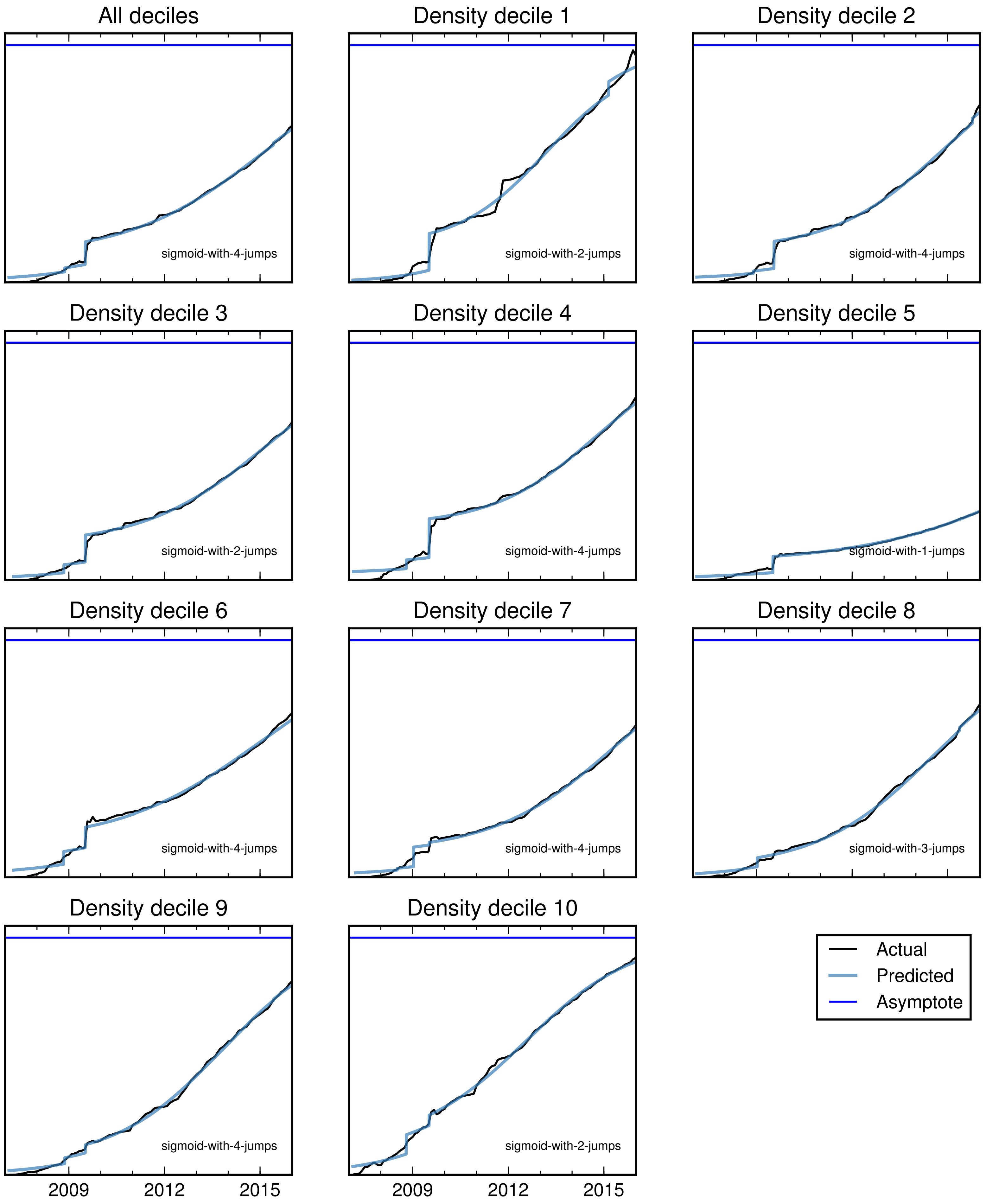

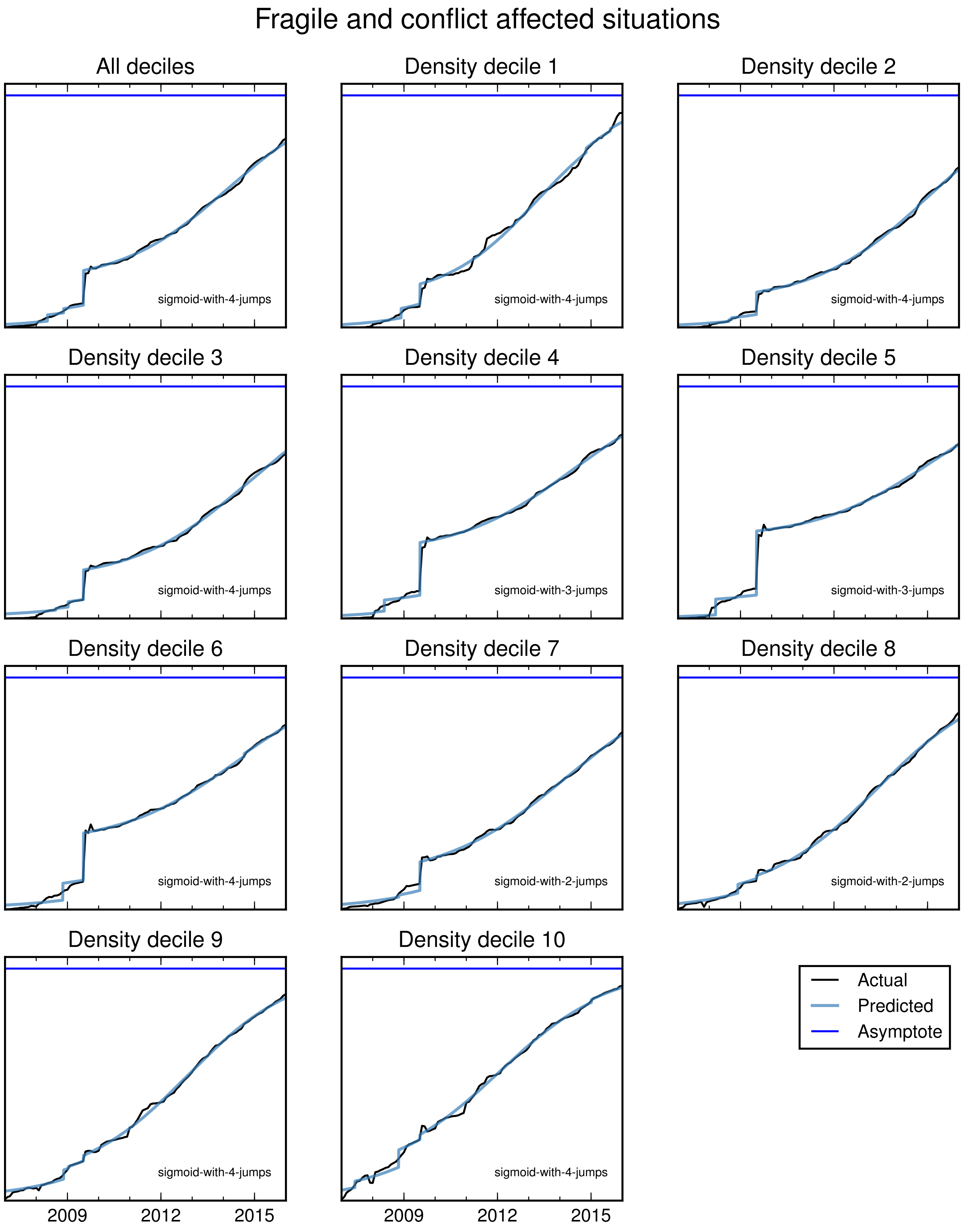
Fragile and conflict affected situations
All deciles
sigmoid-with-4-jumps
Density decile 1
sigmoid-with-4-jumps
Density decile 2
sigmoid-with-4-jumps
Density decile 3
sigmoid-with-4-jumps
Density decile 4
sigmoid-with-3-jumps
Density decile 5
sigmoid-with-3-jumps
Density decile 6
sigmoid-with-4-jumps
Density decile 7
sigmoid-with-2-jumps
Density decile 8
sigmoid-with-2-jumps
Density decile 9
sigmoid-with-4-jumps
Density decile 10
sigmoid-with-4-jumps
2009
2012
2015
2009
2012
2015
Actual
Predicted
Asymptote

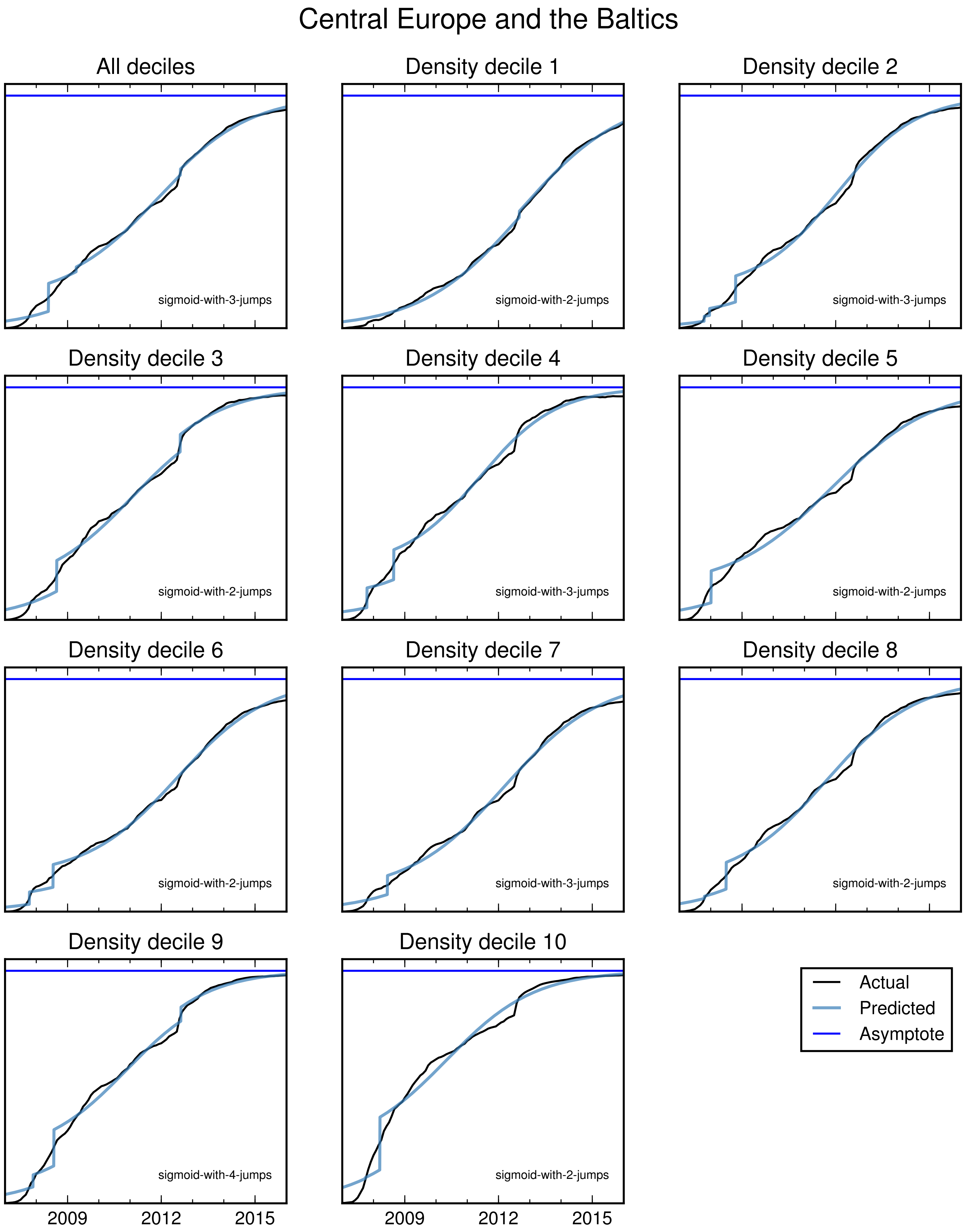

Central Europe and the Baltics
All deciles
sigmoid-with-3-jumps
Density decile 1
sigmoid-with-2-jumps
Density decile 2
sigmoid-with-3-jumps
Density decile 3
sigmoid-with-2-jumps
Density decile 4
sigmoid-with-3-jumps
Density decile 5
sigmoid-with-2-jumps
Density decile 6
sigmoid-with-2-jumps
Density decile 7
sigmoid-with-3-jumps
Density decile 8
sigmoid-with-2-jumps
Density decile 9
sigmoid-with-4-jumps
Density decile 10
sigmoid-with-2-jumps
2009
2012
2015
Actual
Predicted
Asymptote

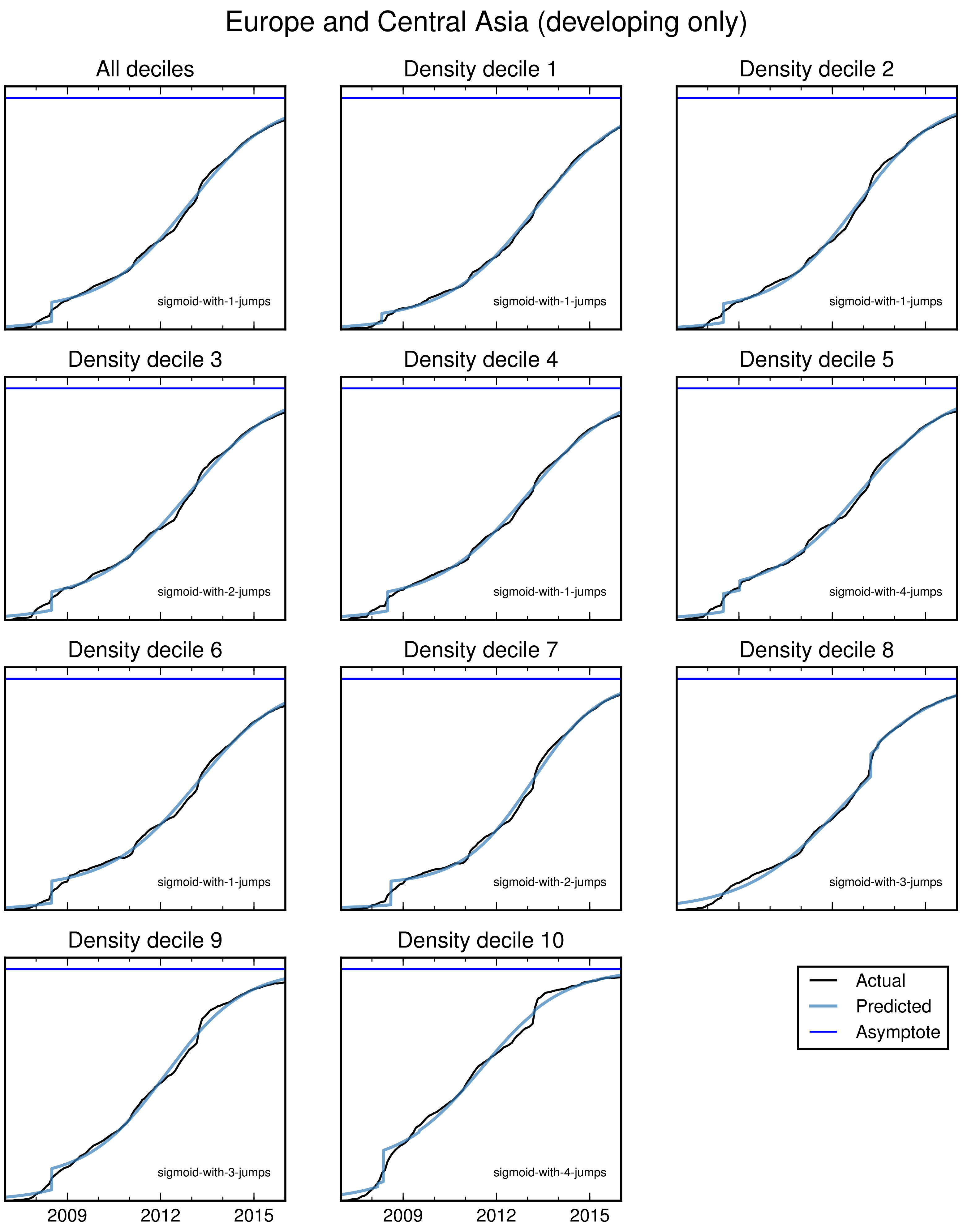
Europe and Central Asia (developing only)
All deciles
sigmoid-with-1-jumps
Density decile 1
sigmoid-with-1-jumps
Density decile 2
sigmoid-with-1-jumps
Density decile 3
sigmoid-with-2-jumps
Density decile 4
sigmoid-with-1-jumps
Density decile 5
sigmoid-with-4-jumps
Density decile 6
sigmoid-with-1-jumps
Density decile 7
sigmoid-with-2-jumps
Density decile 8
sigmoid-with-3-jumps
Density decile 9
sigmoid-with-3-jumps
Density decile 10
sigmoid-with-4-jumps
2009
2012
2015
2009
2012
2015
Actual
Predicted
Asymptote

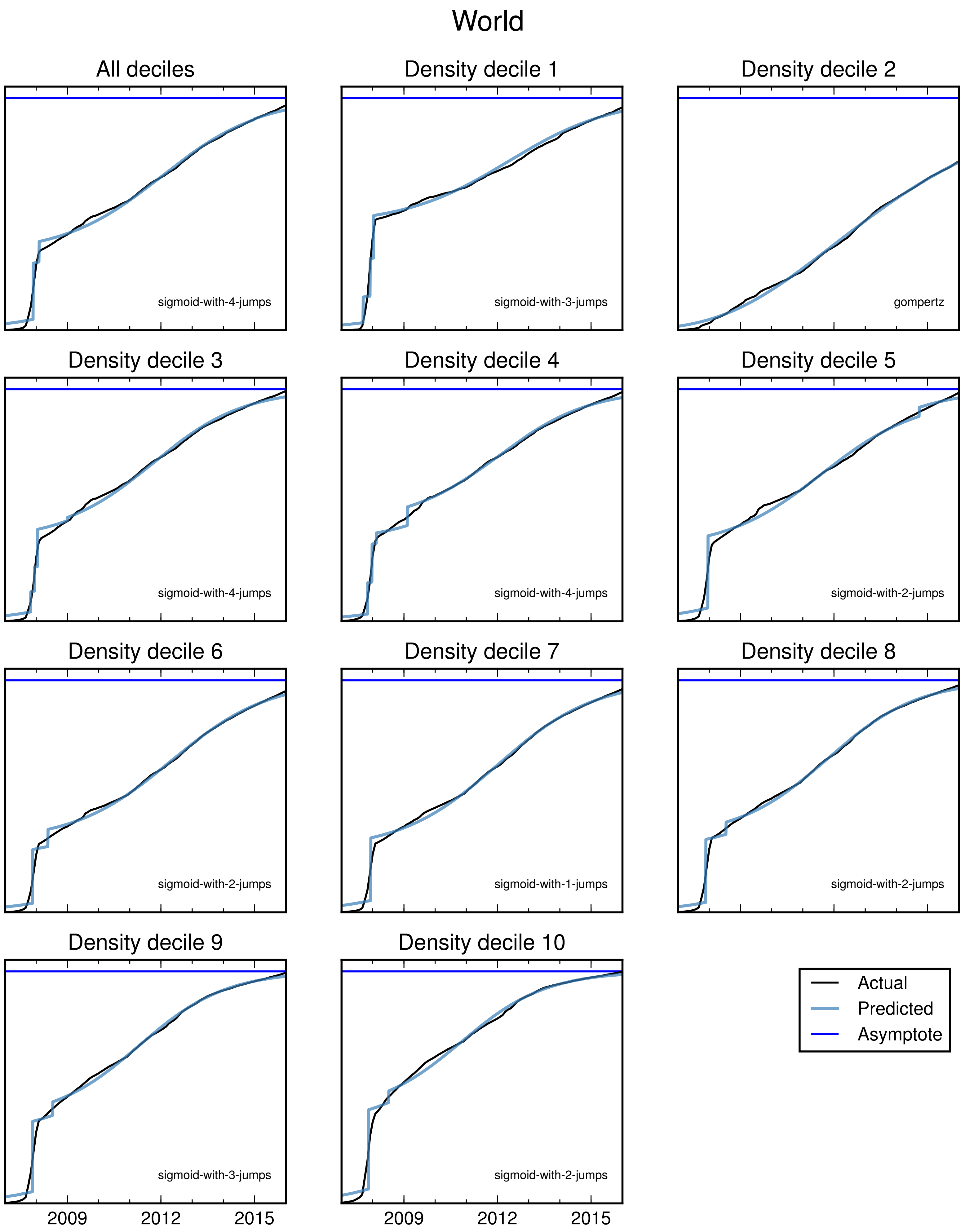
World
All deciles
sigmoid-with-4-jumps
Density decile 1
sigmoid-with-3-jumps
Density decile 2
gompertz
Density decile 3
sigmoid-with-4-jumps
Density decile 4
sigmoid-with-4-jumps
Density decile 5
sigmoid-with-2-jumps
Density decile 6
sigmoid-with-2-jumps
Density decile 7
sigmoid-with-1-jumps
Density decile 8
sigmoid-with-2-jumps
Density decile 9
sigmoid-with-3-jumps
Density decile 10
sigmoid-with-2-jumps
2009
2012
2015
Actual
Predicted
Asymptote

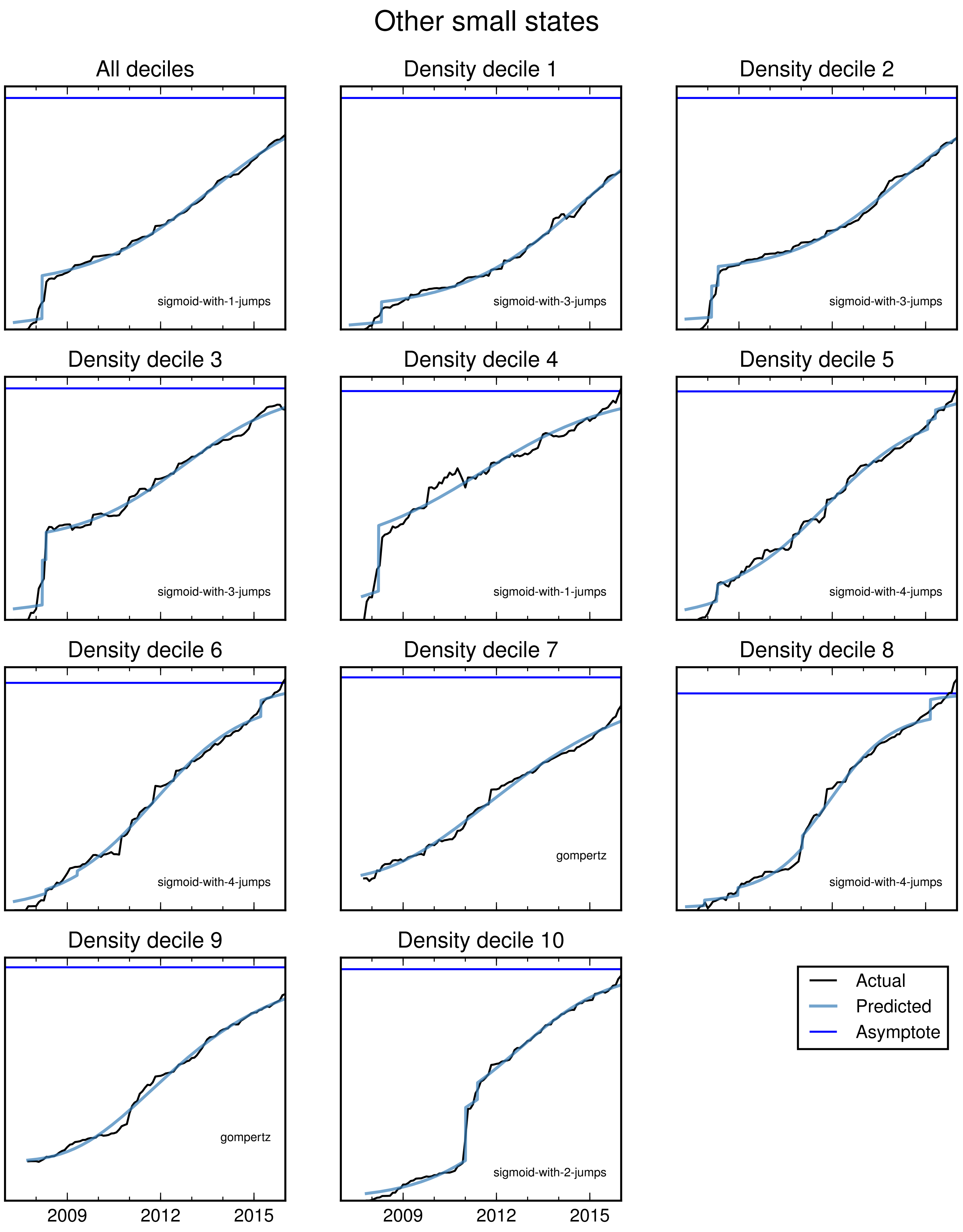
Other small states
All deciles
sigmoid-with-1-jumps
Density decile 1
sigmoid-with-3-jumps
Density decile 2
sigmoid-with-3-jumps
Density decile 3
sigmoid-with-3-jumps
Density decile 4
sigmoid-with-1-jumps
Density decile 5
sigmoid-with-4-jumps
Density decile 6
sigmoid-with-4-jumps
Density decile 7
gompertz
Density decile 8
sigmoid-with-4-jumps
Density decile 9
gompertz
Density decile 10
sigmoid-with-2-jumps
2009
2012
2015
2009
2012
2015
Actual
Predicted
Asymptote

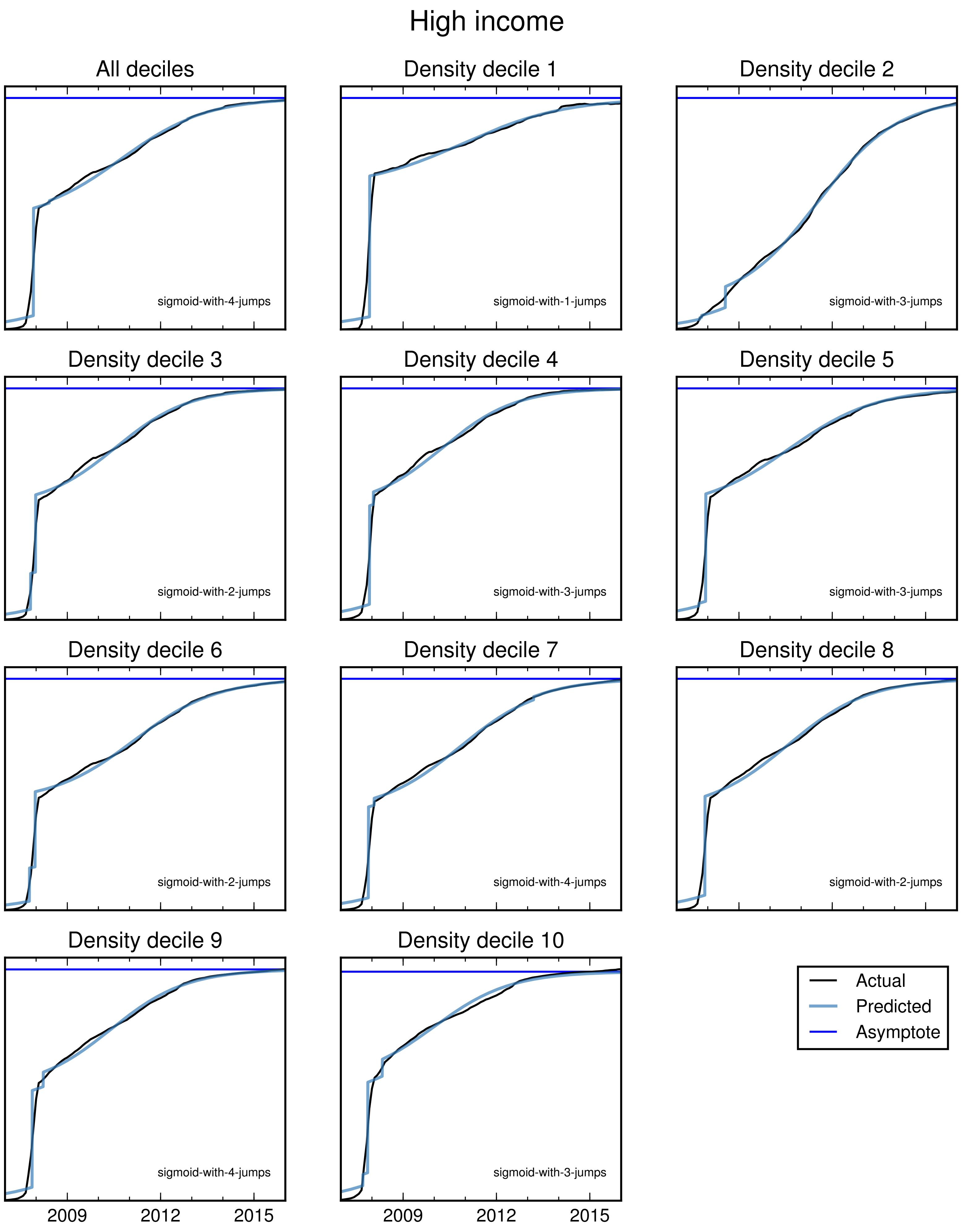
High income
All deciles
sigmoid-with-4-jumps
Density decile 1
sigmoid-with-1-jumps
Density decile 2
sigmoid-with-3-jumps
Density decile 3
sigmoid-with-2-jumps
Density decile 4
sigmoid-with-3-jumps
Density decile 5
sigmoid-with-3-jumps
Density decile 6
sigmoid-with-2-jumps
Density decile 7
sigmoid-with-4-jumps
Density decile 8
sigmoid-with-2-jumps
Density decile 9
sigmoid-with-4-jumps
Density decile 10
sigmoid-with-3-jumps
2009
2012
2015
Actual
Predicted
Asymptote

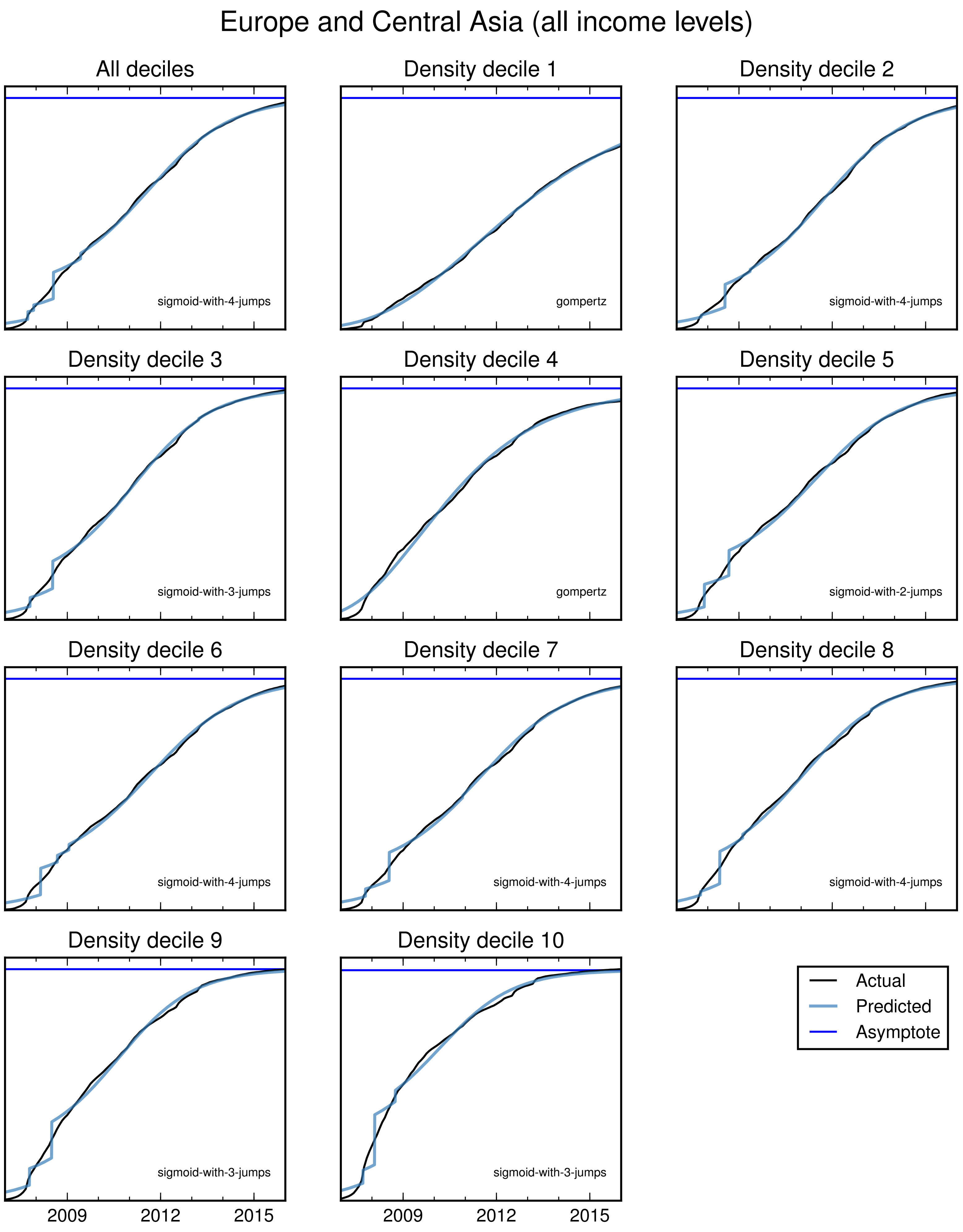
Europe and Central Asia (all income levels)
All deciles
sigmoid-with-4-jumps
Density decile 1
gompertz
Density decile 2
sigmoid-with-4-jumps
Density decile 3
sigmoid-with-3-jumps
Density decile 4
gompertz
Density decile 5
sigmoid-with-2-jumps
Density decile 6
sigmoid-with-4-jumps
Density decile 7
sigmoid-with-4-jumps
Density decile 8
sigmoid-with-4-jumps
Density decile 9
sigmoid-with-3-jumps
Density decile 10
sigmoid-with-3-jumps
2009
2012
2015
Actual
Predicted
Asymptote

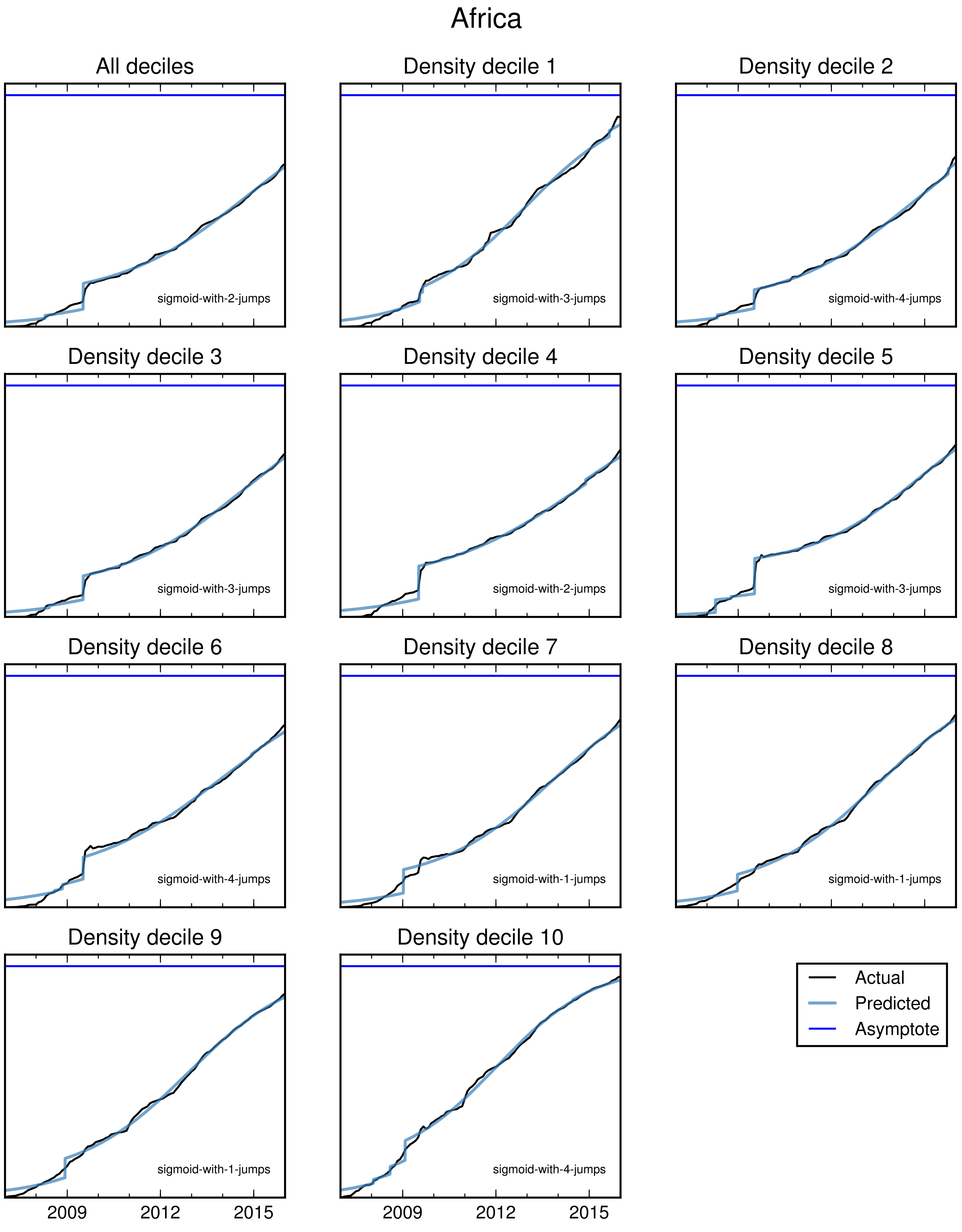
Africa
All deciles
sigmoid-with-2-jumps
Density decile 1
sigmoid-with-3-jumps
Density decile 2
sigmoid-with-4-jumps
Density decile 3
sigmoid-with-3-jumps
Density decile 4
sigmoid-with-2-jumps
Density decile 5
sigmoid-with-3-jumps
Density decile 6
sigmoid-with-4-jumps
Density decile 7
sigmoid-with-1-jumps
Density decile 8
sigmoid-with-1-jumps
Density decile 9
sigmoid-with-1-jumps
Density decile 10
sigmoid-with-4-jumps
2009
2012
2015
2009
2012
2015
Actual
Predicted
Asymptote

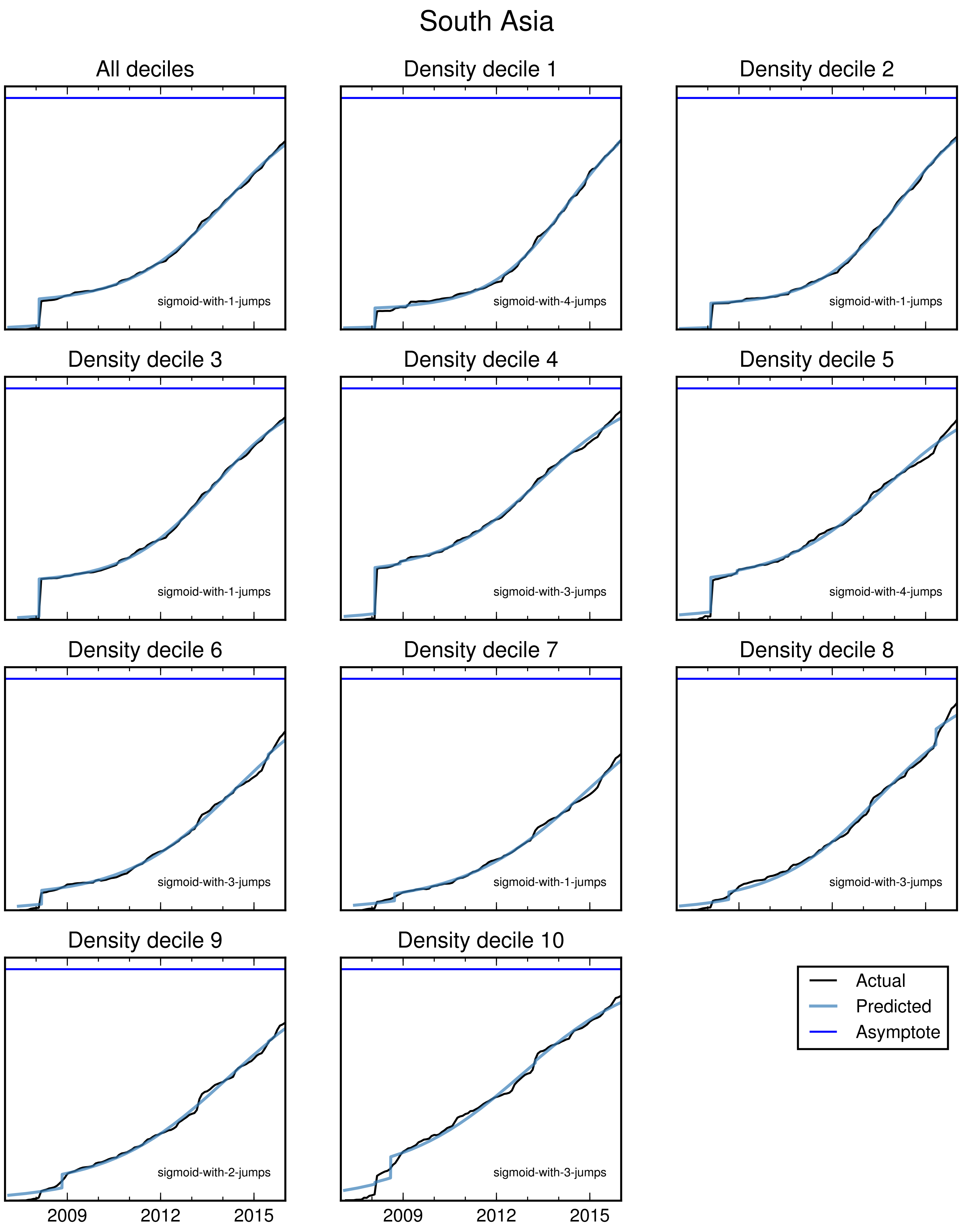
South Asia
All deciles
sigmoid-with-1-jumps
Density decile 1
sigmoid-with-4-jumps
Density decile 2
sigmoid-with-1-jumps
Density decile 3
sigmoid-with-1-jumps
Density decile 4
sigmoid-with-3-jumps
Density decile 5
sigmoid-with-4-jumps
Density decile 6
sigmoid-with-3-jumps
Density decile 7
sigmoid-with-1-jumps
Density decile 8
sigmoid-with-3-jumps
Density decile 9
sigmoid-with-2-jumps
Density decile 10
sigmoid-with-3-jumps
2009
2012
2015
Actual
Predicted
Asymptote

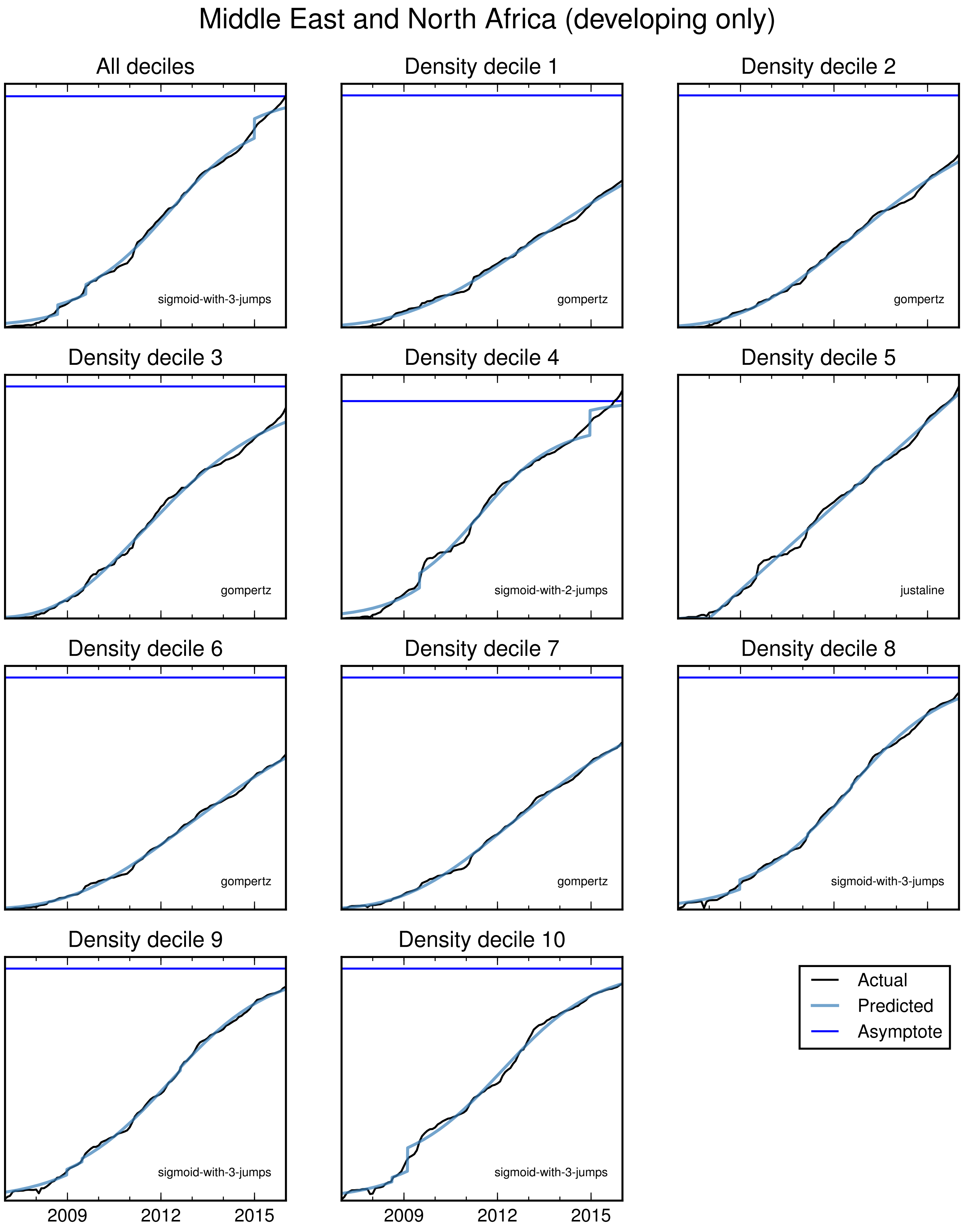
Middle East and North Africa (developing only)
All deciles
sigmoid-with-3-jumps
Density decile 1
gompertz
Density decile 2
gompertz
Density decile 3
gompertz
Density decile 4
sigmoid-with-2-jumps
Density decile 5
justaline
Density decile 6
gompertz
Density decile 7
gompertz
Density decile 8
sigmoid-with-3-jumps
Density decile 9
sigmoid-with-3-jumps
Density decile 10
sigmoid-with-3-jumps
2009
2012
2015
Actual
Predicted
Asymptote

East Asia and Pacific (all income levels)

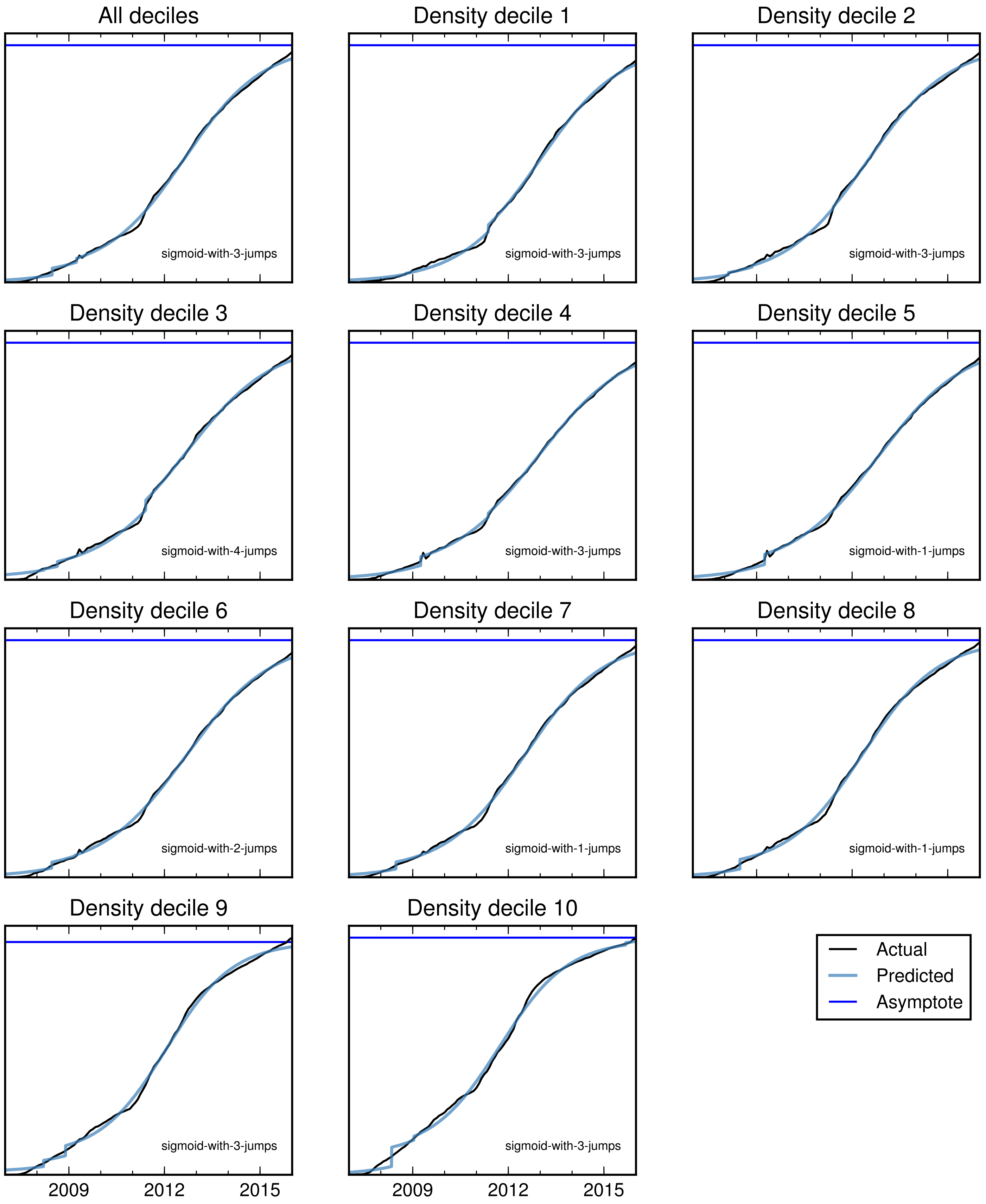

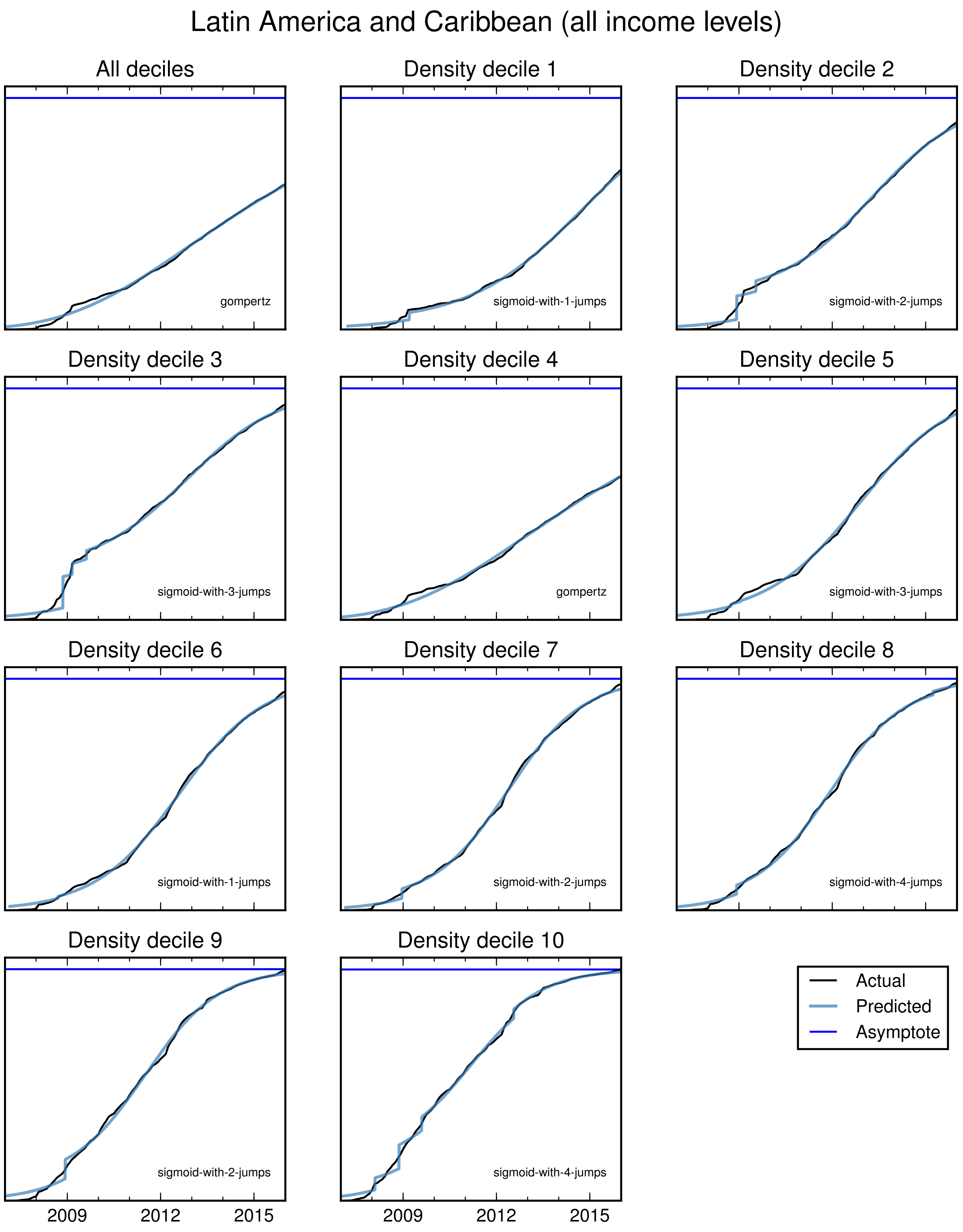

Latin America and Caribbean (all income levels)
All deciles
gompertz
Density decile 1
sigmoid-with-1-jumps
Density decile 2
sigmoid-with-2-jumps
Density decile 3
sigmoid-with-3-jumps
Density decile 4
gompertz
Density decile 5
sigmoid-with-3-jumps
Density decile 6
sigmoid-with-1-jumps
Density decile 7
sigmoid-with-2-jumps
Density decile 8
sigmoid-with-4-jumps
Density decile 9
sigmoid-with-2-jumps
Density decile 10
sigmoid-with-4-jumps
2009
2012
2015
Actual
Predicted
Asymptote

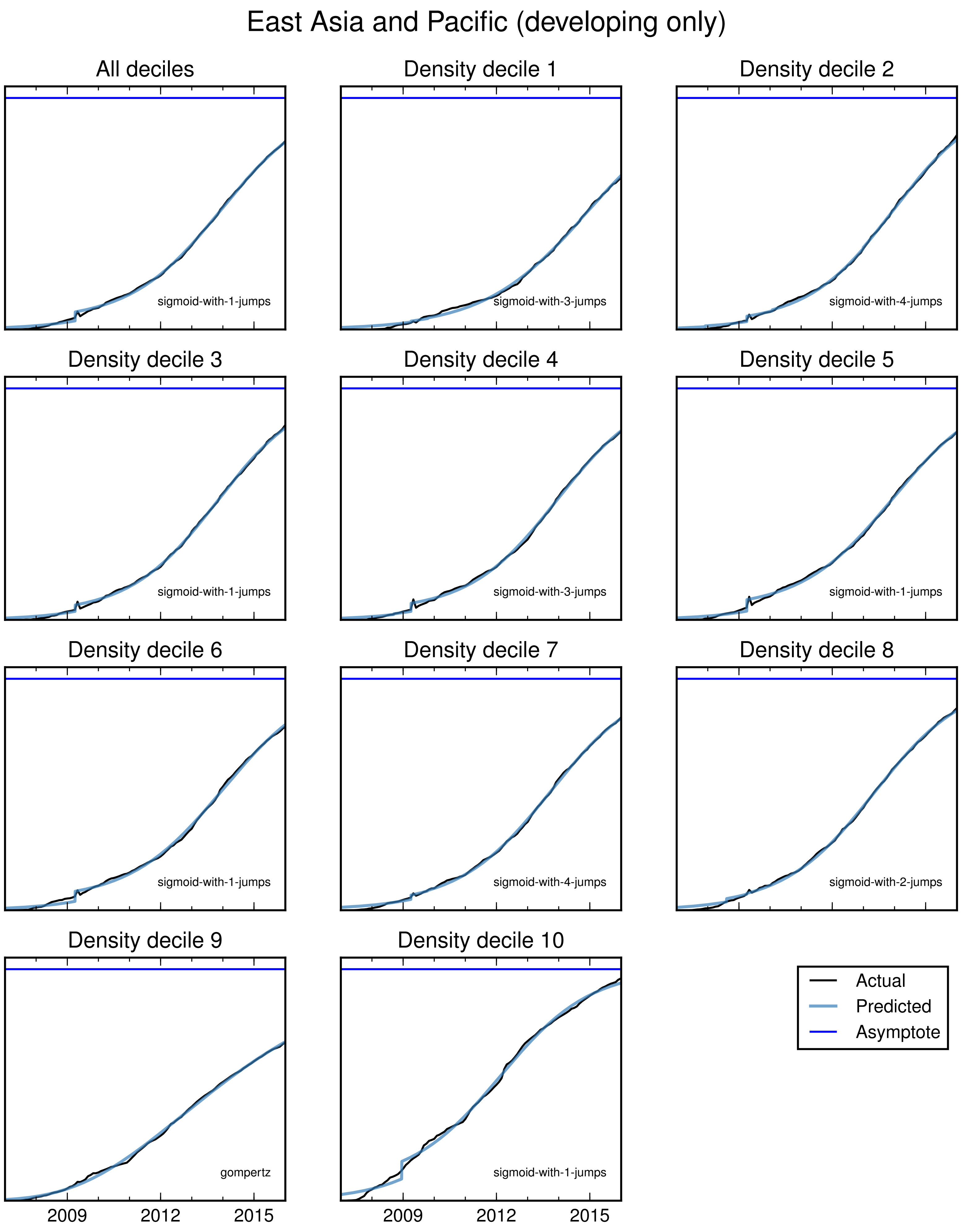
East Asia and Pacific (developing only)
All deciles
sigmoid-with-1-jumps
Density decile 1
sigmoid-with-3-jumps
Density decile 2
sigmoid-with-4-jumps
Density decile 3
sigmoid-with-1-jumps
Density decile 4
sigmoid-with-3-jumps
Density decile 5
sigmoid-with-1-jumps
Density decile 6
sigmoid-with-1-jumps
Density decile 7
sigmoid-with-4-jumps
Density decile 8
sigmoid-with-2-jumps
Density decile 9
gompertz
Density decile 10
sigmoid-with-1-jumps
2009
2012
2015
2009
2012
2015
Actual
Predicted
Asymptote

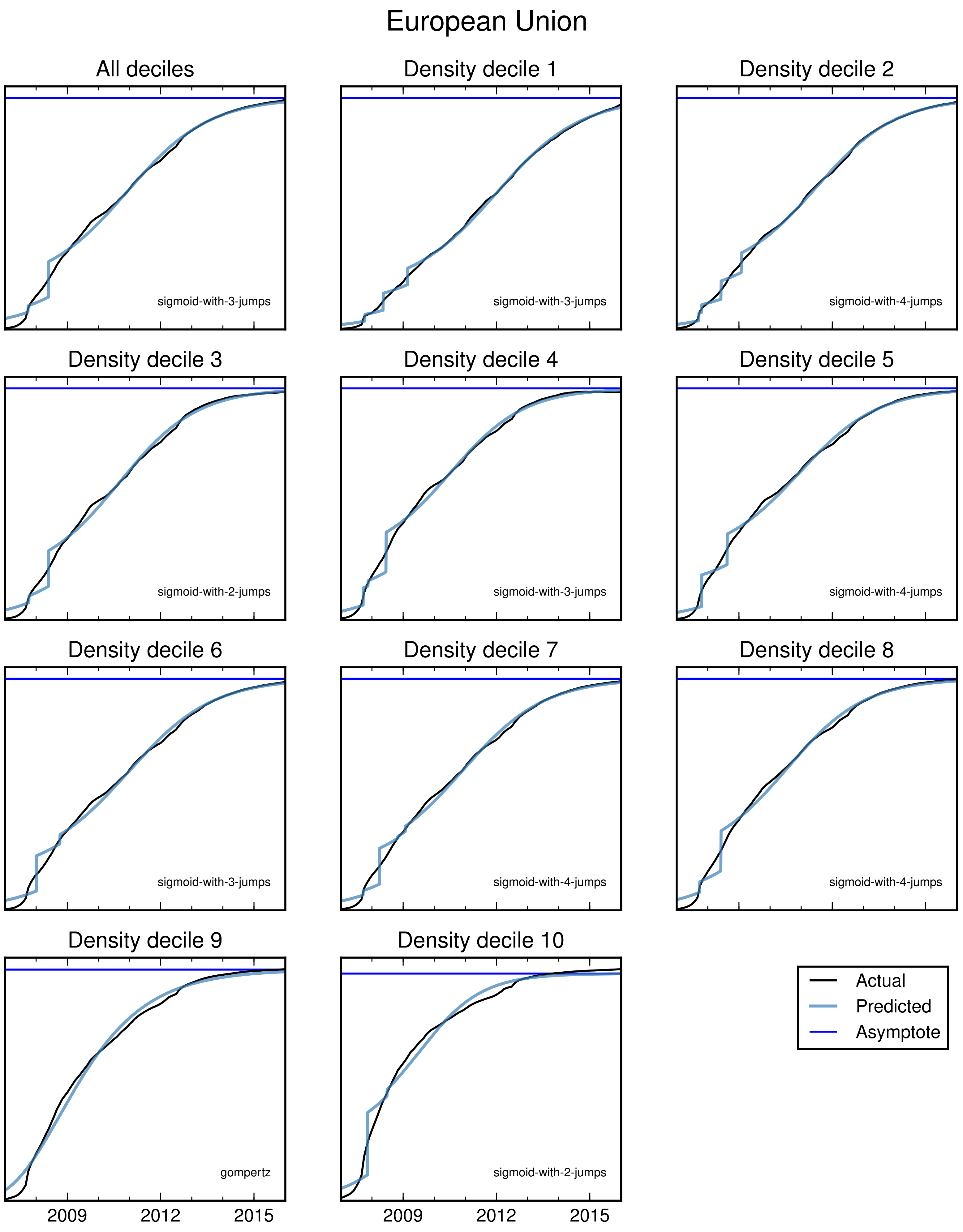
European Union
All deciles
sigmoid-with-3-jumps
Density decile 1
sigmoid-with-3-jumps
Density decile 2
sigmoid-with-4-jumps
Density decile 3
sigmoid-with-2-jumps
Density decile 4
sigmoid-with-3-jumps
Density decile 5
sigmoid-with-4-jumps
Density decile 6
sigmoid-with-3-jumps
Density decile 7
sigmoid-with-4-jumps
Density decile 8
sigmoid-with-4-jumps
Density decile 9
gompertz
Density decile 10
sigmoid-with-2-jumps
2009
2012
2015
Actual
Predicted
Asymptote

IDA and IBRD total

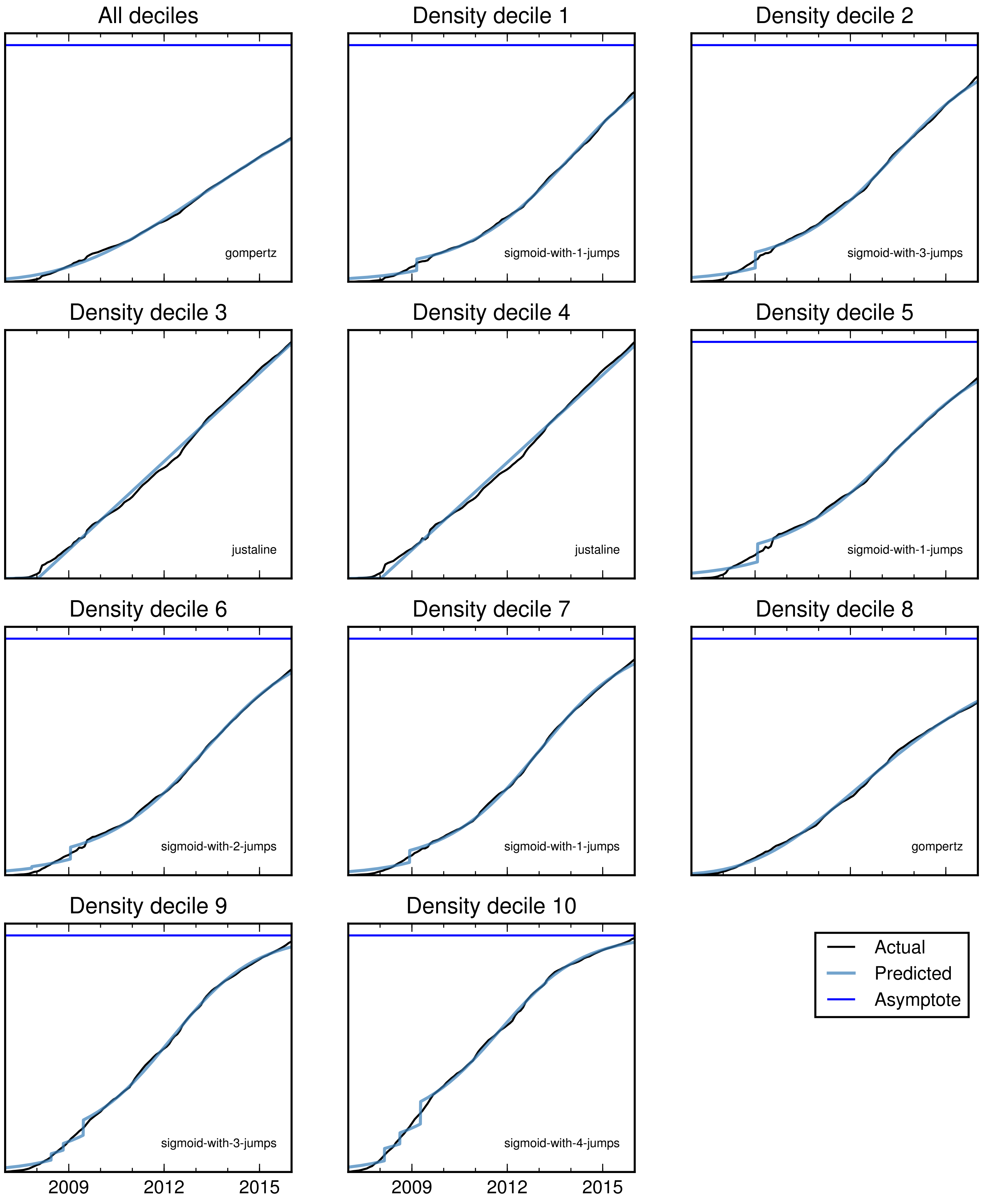

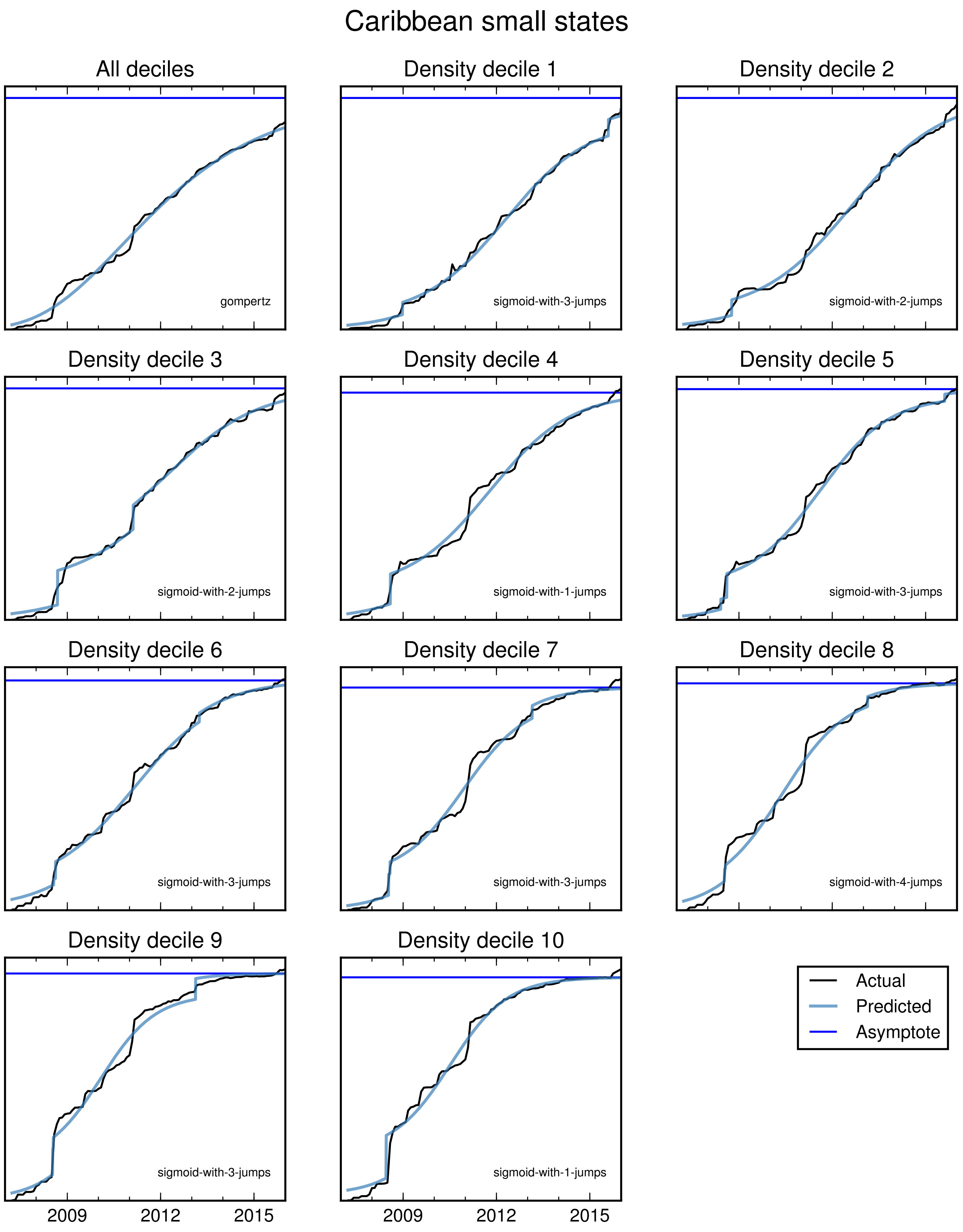
Caribbean small states
All deciles
gompertz
Density decile 1
sigmoid-with-3-jumps
Density decile 2
sigmoid-with-2-jumps
Density decile 3
sigmoid-with-2-jumps
Density decile 4
sigmoid-with-1-jumps
Density decile 5
sigmoid-with-3-jumps
Density decile 6
sigmoid-with-3-jumps
Density decile 7
sigmoid-with-3-jumps
Density decile 8
sigmoid-with-4-jumps
Density decile 9
sigmoid-with-3-jumps
Density decile 10
sigmoid-with-1-jumps
2009
2012
2015
Actual
Predicted
Asymptote

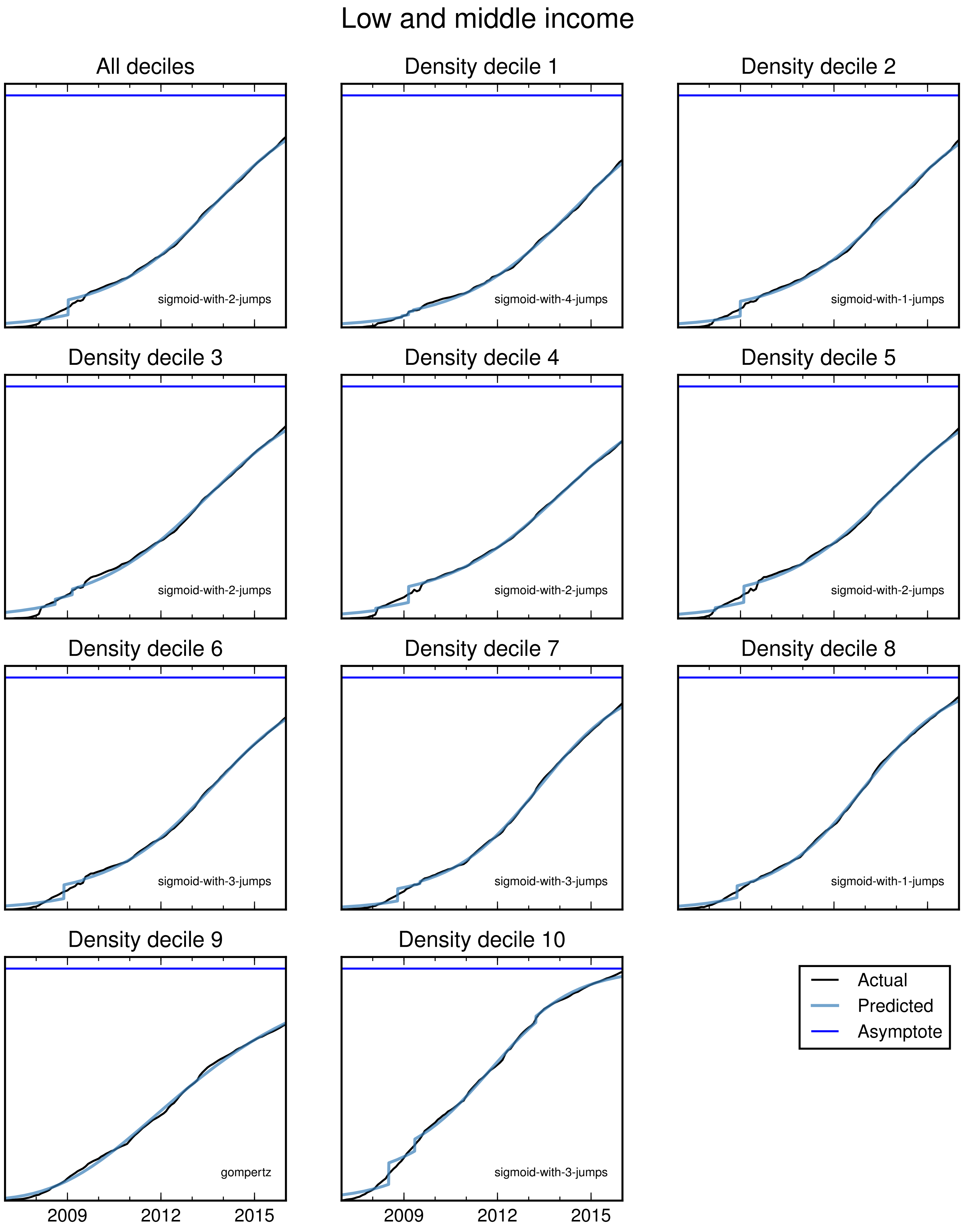
Low and middle income
All deciles
sigmoid-with-2-jumps
Density decile 1
sigmoid-with-4-jumps
Density decile 2
sigmoid-with-1-jumps
Density decile 3
sigmoid-with-2-jumps
Density decile 4
sigmoid-with-2-jumps
Density decile 5
sigmoid-with-2-jumps
Density decile 6
sigmoid-with-3-jumps
Density decile 7
sigmoid-with-3-jumps
Density decile 8
sigmoid-with-1-jumps
Density decile 9
gompertz
Density decile 10
sigmoid-with-3-jumps
2009
2012
2015
Actual
Predicted
Asymptote

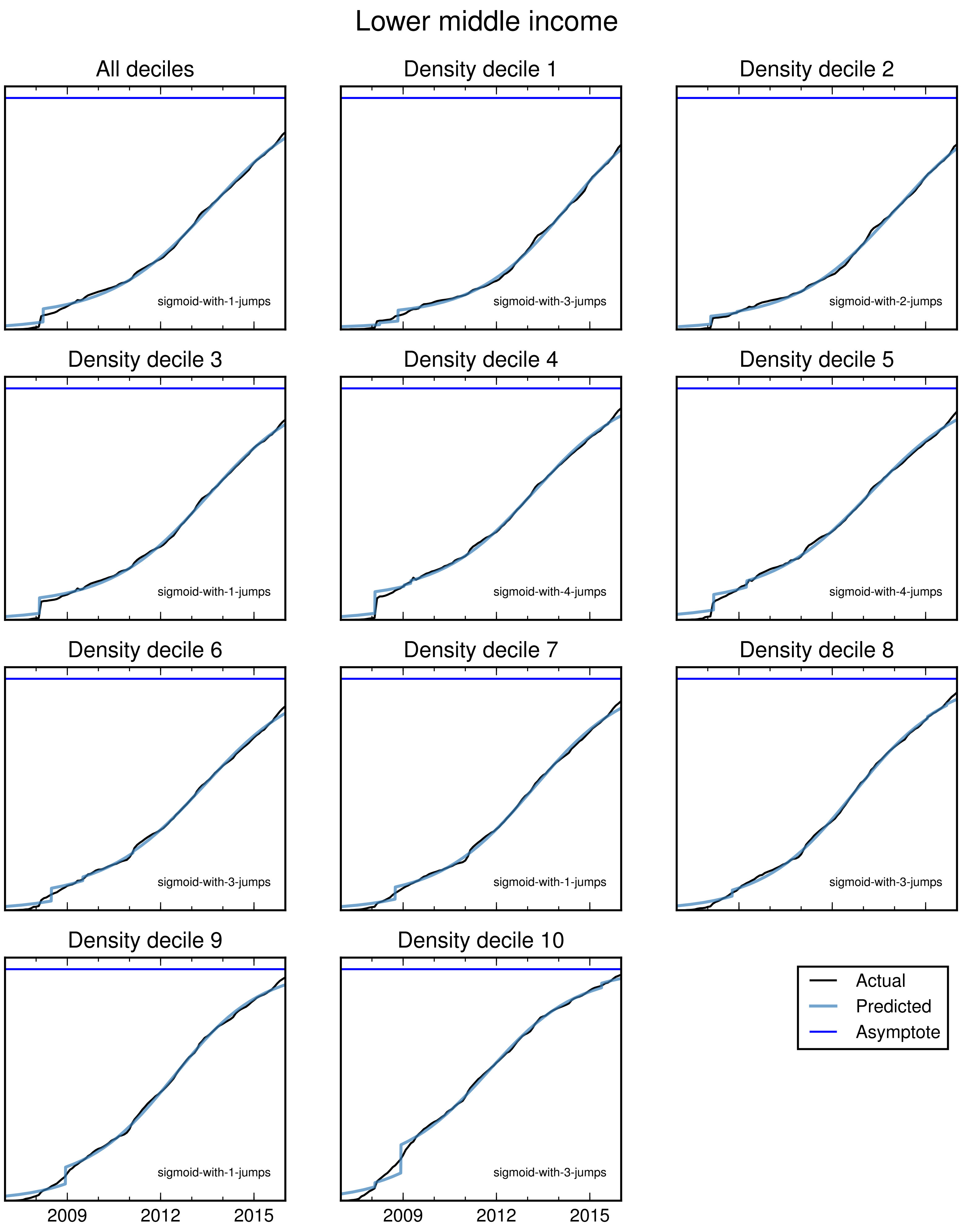
Lower middle income
All deciles
sigmoid-with-1-jumps
Density decile 1
sigmoid-with-3-jumps
Density decile 2
sigmoid-with-2-jumps
Density decile 3
sigmoid-with-1-jumps
Density decile 4
sigmoid-with-4-jumps
Density decile 5
sigmoid-with-4-jumps
Density decile 6
sigmoid-with-3-jumps
Density decile 7
sigmoid-with-1-jumps
Density decile 8
sigmoid-with-3-jumps
Density decile 9
sigmoid-with-1-jumps
Density decile 10
sigmoid-with-3-jumps
2009
2012
2015
2009
2012
2015
Actual
Predicted
Asymptote

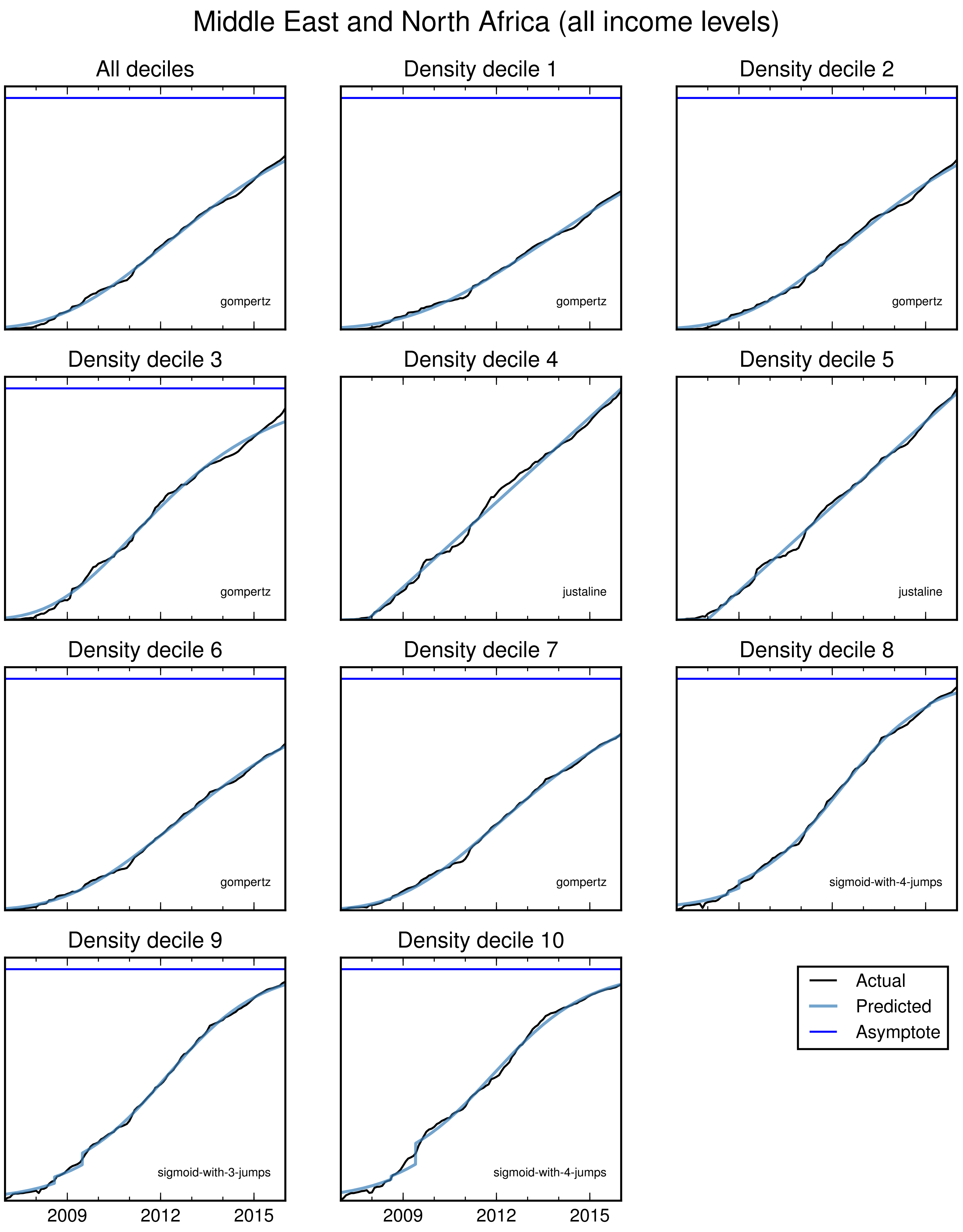
Middle East and North Africa (all income levels)
All deciles
gompertz
Density decile 1
gompertz
Density decile 2
gompertz
Density decile 3
gompertz
Density decile 4
justaline
Density decile 5
justaline
Density decile 6
gompertz
Density decile 7
gompertz
Density decile 8
sigmoid-with-4-jumps
Density decile 9
sigmoid-with-3-jumps
Density decile 10
sigmoid-with-4-jumps
2009
2012
2015
Actual
Predicted
Asymptote

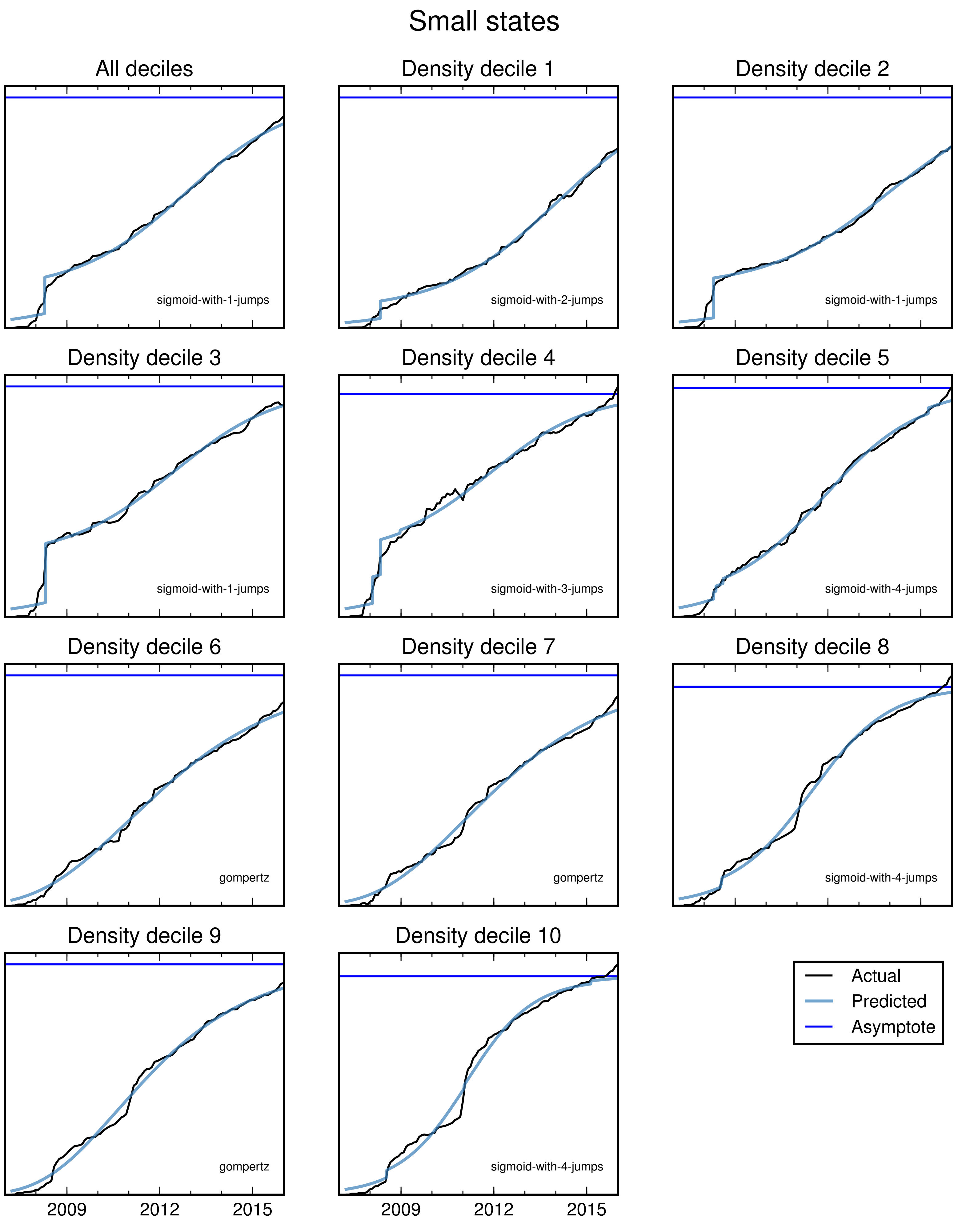

Small states
All deciles
sigmoid-with-1-jumps
Density decile 1
sigmoid-with-2-jumps
Density decile 2
sigmoid-with-1-jumps
Density decile 3
sigmoid-with-1-jumps
Density decile 4
sigmoid-with-3-jumps
Density decile 5
sigmoid-with-4-jumps
Density decile 6
gompertz
Density decile 7
gompertz
Density decile 8
sigmoid-with-4-jumps
Density decile 9
gompertz
Density decile 10
sigmoid-with-4-jumps
2009
2012
2015
2009
2012
2015
Actual
Predicted
Asymptote

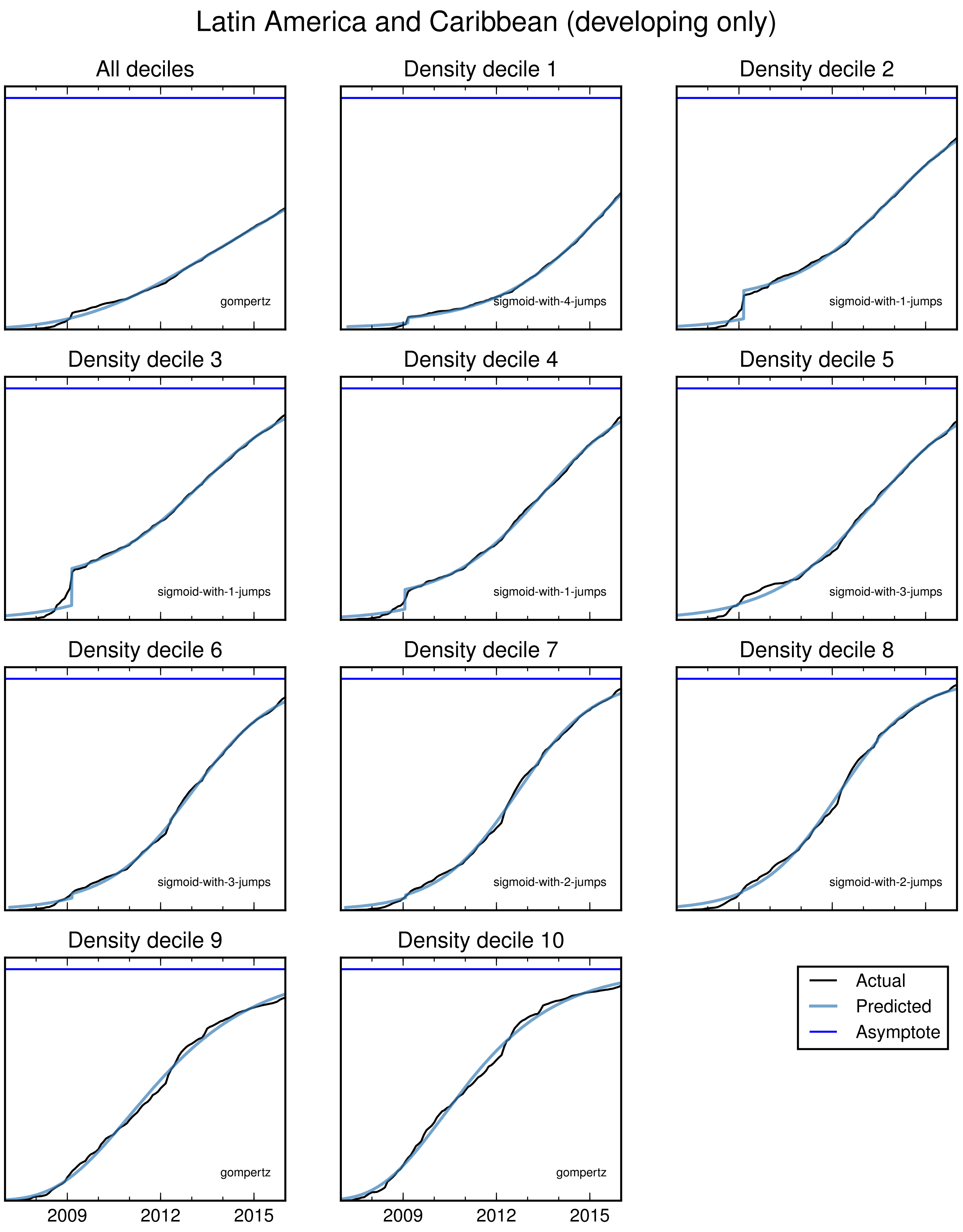
Latin America and Caribbean (developing only)
All deciles
gompertz
Density decile 1
sigmoid-with-4-jumps
Density decile 2
sigmoid-with-1-jumps
Density decile 3
sigmoid-with-1-jumps
Density decile 4
sigmoid-with-1-jumps
Density decile 5
sigmoid-with-3-jumps
Density decile 6
sigmoid-with-3-jumps
Density decile 7
sigmoid-with-2-jumps
Density decile 8
sigmoid-with-2-jumps
Density decile 9
gompertz
Density decile 10
gompertz
2009
2012
2015
Actual
Predicted
Asymptote

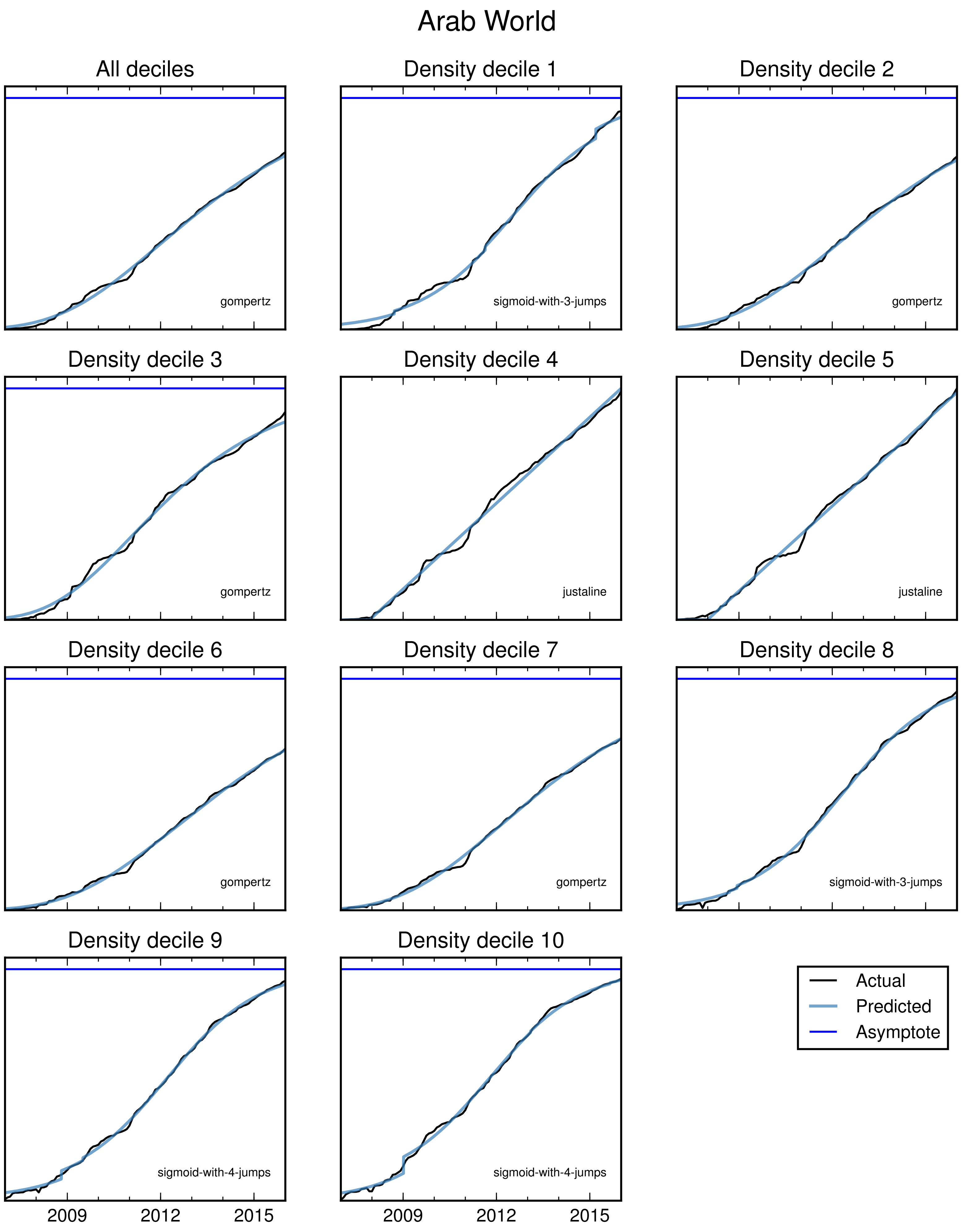
Arab World
All deciles
gompertz
Density decile 1
sigmoid-with-3-jumps
Density decile 2
gompertz
Density decile 3
gompertz
Density decile 4
justaline
Density decile 5
justaline
Density decile 6
gompertz
Density decile 7
gompertz
Density decile 8
sigmoid-with-3-jumps
Density decile 9
sigmoid-with-4-jumps
Density decile 10
sigmoid-with-4-jumps
2009
2012
2015
Actual
Predicted
Asymptote

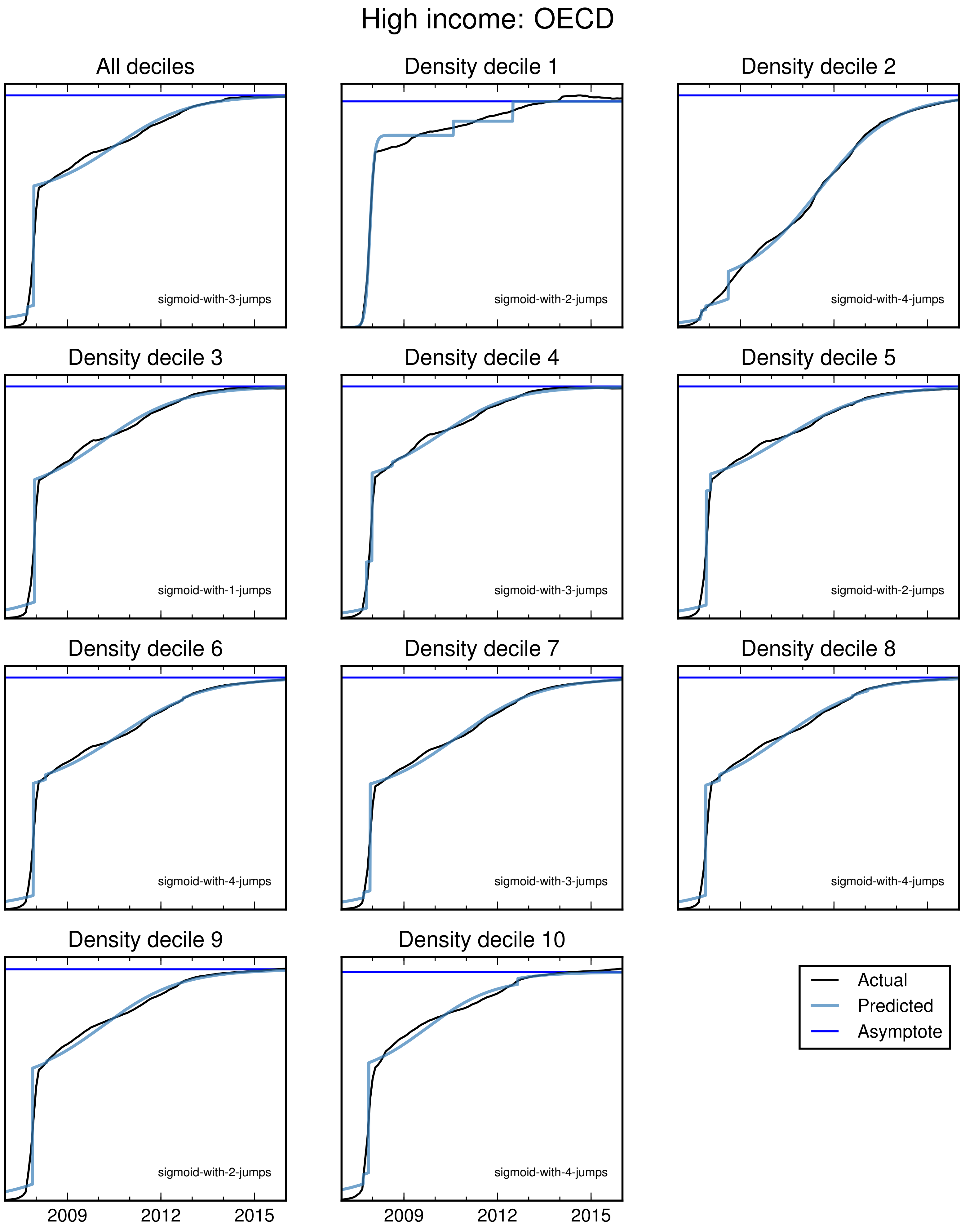
High income: OECD
All deciles
sigmoid-with-3-jumps
Density decile 1
sigmoid-with-2-jumps
Density decile 2
sigmoid-with-4-jumps
Density decile 3
sigmoid-with-1-jumps
Density decile 4
sigmoid-with-3-jumps
Density decile 5
sigmoid-with-2-jumps
Density decile 6
sigmoid-with-4-jumps
Density decile 7
sigmoid-with-3-jumps
Density decile 8
sigmoid-with-4-jumps
Density decile 9
sigmoid-with-2-jumps
Density decile 10
sigmoid-with-4-jumps
2009
2012
2015
Actual
Predicted
Asymptote

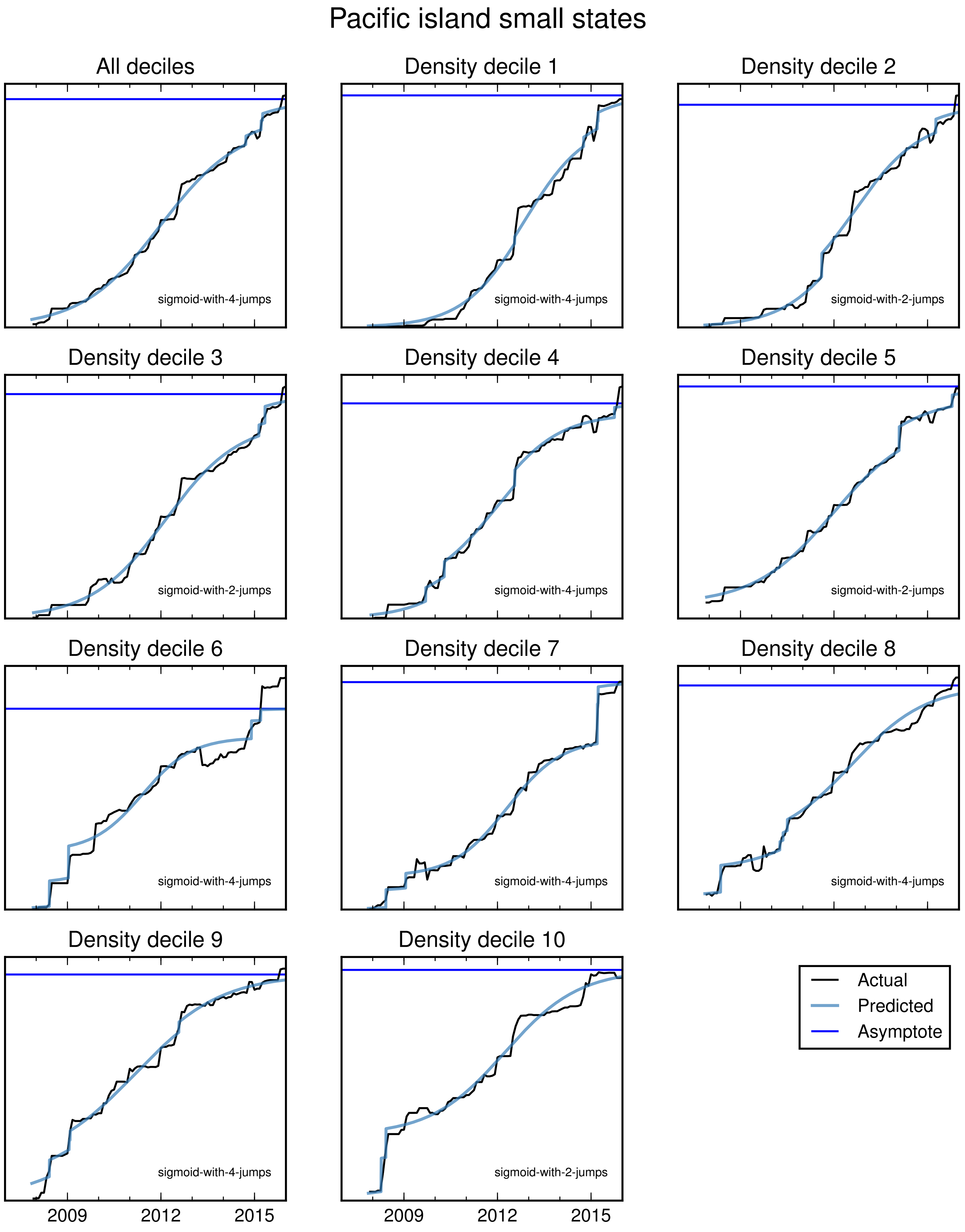
Pacific island small states
All deciles
Density decile 1
Density decile 2
Density decile 3
Density decile 4
Density decile 5
Density decile 6
Density decile 7
Density decile 8
Density decile 9
Density decile 10
sigmoid-with-4-jumps
sigmoid-with-2-jumps
2009
2012
2015
Actual
Predicted
Asymptote

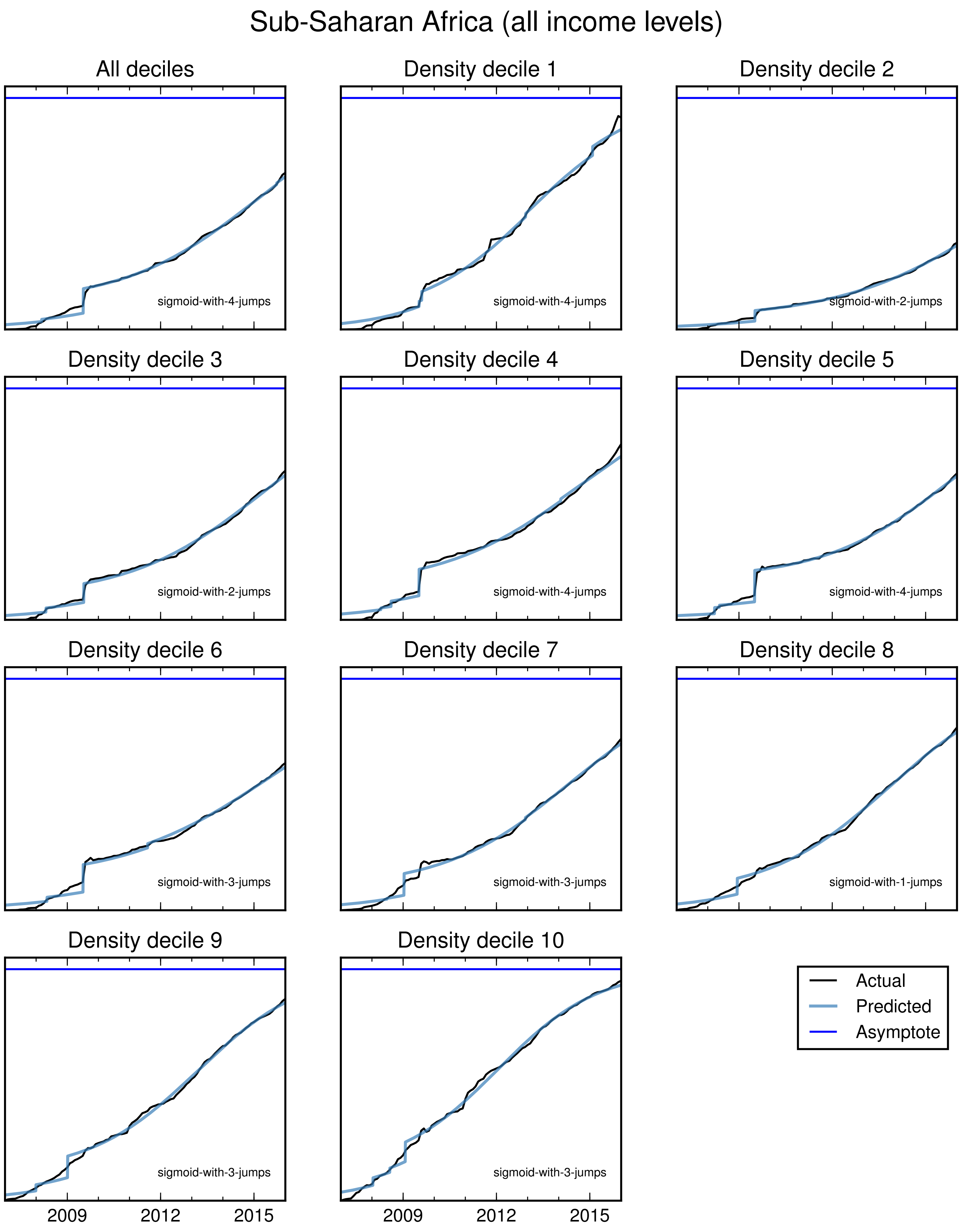

Sub-Saharan Africa (all income levels)
All deciles
sigmoid-with-4-jumps
Density decile 1
sigmoid-with-4-jumps
Density decile 2
sigmoid-with-2-jumps
Density decile 3
sigmoid-with-2-jumps
Density decile 4
sigmoid-with-4-jumps
Density decile 5
sigmoid-with-4-jumps
Density decile 6
sigmoid-with-3-jumps
Density decile 7
sigmoid-with-3-jumps
Density decile 8
sigmoid-with-1-jumps
Density decile 9
sigmoid-with-3-jumps
Density decile 10
sigmoid-with-3-jumps
2009
2012
2015
Actual
Predicted
Asymptote

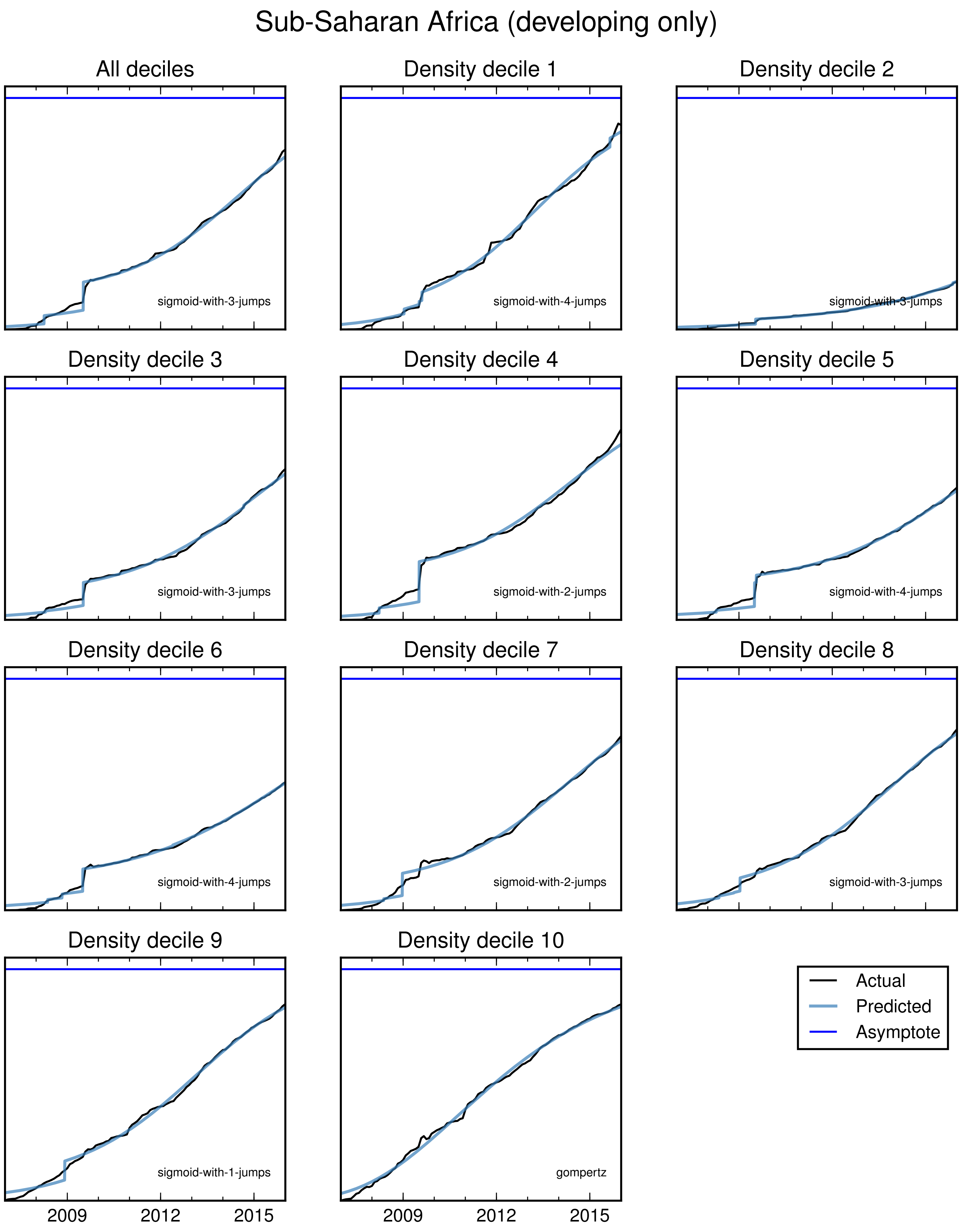

Sub-Saharan Africa (developing only)
All deciles
sigmoid-with-3-jumps
Density decile 1
sigmoid-with-4-jumps
Density decile 2
sigmoid-with-3-jumps
Density decile 3
sigmoid-with-3-jumps
Density decile 4
sigmoid-with-2-jumps
Density decile 5
sigmoid-with-4-jumps
Density decile 6
sigmoid-with-4-jumps
Density decile 7
sigmoid-with-2-jumps
Density decile 8
sigmoid-with-3-jumps
Density decile 9
sigmoid-with-1-jumps
Density decile 10
gompertz
2009
2012
2015
Actual
Predicted
Asymptote

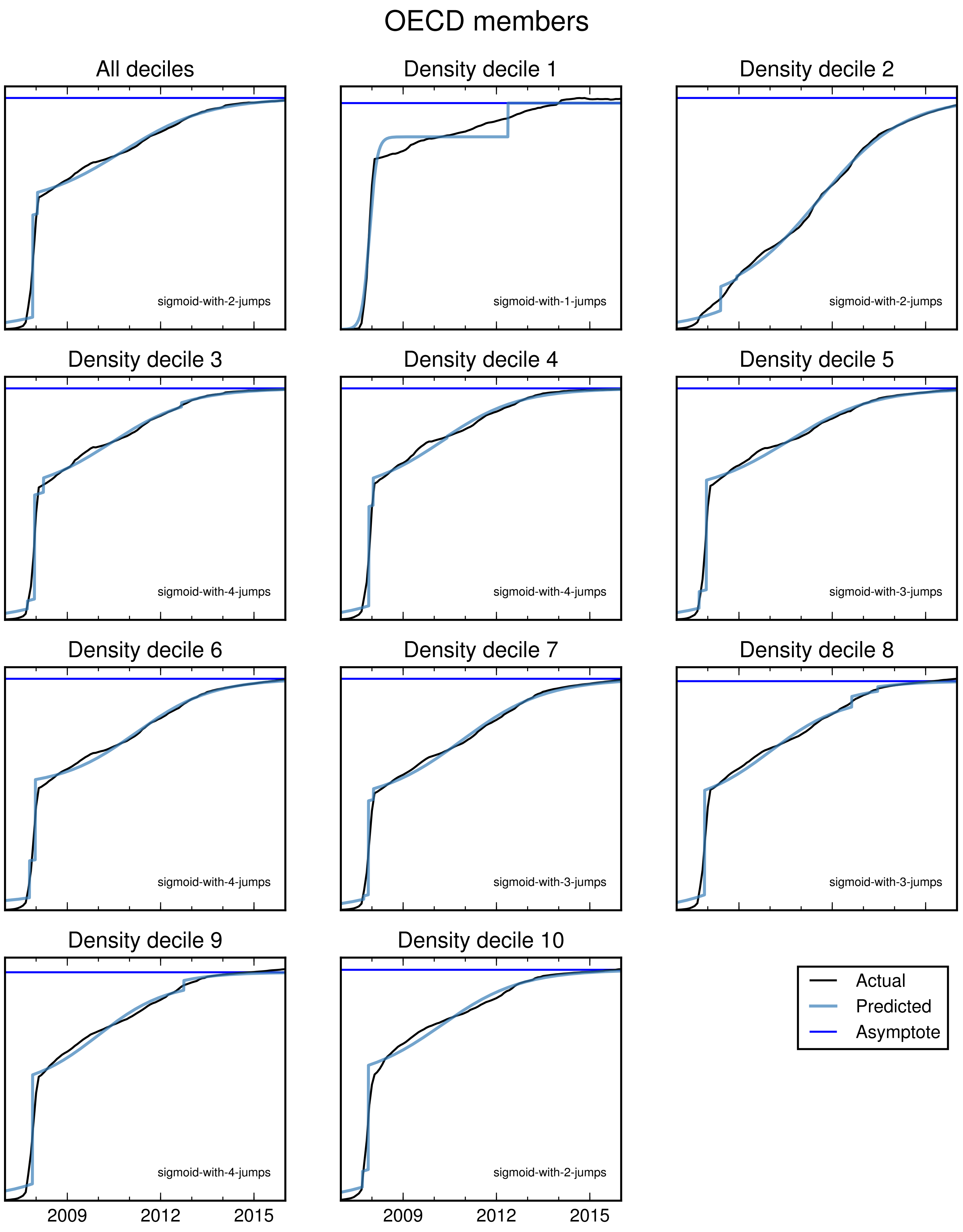
OECD members
All deciles
sigmoid-with-2-jumps
Density decile 1
sigmoid-with-1-jumps
Density decile 2
sigmoid-with-2-jumps
Density decile 3
sigmoid-with-4-jumps
Density decile 4
sigmoid-with-4-jumps
Density decile 5
sigmoid-with-3-jumps
Density decile 6
sigmoid-with-4-jumps
Density decile 7
sigmoid-with-3-jumps
Density decile 8
sigmoid-with-3-jumps
Density decile 9
sigmoid-with-4-jumps
Density decile 10
sigmoid-with-2-jumps
2009
2012
2015
Actual
Predicted
Asymptote

G Multilevel model fits: table of standardized coefficients

model	coefficient	variable	posterior-mean	5th-percentile	95th-percentile
Fraction-complete	intercept	intercept	1.13	0.656	1.65
Fraction-complete	intercept	log-GDP-pc	0.0425	-0.839	0.893
Fraction-complete	intercept	internet	1.18	0.281	2.1
Fraction-complete	intercept	log-pop	-0.565	-1.08	-0.0336
Fraction-complete	intercept	governance	0.917	0.28	1.5
Fraction-complete	log-density	intercept	0.453	-0.295	1.21
Fraction-complete	log-density	log-GDP-pc	0.0459	-1.1	1.22
Fraction-complete	log-density	internet	-0.556	-1.84	0.662
Fraction-complete	log-density	log-pop	0.302	-0.487	1.07
Fraction-complete	log-density	governance	-0.0162	-0.847	0.888
Fraction-complete	log-density-sq	intercept	0.939	0.579	1.31
Fraction-complete	log-density-sq	log-GDP-pc	-0.204	-0.82	0.409
Fraction-complete	log-density-sq	internet	0.366	-0.266	1.01
Fraction-complete	log-density-sq	log-pop	-0.117	-0.53	0.29
Fraction-complete	log-density-sq	governance	-0.063	-0.5	0.385
N-segments	intercept	intercept	2.56	2.42	2.7
N-segments	intercept	log-GDP-pc	0.395	0.133	0.656
N-segments	intercept	internet	0.316	-0.00503	0.629
N-segments	intercept	log-pop	-0.113	-0.249	0.0259
N-segments	intercept	governance	0.103	-0.0791	0.29
N-segments	log-density	intercept	3.1	2.88	3.34
N-segments	log-density	log-GDP-pc	-0.441	-0.87	-0.0119
N-segments	log-density	internet	-0.479	-0.977	0.0132
N-segments	log-density	log-pop	0.341	0.109	0.571
N-segments	log-density	governance	0.000787	-0.31	0.293
N-segments	log-density-sq	intercept	-0.765	-0.889	-0.641
N-segments	log-density-sq	log-GDP-pc	0.154	-0.0874	0.397
N-segments	log-density-sq	internet	0.173	-0.0965	0.439
N-segments	log-density-sq	log-pop	-0.158	-0.28	-0.0292
N-segments	log-density-sq	governance	0.0216	-0.146	0.189

H Multilevel model fits: diagnostics and distribution

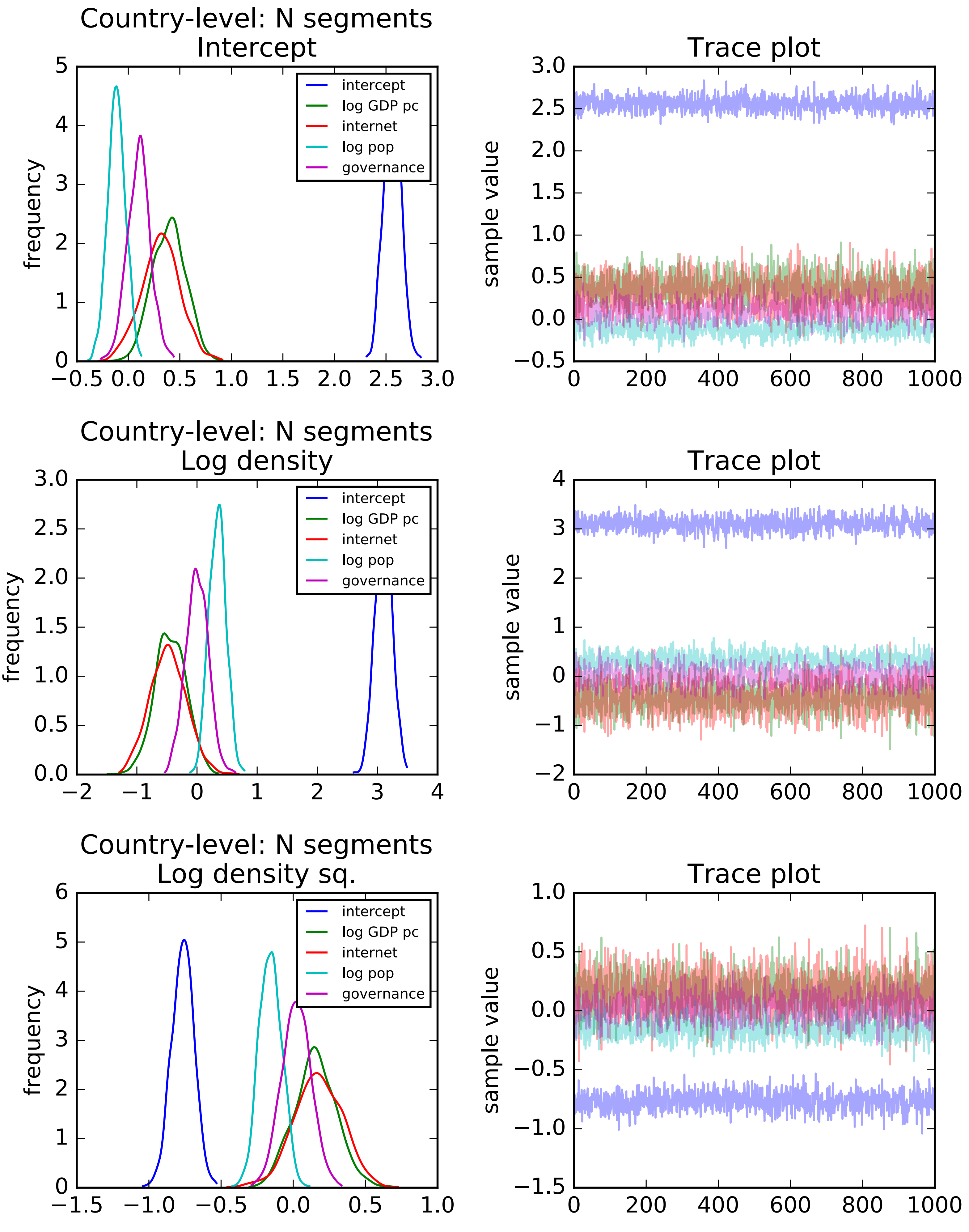
Country-level: N segments
Intercept
frequency
intercept
log GDP pc
internet
log pop
governance
Trace plot
sample value
Country-level: N segments
Log density
frequency
intercept
log GDP pc
internet
log pop
governance
Trace plot
sample value
Country-level: N segments
Log density sq.
frequency
intercept
log GDP pc
internet
log pop
governance
Trace plot
sample value

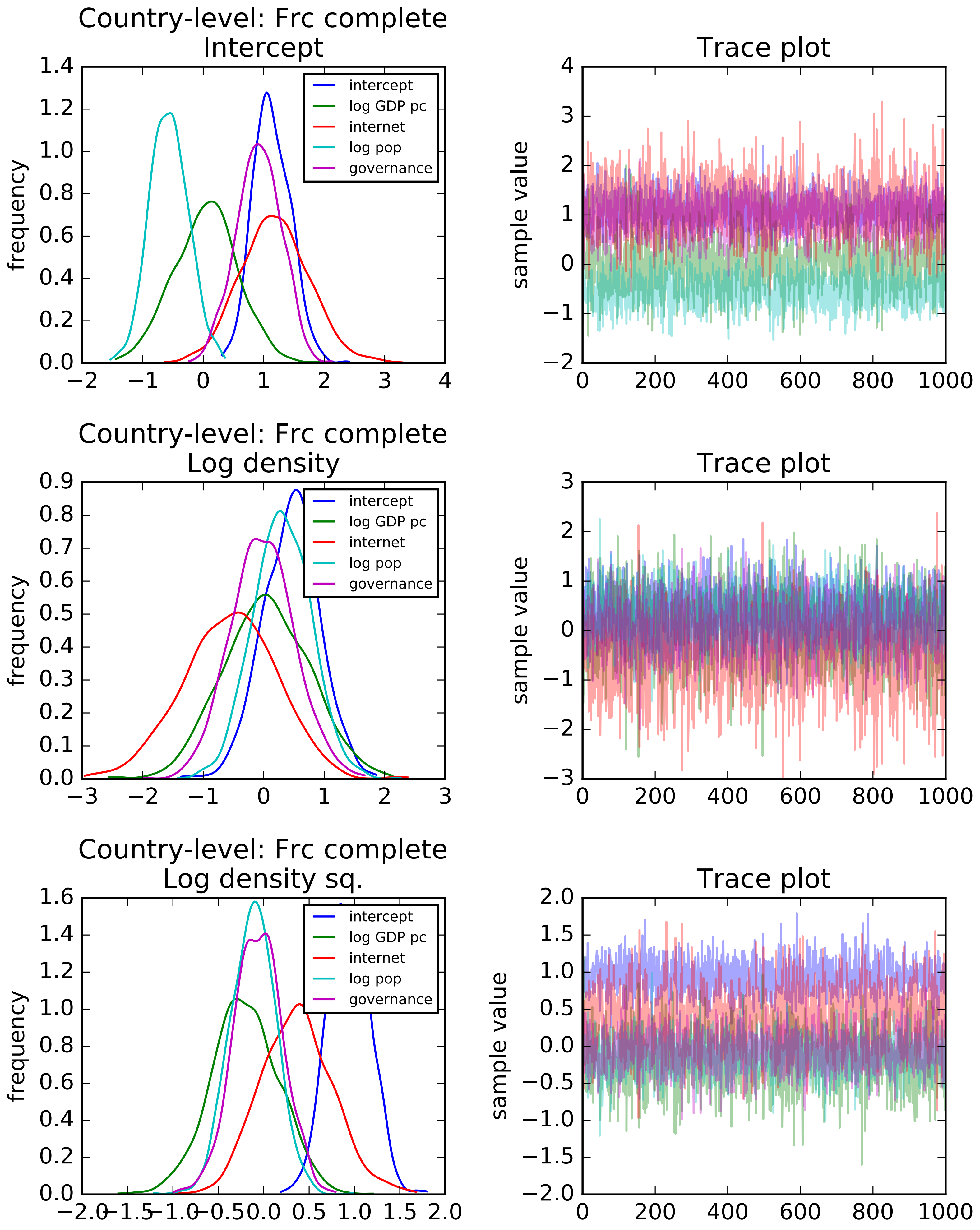

Country-level: Frc complete
Intercept
frequency
intercept
log GDP pc
internet
log pop
governance
Trace plot
sample value
Country-level: Frc complete
Log density
frequency
intercept
log GDP pc
internet
log pop
governance
Trace plot
sample value
Country-level: Frc complete
Log density sq.
frequency
intercept
log GDP pc
internet
log pop
governance
Trace plot
sample value

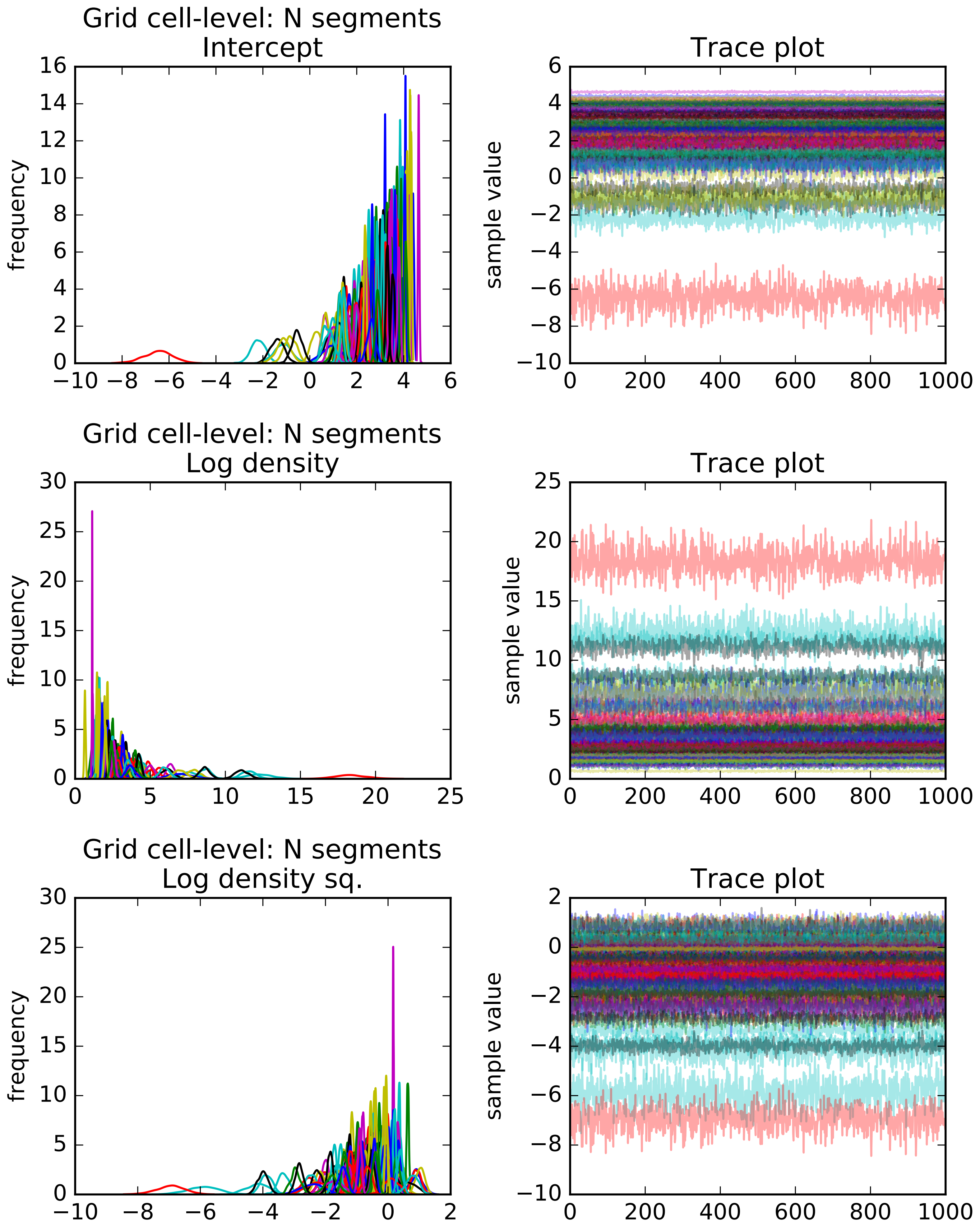
Grid cell-level: N segments
Intercept
frequency
Trace plot
sample value
Grid cell-level: N segments
Log density
frequency
Trace plot
sample value
Grid cell-level: N segments
Log density sq.
frequency
Trace plot
sample value

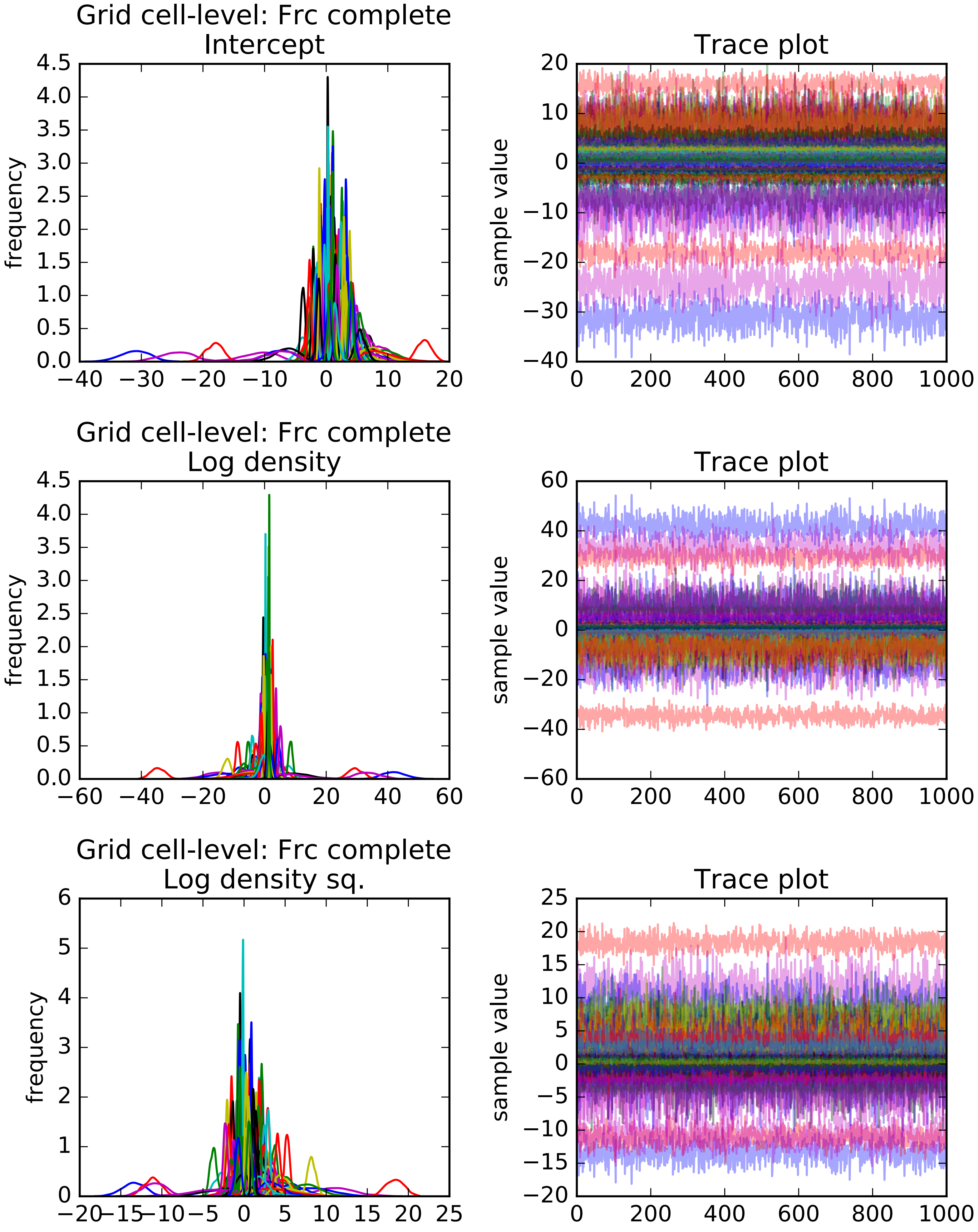

Grid cell-level: Frc complete
Intercept
frequency
Trace plot
sample value
Grid cell-level: Frc complete
Log density
frequency
Trace plot
sample value
Grid cell-level: Frc complete
Log density sq.
frequency
Trace plot
sample value

I Data release

We provide both the data set corresponding to the published paper, and a 2017 update. In the 2017 update, both our methods find an 89% completion rate for roads, overall, planet-wide. Within each, three separate components are provided:

1. a compiled country-level dataset
2. the visual assessment data
3. the OpenStreetMap history, aggregated by density, national and sub-national boundaries.

These data are openly available at `https://alum.mit.edu/www/cpbl/publications/PLOS2017roads`

J 2017 update

We updated our analysis to use the OSM contribution history up to May 2017. Using these updated data, both our methods find an 89% completion rate for roads, overall, planet-wide. Below are our fits corresponding to Figure 4 in the paper.

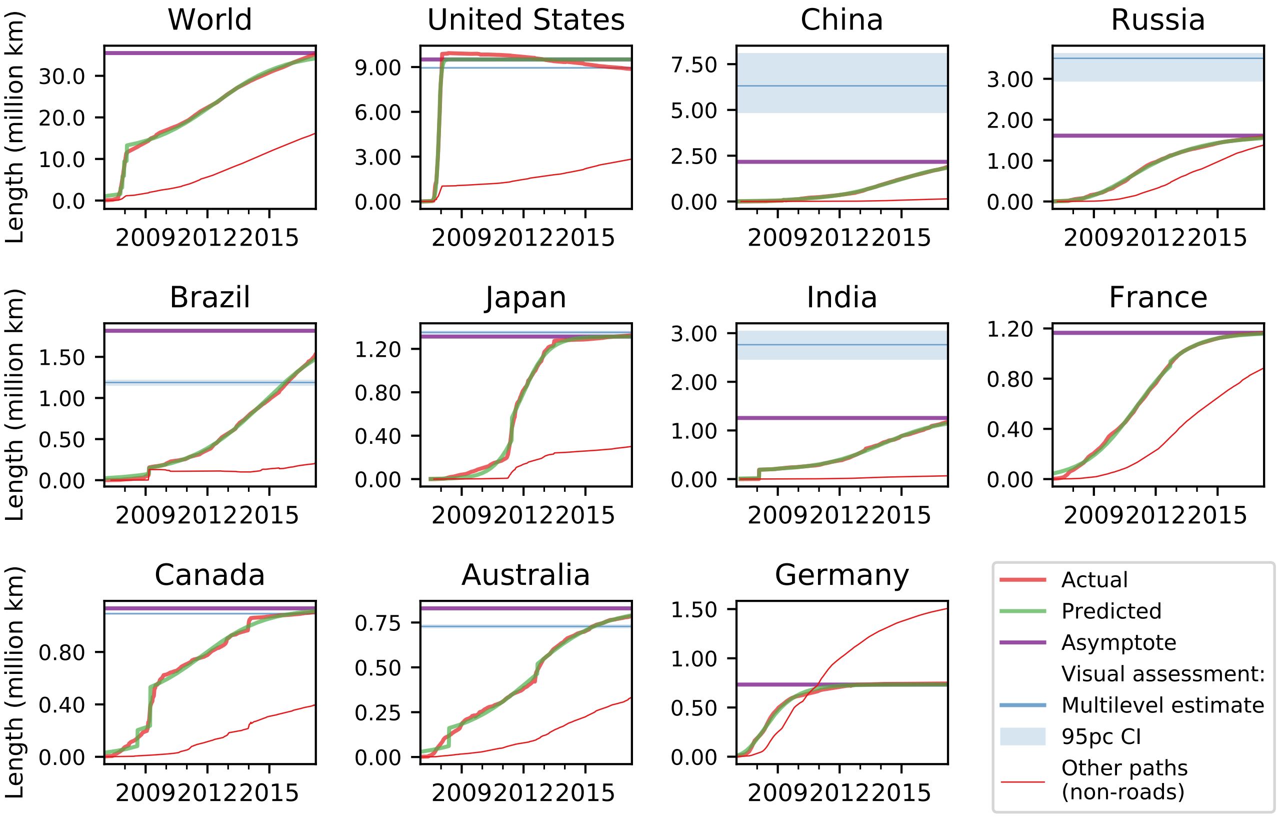

K Open source Python code

Our code to reproduce the data and analysis is released under the GNU General Public License v3.0 as an open source project, permanently available at:

`https://alum.mit.edu/www/cpbl/publications/PLOS2017roads/osm-completeness`

A direct link to a description of the code is at:
`https://github.com/cpbl/osm-completeness/blob/master/README.md`

L Citation

For any use of data or code, cite the original PLOS One paper

Barrington-Leigh, Christopher and Millard-Ball, Adam (2017), "The world's user-generated road map is more than 80% complete," [citation to be updated...]

M Contact

For further questions, please contact:

Chris Barrington-Leigh, McGill University
Adam Millard-Ball, University of California, Santa Cruz
